# Improvements to previous algorithms to predict gene structure and isoform concentrations using Affymetrix Exon arrays

**DOI:** 10.1186/1471-2105-11-578

**Published:** 2010-11-26

**Authors:** Miguel A Anton, Ander Aramburu, Angel Rubio

**Affiliations:** 1 CEIT and TECNUN, University of Navarra, San Sebastián, Spain

## Abstract

**Background:**

Exon arrays provide a way to measure the expression of different isoforms of genes in an organism. Most of the procedures to deal with these arrays are focused on gene expression or on exon expression. Although the only biological analytes that can be properly assigned a concentration are transcripts, there are very few algorithms that focus on them. The reason is that previously developed summarization methods do not work well if applied to transcripts. In addition, gene structure prediction, i.e., the correspondence between probes and novel isoforms, is a field which is still unexplored.

**Results:**

We have modified and adapted a previous algorithm to take advantage of the special characteristics of the Affymetrix exon arrays. The structure and concentration of transcripts -some of them possibly unknown- in microarray experiments were predicted using this algorithm. Simulations showed that the suggested modifications improved both specificity (SP) and sensitivity (ST) of the predictions. The algorithm was also applied to different real datasets showing its effectiveness and the concordance with PCR validated results.

**Conclusions:**

The proposed algorithm shows a substantial improvement in the performance over the previous version. This improvement is mainly due to the exploitation of the redundancy of the Affymetrix exon arrays. An R-Package of SPACE with the updated algorithms have been developed and is freely available.

## Background

Alternative splicing (AS) is the process by which a single gene is able to express several proteins that may have diverse or even antagonistic functions. The mature mRNA that corresponds to the different proteins for the same gene is called transcript isoform or isoform in short. Some AS is related to development, tissue differentiation, etc. Other AS events are pathological and are associated with various diseases including cancer. Many of them are yet unknown. The analysis of disease specific alternative splicing and its molecular consequences is promising and can be used to find new diagnostic, prognostic, predictive, and therapeutic tools [1].

Some microarray companies (Affymetrix, Jivan Biotechnology and Exonhit) provide arrays designed to capture alternative splicing events. There are also custom arrays that use the Agilent platform [2]. The main difference between these technologies is that Affymetrix only includes exon probes (complementary sequences to each one of the known transcribed exons of a given gene) and Jivan, Exonhit or customized Agilent arrays also include junction probes (a segment of complementary nucleotides for each of the two sides of a known exon-exon junction in the mature mRNA of the gene). Affymetrix has recently developed an experimental array that also includes junction probes. This work focuses on Affymetrix exon arrays. There are a number of studies that propose different methods to deal with these arrays [2-8]. The procedure to extract a signal value from a set of probes in an Affymetrix array (including exon arrays) can be divided into three stages: background removal, normalization (to equalize the conditions among all the experiments) and summarization (to provide a single concentration measurement that represents all the signals in a set of probes that corresponds to a particular analyte). Usually the proposed methods for exon arrays [9,10] are modifications of the algorithms already used in expression arrays.

Algorithms for removing background and performing normalization in expression arrays can be reasonably applied to exon arrays. However, the summarization step has some special characteristics that make it difficult to apply these methods. To clarify this assertion, it is important to distinguish that, in exon arrays, there are three ways to group the probes: by genes (to measure gene expression), by transcripts (to measure transcript expressions) and by exons (to measure the exon expression). Affymetrix [11] and Brainarray [12] provide different updated versions of the Chip Definition Files (CDF) to do the grouping using genes, transcripts or exons and using different identifiers for each of them. Our work focuses on transcripts.

In a summarization step it is implicitly assumed that there is no cross-hybridization between probes that belong to different sets (or at least in most of the probes). However, if the probes are grouped by transcripts and a gene presents several isoforms, there will be many probes that hybridize with several transcripts of the same gene. To make matters worse, specific probes for a particular transcript (that do not show cross-hybridization) may be excluded if the computation is a robust summarization method. The reason is that, since their behavior is different from other probes in the set, their values are not used in the summarized signal. As many as 85% of the exons in genes with several isoforms are shared by several transcripts. It is not advisable to use standard expression algorithms to exon arrays grouped by transcripts. In the seminal study of Wang *et al*. [13] this problem is stated and solved by using a deconvolution algorithm assuming that the structure of the gene is known. Recently, a work that also deals with transcript concentration estimation has been proposed [14]. For some gene structures there is a deconvolution ambiguity and additional constrains may be necessary to resolve transcript expressions [15,16].

To deal with the problem of quantifying transcripts, the authors and some colleagues [17] designed the algorithm "Splicing Prediction and Concentration Estimation" (SPACE) based on Non-negative Matrix Factorization (NMF). SPACE algorithm was used to predict the structure for each particular gene and quantify the concentration of each particular transcript including unknown isoforms. SPACE was designed to be used with arrays with either exon or junction probes. The special characteristics of the Affymetrix arrays drove us to include some refinements to this algorithm. They are (1) detection and removal of the outliers in the probe data, (2) estimation of the number of transcripts of a gene in a set of samples, (3) selection among the possible solutions which is the most suitable for splicing data (NMF solution is not unique) and (4) use of the information of probesets to correct errors in the predicted structure of the genes. In the results section, the improvements for simulated data are shown. The accuracy of predicted structures and concentrations validated by PCR for real datasets is also tested. In addition to this, the SPACE algorithm with the improvements described here, has been ported to R (initially it was written in Matlab).

## Results and Discussion

The improvements described in the methods section to the initial SPACE algorithm were applied to both synthetic and real microarray datasets. Each of the datasets are described in the following sections.

### Synthetic dataset

Around 600 genes (selected randomly) that show alternative splicing were simulated. Probe signals for this synthetic dataset are proportional to the sum of concentrations of the transcripts that share the probe, in turn, this proportional constant, the affinity, was estimated from the values of each probe in an experiment for different tissues provided by Affymetrix. These affinities are estimated using dChip. This method assumes that the signal is proportional to the product of the affinities of the probes and the concentration of the gene. Once the estimated concentration of the gene is known it is quite straightforward to get the affinities. Another equivalent possibility is to get the affinities using the first eigenvector of the SVD decomposition of the probe data matrix for each gene. Since dChip assumes that there is only one isoform per gene, these affinities are not accurate but sufficient to perform a simulation. Transcript concentrations are constructed by drawing random numbers that follow a uniform distribution across samples and multiplying them by a proportional factor also drawn from a uniform distribution. This approach simulates that there are some transcripts that are systematically more expressed than others in a gene.

The property *G *matrix for each of the genes was built using the information provided by Brainarray [12]. *G *is an indicial matrix (*g_ij _*= {0, 1}) that relates probes with transcripts, i.e. it discerns whether a probe is included in a transcript or not. The size of this matrix is *probes transcripts*. Following the deconvolution model proposed by Wang *et al*. [13], the simulated probe signal matrix (*probes samples*) was built by multiplying the three matrices (*Y *= *A⋯G⋯T*). The underlying reasoning is that the signal of each probe is proportional to its affinity times the sum of the concentrations of the transcripts that hybridizes against it. Finally, noise (both multiplicative and additive) was added. This noise was computed so that it is similar to the noise present in real data. In turn, the noise in real data was estimated by using the residuals of the dChip summarization model.

This simulated dataset are 600 genes with known structure (obtained from Brainarray), known number of transcripts (consequence of the structure) and known concentrations (generated using random numbers) plus noise that mimics the real distribution.

The SPACE algorithm includes a NMF step. The NMF is computed iteratively. Each gene is iterated 3000 times to achieve convergence.

The results are presented for estimation of the number of different transcripts in a given mixture, structure prediction of each of these transcripts and estimation of their concentration.

#### Prediction of the number of transcripts

**Figure 1:**
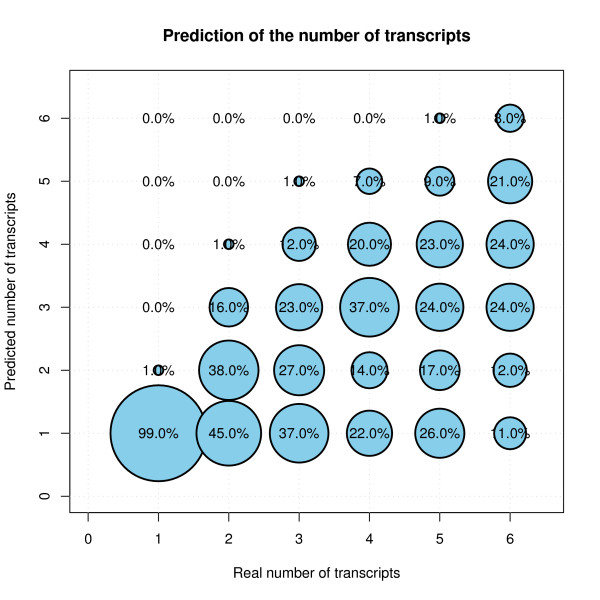
**Proportion of predicted number of transcripts in a simulation performed with 600 genes (synthetic data)**. The 600 genes used were randomly selected from the human genome with one to six transcripts. Annotated transcript structures in Brainarray version 11 CDFs were used to make the synthetic data for each gene. Probe affinities were estimated from real data (Affymetrix sample dataset for human tissues). Both additive and multiplicative noise mimics the noise present in real datasets. Transcript concentrations have been randomly assigned from a uniform distribution across samples multiplied for a proportional factor (also from a uniform distribution) between transcripts, the resulting concentrations are such that some transcripts present lower concentrations in all samples compared with the concentrations of the most significant transcript of the same gene. The area of each circle represents the proportion of times the corresponding predicted number is chosen by the algorithm for a given number of transcripts. Genes with a single transcript are wrongly predicted to have AS (several transcripts) only 1%.

The number of transcripts was estimated using a modification to a method proposed by Owen and Perry [18]. This method performs a partition of the initial data matrix (*Y*) into four submatrices and estimates one of these submatrices using the remaining three submatrices. Changing the role of each of the submatrices, it is possible to predict the whole initial data using approximately 75% of these data. The estimation is performed by means of using NMF (or partial SVD) and is done considering different internal dimensions for the factorizations into consideration. The estimation process was repeated a number of times for different partitions (250 in our case). Afterwards, we selected the minimal internal dimension of the factorization (number of transcripts) that provides an error distribution whose median is not statistically larger than the distribution of the error of the internal dimension with the minimum error (using a Wilcoxon test). Figure 1 shows the performance of this estimation. It compares the predicted number of transcripts (using only the simulated probe level data) with the number of transcripts in Ensembl (using Brainarray cdfs).

This simulation shows that the estimation of the number of transcripts is a difficult task even for synthetic data since, for this level of noise, the predicted number of transcripts is usually incorrect. Nevertheless, if the proposed method is used simply to predict splicing, it has a very low false positive rate (FPR), i.e. only 1% of the genes that have a single transcript are predicted to have alternative splicing. The false negative rate (genes with several transcripts that are predicted to have one transcript) is on average about 30%. This false negative rate (FNR) is (among others) a consequence of some simulated transcripts having very low concentrations in all samples, in comparison with the most significant transcript of the same gene. We also performed a simulation assuming that all the transcripts follow an uniform distribution and FNR improves (data not shown).

The Figure S3 in Additional file 1, compares the performance of the number of transcripts predictions using the new and the previous version of the algorithm on the same simulated dataset. The new version estimates the number of transcripts more accurately.

#### Structure prediction

SPACE algorithm was applied to each of the genes in the synthetic dataset. The algorithm provided a continuous estimation $\tilde{G}$ of the property *G *matrix (see methods section).

The $\tilde{G}$ matrix has all its values bounded between zero and one. On the other hand, the original *G *matrix is binary (i.e. a probe does or does not hybridize against a transcript). It would be desirable to quantify the ability to predict the *G *matrix using $\tilde{G}$.

Changing a threshold *th*, it is assumed that a particular entry of $\tilde{G}$ is 1 (the probe is predicted to hybridize against a particular transcript if the corresponding entry is over *th*) or 0 (the probe is predicted not to hybridize against this particular transcript if the corresponding entry is below *th*). Comparing the corresponding entries from *G *and ($\tilde{G}$ >*th*), there are true positives (TP, corresponding entry is 1 in both matrixes), true negatives (TN, the entry is 0 in both matrixes), false positives (FP, the entry is 0 in *G *and $\tilde{G}$ is over *th*) and false negatives (FN, the entry is 1 in *G *and $\tilde{G}$ is below *th*). These values (using different thresholds) can be used to construct the ROC or compute other performance figures. Each gene has its own ROC curve. Summing up each of TP, TN, FP and FN for all the genes it is possible to get an estimation of the overall performance.

It is important to point out that there is always an indetermination in the order of the transcripts: the columns of *G *and $\tilde{G}$ for a perfect prediction can have a different order. Before performing the previous computations, $\tilde{G}$ is resorted so that it becomes more similar to *G*. The same sorting is applied to the rows of the concentrations matrix.

**Figure 2:**
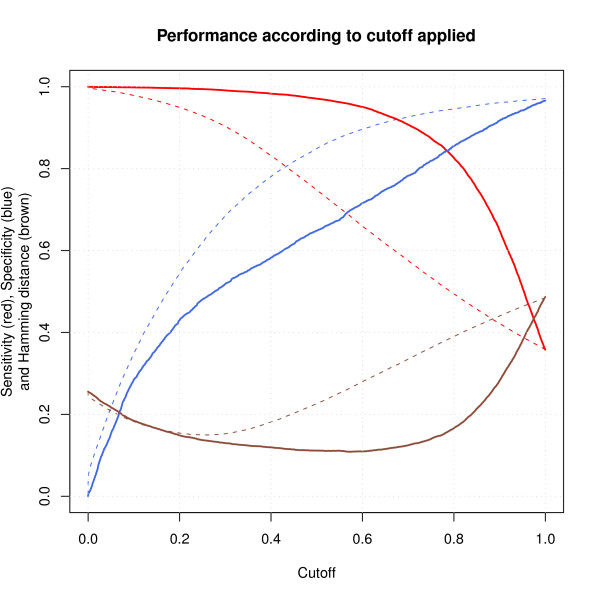
**Performance of the structure prediction**. Sensitivity (SN), specificity (SP) and Hamming distance (HD) of the structure prediction obtained in the simulations for different values of applied threshold. The dashed lines show the performance of SPACE before applying improvements and the continuous lines after applying Adapt algorithm and coherence correction. The Hamming distance error rate was calculated in the form of *HD *= (*FP *+ *FN*) /*N*, i.e. the proportion of probes that are erroneously predicted. The sensitivity (or true positive rate, *TPR*) was calculated as *SN *= *TP *= (*TP *+ *FN*), i.e. the proportion of probes that are predicted to hybridize against a transcript and in fact do hybridize. Finally, the specificity (or one minus the false positive rate, *FPR*) was calculated as *SP *= *TN/*(*TN *+ *FP*), i.e. the proportion of probes that are predicted NOT to hybridize against a transcript and in fact do not hybridize. The x-axis shows the threshold in the estimated $\tilde{G}$ matrix to decide whether a probe hybridizes or not. The threshold that provides the minimum Hamming distance (overall error) is 0.5. A threshold near 0.8 provides the same sensitivity and specificity, both equal to 0.85; such value represents an error at least 25% lower than the crossing point for SPACE without improvements.

Different error figures in the prediction are represented in Figure 2. It includes the Hamming distance, the sensitivity and the specificity for different thresholds over the $\tilde{G}$ matrix. The dashed lines belong to the SPACE algorithm without improvements and the continuous lines to SPACE with improvements.

In figure 2, it can be observed that the optimum threshold for the Hamming distance (overall error) is about 0.5. The new algorithm has a lower hamming distance than previous one. If the threshold that provides the same sensitivity and specificity (the crossing point) is set, both the one and zero values have the same reliability. In this case, we consider accidental inclusion as equally undesirable as accidental exclusion of an exon. This threshold is close to 0.8. The sensitivity and specificity of SPACE in the crossing point is equal to 0.85; such value represents an error 25% lower than the crossing point for SPACE without any improvement.

#### Concentration estimation

We scaled the columns of the *W *matrix to make its structure more similar to *AG *matrices. This scaling, different for each of the columns of *W *is possible because of the non-uniqueness of the NMF. The initial SPACE algorithm included a heuristic to obtain a "filled" G matrix that is also used in the improved one before the scaling. The estimated concentrations of the transcripts with and without the adaptation of *W *matrix in SPACE were compared. In figure S4 in Additional file 1, the mean average error (MAE%) for different genes used in the simulations is shown. In that figure, it can be observed that adaptation improves the estimation of the concentrations. In most of the cases the diminution in the estimated error is statistically significant.

### Real datasets

SPACE was also applied to several datasets downloaded from GEO [19]. All of them have available CEL files to perform all the steps of the analysis and the corresponding papers include validation of the results using RT-PCR. These datasets are: Affymetrix sample dataset of human tissues. It contains 11 tissues with 3 replicates of each tissue along with several mixtures of three tissues (heart, testes and cerebellum). A recent study made by de la Grange *et al*. [8] includes RT-PCR validation for some genes in that dataset; GSE9385 [20] (a study of glial brain tumors in humans); GSE9372 [21] (a comparative study of the relationships between genotype and alternative splicing in humans); GSE11344 [22] (change in splicing patterns after PTB depletion in mice); and GSE8945 [23] (a study on the effect of the hnRNP L protein on alternative splicing).

The Table S1 in Additional file 2, summarizes the results of the predictions for each of the datasets. The table includes the following fields: dataset, gene, RT-PCR band according to Fast-DB, number of isoforms between primers using PCR, predicted number of isoforms between primers using SPACE, number of isoforms between primers according to Ensembl 51, predicted total number of isoforms, total number of isoforms in Ensembl 51, coherence of predicted structure with PCRs, coherence of estimated concentrations with PCRs and a comment of the results.

#### Affymetrix dataset

**Figure 3:**
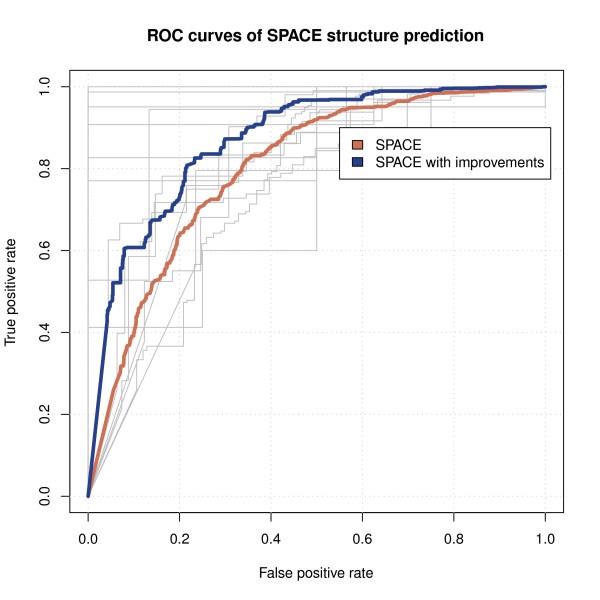
**ROC curves of SPACE structure prediction (Affymetrix sample dataset of human tissues)**. ROC curves that test SPACE performance for structure prediction. They measure the concordance of the predictions with the transcript structures that appear in Ensembl release 51. The y-axis shows the sensitivity (the proportion of probes that are said to hybridize against a transcript and in fact do) and in the x-axis 1 - specificity is shown (the proportion of probes that are said not to hybridize against a transcript and in fact they do not). A total of 15 genes that present alternative splicing between tissues and were validated by RT-PCR (figures 3 and 4 of de la Grange *et al*. [8]) were chosen to make the ROC curves. Two genes SNX13 and IDE (also validated in the same figures) were not included because alternative splicing events shown in the PCRs do not appear in Ensembl release 51. Another two genes ABLIM1 and MICAL2 were validated in two different splicing events. The number of transcripts of each gene in the tissue dataset was estimated from the PCR figures. The first ROC curve corresponds to the prediction without taking into account the improvements described in this paper. The second ROC curve was obtained after applying Adapt algorithm and structure coherence correction (probes within the same exon or exon part must have the same hybridization pattern). The ROC curves of each of the 15 genes are shown in grey as background.

ROC curves have been made to test SPACE performance for the structure prediction of alternatively spliced isoforms. As already stated, SPACE predicts the overall structure of a gene whereas RT-PCR experiments can identify only the events that occur between the included primers. In order to test the quality of SPACE predictions we made the following assumption: if the results of a RT-PCR within a gene in a dataset is compatible with Ensembl structure, we assume that the Ensembl structure is correct. In figures three and four of the study made by de la Grange *et al*. [8] a total of 17 genes that present alternative splicing between tissues were validated by RT-PCR (some of them in more than one splicing event). Two genes SNX13 and IDE were excluded because alternative splicing events shown in the PCRs do not appear in Ensembl release 51. Assuming that the gene structures of the remaining 15 genes in Ensembl are correct, we use these structures for each gene as "ground truth" and computed the ROC curves shown in Figure 3. The number of transcripts of each gene in all samples was estimated from the PCR figures. The number of transcripts was also estimated by the proposed algorithm. The estimated number of transcripts was usually larger than the indicated by PCRs. Two ROC curves were constructed. The red ROC curve indicates SPACE performance without any improvement. The black ROC curve was estimated applying the improvements described in the methods section.

**Figure 4:**
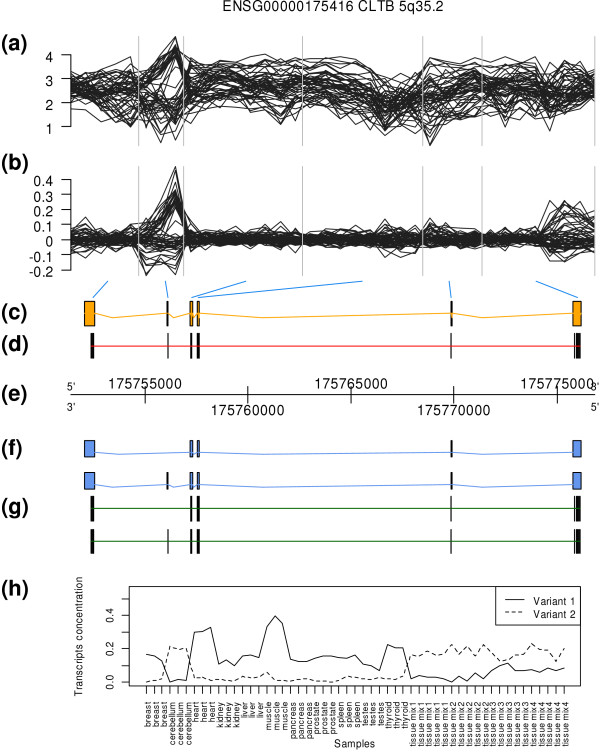
**Results for CLTB gene (Affymetrix sample dataset of human tissues, reverse strand)**. (a) Log of the intensities for all the probes within the gene. Each line corresponds to a different sample. (b) Residuals of the RMA normalization model. The residuals for some exons are much larger than for others. One of these exons is predicted as a cassette alternative splicing event (skipped exon). The vertical bars detach the different exons (or piece of exons) that correspond to each group of probes. (c) Structure of the CLTB gene, i.e. the exons that appear in any of its transcripts. (d) Locations of the Exon probes. It can be observed that each exon has one or several probes mapped to them. (e) Genomic positions of the probes. (f) Structure of the different transcripts of CLTB gene in Ensembl release 51. Two different transcripts are represented. (g) Predicted structure using SPACE. The predicted number of transcripts is two and their predicted structure is shown. Predicted transcripts match exactly the Ensembl annotated ones. (h) Estimated concentration of each of the two transcripts (variant 1 and 2) in each sample. Variant 2 is predominant in cerebellum tissue as shown by RT-PCR in [8]. These graphs were generated using GenomeGraphs [30].

The analysis for CLTB gene is shown in Figure 4. SPACE algorithm was run to predict the structure of two alternatively spliced isoforms. Their predicted structure in panel (g) shows a cassette event that is characteristic of cerebellum. Predicted structure is the same than annotated structure in Ensembl release 51 shown in panel (f). The predicted concentrations of isoforms clearly show that variant 2, which includes the cassette exon, is exclusive of nervous tissue. These results match RT-PCRs provided in de la Grange *et al*. [8]. Results for other genes in [8] (and all the other studies) are shown in the additional material (Additional file 1).

#### Glial tumors

**Figure 5:**
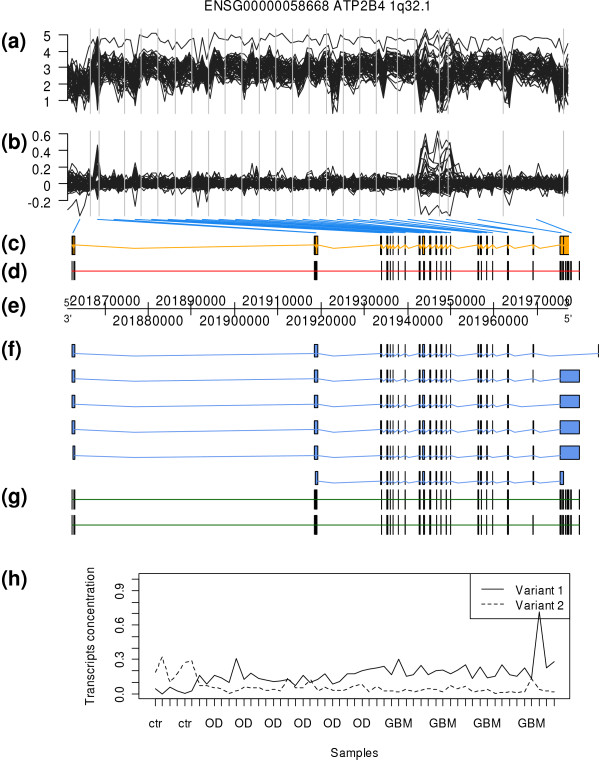
**Results for ATP2B4 gene (data from GSE9385)**. The content of each panel is explained in figure 4. (f) In Ensembl release 51 there are six different annotated transcripts. (g) The predicted number of transcripts using SPACE is two and their predicted structure is shown. A cassette event of one exon toward the 5' end of the gene is clearly identified. (h) Estimated concentration of each of the two transcripts (variant 1 and 2) in each sample. Samples 1 to 6 correspond to normal tissue, samples 7 to 32 to oligodendroglioma and samples 33 to 55 to glioblastoma multiform. The results for the estimation of the concentrations are the reverse of RT-PCR results.

In the study of glial brain tumors in humans made by French *et al*. [20], the authors provide PCRs for 11 genes. The analysis for ATP2B4 gene is shown in Figure 5. SPACE algorithm was run to predict the structure of two isoforms. Their predicted structure in panel (g) shows a cassette event that involves one exon towards the 5' end of the gene. The estimated concentrations of both transcripts (variant 1 and 2) across all samples are shown in panel (h). Variant 1 (short isoform) is almost non-existent in samples 1 to 6 (normal tissue). In samples 7 to 32 (oligodendroglioma), variant 2 has a larger concentration than variant 1. In samples 33 to 55 (glioblastoma multiforme), this difference in concentration is even more apparent. These results are not consistent with the PCRs shown in [20]. In this study, the PCR shows that the long isoform is expressed only in GBM cells whereas the short isoform is expressed similarly in the three groups. This error in the predicted concentration may be owed to the lack of identifiability of this particular gene structure [15,16].

#### Genotype and alternative splicing

**Figure 6:**
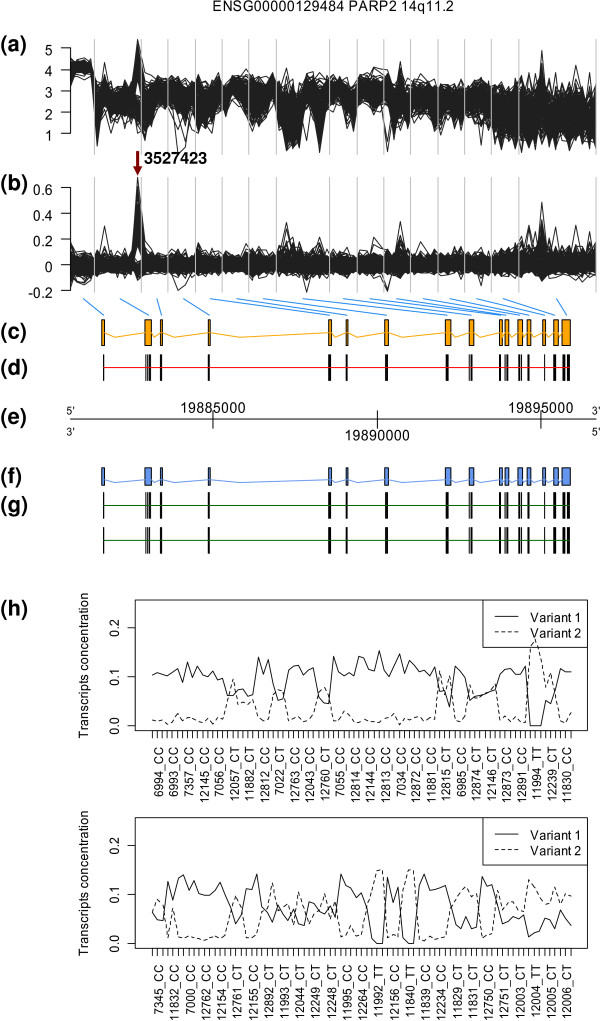
**Results for PARP2 gene (data from GSE9372)**. According to Kwan *et al*. [21], this gene shows AS related to a particular SNP in probeset 3527423 and it has a new variant with its second exon shortened. The content of each panel is explained in figure 4. (b) Residuals of the RMA normalization model. For some exons there are residuals much larger than for others, as at the end of the second exon where the probeset 3527423 is located. (g) There is a splicing event at the end of the second exon which correspond to probeset 3527423 in transcript variant 2. Transcript variant 1 matches Ensembl annotated transcript. (h) Estimated concentration of each variant with three replicates per transcript. These concentrations match RT-PCR results perfectly.

In the comparative study of the relationships between genotype and alternative splicing in humans, the validation done with PARP2 gene is shown in Figure 6. This gene, according to Kwan *et al*. [21] study, shows an alternative splicing event related to a particular SNP in probeset 3527423. In Ensembl release 51, this gene is annotated as a single transcript. Two transcripts were predicted by SPACE algorithm and their predicted structure is shown in panel (g). Predicted transcript variant 1 is equal to the annotated transcript in Ensembl. Predicted transcript variant 2 shows the lack of probeset 3527423. In panel (h), the estimated concentrations of the two predicted transcripts are shown. There are three replicates of each sample and the estimated concentrations match perfectly PCR results for both splicing variants in each sample.

In this particular case, the coherence algorithm would miss this alternative splicing event because the Ensembl annotation does not include this variant.

#### PTB depletion in mice

**Figure 7:**
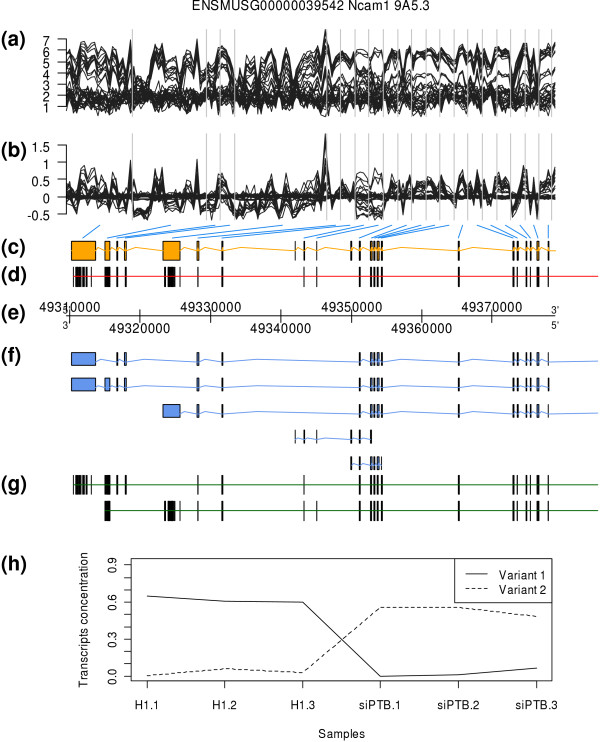
**Results for Ncam1 gene (data from GSE11344)**. The content of each panel is explained in figure 4. (b) Residuals of the RMA normalization model. It can be observed that for many exons there are residuals that are much larger than expected. This fact indicates the presence of many splicing events. (d) Most of the exons have several probes mapped to them. However, some exons (in the middle close to the 49.34 Mb position) are not represented by any probe. Any splicing event related to these exons cannot be detected. (g) Predicted structure using SPACE of two transcripts of this gene. The first transcript variant 1 is related to the first Ensembl release 51 transcript. The second transcript variant 2 is related to third Ensembl transcript. A few exons are misassigned to these transcripts. (h) Estimated concentrations of each of the two transcripts (variant 1 and 2) in each sample. Samples 1 to 3 correspond to normal tissue where the variant 1 is predominant. Additionally, variant 2 is predominant in samples 4 to 6 that present PTB depletion. These results are in concordance with RT-PCR results.

In the analysis of predetermined transcript inclusion levels after PTB depletion in mice made by Xing *et al*. [22], the obtained results for Ncam1 gene are shown in Figure 7. This gene has five different transcripts annotated in Ensembl release 51 as shown in panel (f), two of which are very short compared to the length of the gene. Two transcripts were predicted by SPACE and their predicted structure is shown in panel (g). The first predicted transcript variant 1 is very similar to the first Ensembl transcript. The second predicted transcript variant 2 is also similar to the third Ensembl transcript. Some probes that belong only to the short Ensembl transcripts are incorrectly assigned to the larger predicted transcripts and the second exon is also misassigned to the predicted variant 2. In panel (h), the estimated concentrations of the two predicted variants are shown. Variant 1 is predominant in the samples 1 to 3 (normal tissue) and variant 2 is predominant in the samples 4 to 6 (tissue of mice with PTB depletion). These concentrations are in concordance with PCRs.

### Discussion

We have described the different improvements on SPACE: outlier detection and correction; prediction of the number of transcripts; the adaptation of the factors of the NMF to provide a structure closer to the reality; and the exploitation of the redundancy of the Affymetrix probes.

All the improvements were tested on synthetic data mimicking the real data (affinity, structure and noise were taken from real data). Suggested changes improved error rates by about 25%. We have also tested our algorithms in RT-PCR validated data. The ROC figures document a specificity and sensitivity of around 75% in the case of structure prediction. Concentration estimates to a large extent mirrored PCR images in the cited studies.

However, when applying SPACE (with the suggested improvements) to real data we faced some problems worth noting. For example, SPACE algorithm assumes that all the probes hybridize against at least one of the transcripts. In exon arrays there are many probes (probes related with erroneously predicted exons) that do not hybridize against mRNA, this happens even in the core annotated probes. Additionally, many of the probes in exon arrays do not perform well or at least there are probes within the same exon that do not correlate well with other probes of that exon.

Structure prediction improves with careful selection of the probes, e.g. by identifying and discarding probes that do not hybridize or that cross-hybridize to different parts of the genome (a problem addressed by Kapur *et al*. [24]). Background acts as an offset for the probes in the array. Even though the applied background removal algorithm [25] behaves better than the standard RMA version, further improvements can be made at this point. This problem is attenuated but not completely solved when using Brainarray [12] CDF files for genes, transcripts and exons. There are several papers [4,22,24] that address these questions and provide different methods to select the probes.

Assessment of the performance of the SPACE algorithm for real datasets required some sort of "ground truth". In this paper, only genes with RT-PCR validated splicing events in each dataset were used for validation. It should be noted that PCRs only assess splicing events that appear between the primers, while SPACE algorithm reconstructs the whole structure of each transcript. For the validated gene set, transcript structures from Ensembl release 51 were used as reference. Inaccuracies in the Ensembl annotation would lead to an underestimation of the validation rates. In Figures S1 and S2, Additional file 1, the concordance between SPACE predictions and Ensembl release 51 annotations was tested for a set of 1600 randomly selected genes. The performance decreased compared to genes that have RT-PCR evidence of alternative splicing. Additionally, the concordance of the estimated concentrations of each isoform in each sample with the PCRs shows the accuracy of the predictions.

The redundancy of the Affymetrix probes in exon arrays was exploited to detect and correct errors in the structure. Probes that belong to the same exon or probeset (in the case of exons that differ between transcripts) must all be present or absent in a particular transcript. After applying this correction, both sensitivity and specificity improved significantly in real and simulated datasets. However, if a new transcript had a splicing event (previously unknown) that did not follow the probeset grouping (a new alternative donor site, for example), the prediction would be incorrect, as the algorithm would treat the probeset as a whole.

Variability between samples provides SPACE algorithm with more information that leads to better results in the prediction of structure. For example, a normal vs tumor experiment that consists of several technical replicates of the same samples provides less information than an experiment with 11 different tissues as the Affymetrix sample dataset. Additionally, the number of transcripts present in all samples is also important to the algorithm performance. If there are more than 4 or 5 transcripts SPACE performance greatly decreases.

Using the Wang deconvolution model, it would be possible to estimate transcript concentrations provided that the gene structure (*G *matrix) is known. However, there is a deconvolution ambiguity that only depends on the gene structure [15,16]. This ambiguity may provide different valid solutions to the Wang model for some genes with the same probe intensities. SPACE is also sensitive to this ambiguity. However, as shown in the simulations, it is able to predict the structure and estimate the concentrations with reasonable accuracy. She *et al*. [15] suggest a method to deal with this ambiguity by estimating the mean affinity of groups of probes. This is a priori information which can also be included in the SPACE algorithm. It is also possible to test later whether the estimated *G *matrix has this ambiguity or not.

All the described algorithms have been included in an R-Package and added as the Additional file 3. A script to test the functions is also included as Additional file 4. The source code and the manual of the package are included as Additional file 5 and Additional file 6 respectively.

## Conclusions

We have proposed a method to predict the structure and estimate the concentration of transcripts using Affymetrix exon arrays. We have included several improvements over our previous work, namely; outlier identification and removal, improved estimations of the number of transcripts, adaptation of the NMF factors to mimic the proposed model and exploitation of the redundancy of the exon arrays that include several probes per exon. Simulations show that the error figures improved by applying these methods. The algorithms have been validated in real datasets.

## Methods

**Figure 8:**
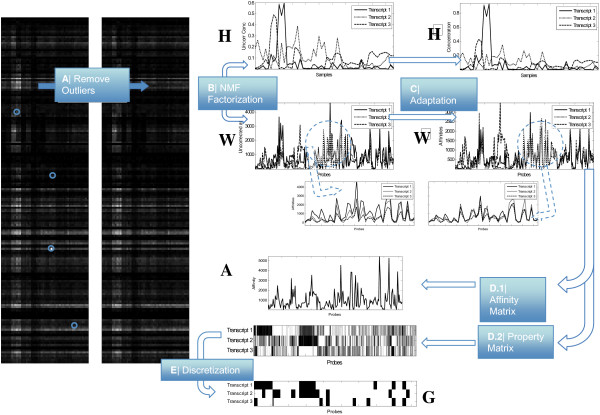
**Scheme of the different algorithms in the SPACE method**. The figure depicts the different proposed stages that are performed after the background removal and normalization across the arrays. The initial input to the algorithm is the probe expression matrix for a particular gene (in this case the WNK1 gene). The selected experiment is a set of arrays hybridized against different tissues provided by Affymetrix. Probe signal matrix may have some outliers that are identified and corrected in stage A (outlier removal) of the method. In stage B (NMF factorization), the corrected matrix is factorized into two matrices *W *(related to concentration) and *H *(related with the structure). NMF factorization is not unique. We select the factorization whose $\tilde{W}$ matrix is closer to the *AG *factorization suggested by Wang *et al*. This adaptation is performed in stage *C*. It can be seen in the zoomed figures that the $\tilde{W}$ matrix have columns that are closer to each other. Once we have the $\tilde{W}$ matrix it can be factorized again into two matrices: a matrix of affinities (step D.1) and a continuous $\tilde{W}$ = $A\tilde{G}$ property matrix $\tilde{G}$. Finally, the discretization process (step E) provides a binary structure of the *G *matrix that is compatible with the characteristics of the exon arrays, i.e., the probes that belong to the same exon probeset have the same hybridization pattern.

The different steps of the suggested method are depicted in Figure 8. These steps are: outlier removal, factorization, adaptation of the *W *matrix (Adapt algorithm) and structure correction by coherence.

### Normalization and background removal

The Robust Multiple-array Average (RMA) [26] procedures for background removal and quantile normalization were used. Instead of using the standard RMA background method, we used [25] (developed by the same group), an improvement over the previous algorithm. These methods are quick and efficient and can be readily applied to exon arrays. The implementation of these algorithms in aroma.affymetrix [27,28] was used.

### Outlier detection and correction

Usually there are outliers within probe intensity data. An outlier is an anomalous probe measurement that shows an abnormal distance from similar probe measurements of the same array in different experiments after the normalization step. NMF factorization (the core of the SPACE algorithm) is not robust if there are outliers in data, as it tries to accommodate them. A single factor of the factorization may be used simply to store a large outlier. Therefore, results improve by removing outliers prior to the factorization. A method to detect outliers that is closely related with the algorithm to estimate the number of transcripts was included and is explained below. Once the outliers are identified, there are different algorithms to impute their values (for a comparative review Barnett *et al*. [29]).

### Making microarray intensity data homoscedastic

NMF optimization assumes that the residuals between the initial matrix and the estimated one by the factorization are homoscedastic, i.e. that the residuals have the same variance and do not depend on probe intensity or other variables. However, the residuals tend to be larger for probes with large affinities. Therefore, probe intensity matrix is rescaled to make the residuals closer to the proposed model.

Every probe intensity was divided by the median of the values of the same probe in all experiment raised to the power of 0.7. This value was obtained, using genes with a single transcript (according to Ensembl release 51) and comparing the residuals with the median of probe intensities. Using the aforementioned transformation, the prediction results in the simulation improved significantly.

### Estimation of the number of transcripts

The basic idea of the method is borrowed from Owen *et al*. [18]. This method provides an estimation of the optimal number of components to be retained using Singular Value Decomposition (SVD) or NMF data factorizations. The optimal number of components is computed by using a resampling method that splits the expression matrix of the probes belonging to a particular gene across all samples into four submatrices. Next, each submatrix is estimated using the other three submatrices. The suggested optimal number of components is the one with a smaller average error (after performing a number of estimations) compared to the initial matrix.

The matrix of probe intensities *Y *is split into four submatrices.

(1) $$Y_{m\times n}=\left[\begin{array}{cc} P_{m_{1}\times n_{1}} & Q_{m_{1}\times n_{2}}\\ R_{m_{2}\times n_{1}} & S_{m_{2}\times n_{2}} \end{array}\right]$$

If *k *= *rank*(*Y*) is smaller than *min*(*m*_1, _*m*_2, _*n*_1, _*n*_2_), and *rank*(*Y*) = *rank*(*S*) then it can be demonstrated that the *P *submatrix can be reconstructed using the other three submatrices. Without noise, the rank of *Y *matrix is equal (or smaller in some degenerated cases) to the number of transcripts *k *which represents the dimension of NMF factorization. In this case, the *P *submatrix is identically,

(2) $$P_{m_{1}\times n_{1}}=Q_{m_{1}\times n_{2}}\cdot S_{n_{2}\times m_{2}}^{+}\cdot R_{m_{2}\times n_{1}}$$

where *m *= *m*_1 _+ *m*_2_, *n *= *n*_1 _+ *n*_2 _and *S*^+ ^is the Moore-Penrose pseudoinverse of *S*. If there is noise, *rank*(*Y*) ≠ *rank*(*S*) and the reconstruction of *P *will no longer be exact. This reconstruction is called $\tilde{P}$. In Owen *et al*. [18], it is stated that using a partial pseudoinverse and only the *k *first singular values of *S*, the expected error of the reconstruction is minimized. This decomposition of matrix *Y *can be done for any permutation *L *of the initial matrix. In this case,

(3) $${\tilde{P}}_{k}^{\left(L\right)}=Q^{\left(L\right)}\cdot S_{k}^{+(L)}\cdot R^{\left(L\right)}$$

where $S_{k}^{+}$ is the pseudoinverse of *S *using the *k *first components. This consideration provides a way to apply resampling: using different factorizations, different estimates of *P *, *Q*, *R *and *S *and therefore, the whole *Y *matrix can be obtained. These permutations can be done for different values of *k*, i.e. the number of singular vectors to reconstruct *P *using the pseudoinverse of *S*. Owen *et al*. [18] suggest to select the *k *that provides the smallest expected value of the norm of the error, i.e.,

$\min_{k}\left(E(|E_{k}^{\left(L\right)}|)\right)=E(|Y^{\left(L\right)}-{\tilde{Y}}_{k}^{\left(L\right)}|)\;.$ As *k *increases the error drops until the optimum value. For larger values of *k *the error increases only slightly. Our algorithm selects the optimal dimension by using the value of *k *(number of transcripts) whose median error is not significantly different to the number of transcripts that provides the minimum error. The corresponding statistics are obtained using a Wilcoxon test.

A small variation of this algorithm was applied to detect the outliers in the data. Each of the *L *permutations provided a estimation for each entry of *Y *matrix using the largest possible *k*. Applying these estimations a number of times (250 in our case), a density function for each of the entries of the matrix is obtained. If an entry is far from the median of its distribution, it is considered to be an outlier and its value is substituted by the median of the estimates. This method takes (~5 sec/gene).

### Summarization

After the outliers have been removed and their values estimated, NMF factorization is applied to each of the genes within the array. This step is time consuming (~6 sec/gene). This procedure was described in Anton *et al*. [17]. Briefly, this method consists of applying non-negative matrix factorization (NMF) to the matrix of probe intensities. The standard NMF applied to the matrix *Y *, yields two matrices *W *and *H*:

(4) $$Y_{m\times n}\approx W_{m\times k}\cdot H_{k\times n}$$

The first matrix *W *can be used to reveal the predicted structure of the gene (whether a particular probe belongs to a transcript or not) and the second matrix *H *the concentration of the transcripts. In Wang deconvolution model [13], the matrix equation is *Y ≈ A·F·G·T*. The feature matrix *F *and the property matrix *G *were merged into a single matrix *G *obtaining the following equation:

(5) $$Y_{m\times n}\approx A_{m\times m}\cdot G_{m\times k}\cdot T_{k\times n}$$

Comparing equation 4 and 5, it is possible to identify *W *with *A G *(the structure of the *k *transcripts) and *H *with *T *(their concentrations).

### Reshaping *W *to improve gene structure. Adapt algorithm

NMF factorization is not unique and some additional degrees of freedom exist. One matrix *D *and its inverse *D*^-1 ^can be used to transform the two matrices as follows:

(6) $$Y\approx W\cdot H=(WD)\cdot (D^{-1}H)=\tilde{W}\cdot \tilde{H}$$

and in turn, the new matrices can be identified with the *AG *matrix,

(7) $$\tilde{W}\cdot \tilde{H}=(\tilde{A}\cdot \tilde{G})\cdot \tilde{T}$$

This new factorization (given that $\tilde{W}$ and $\tilde{H}$ have all their elements non-negative) provides a different structure (and different concentrations) for each of the transcripts and reconstructs the same matrix. Since the reconstructed matrix is identical, any method to discern the "correct" *D *matrix must rely on additional properties of the data, not on the data themselves.

In our case, the non-negativity condition of $\tilde{W}$ and $\tilde{H}$ can be guaranteed if *D *is a non-negative diagonal matrix. Since the *G *matrix is binary (as proposed by Wang *et al*. [13]) and the *A *matrix is a diagonal of the affinities of the probes, all the rows in *AG *are equal to the affinity of the probe (if the probe hybridizes against the transcript) or zero (if the probe does not hybridize). There are many more probes that hybridize against transcripts than probes that do not, i.e., the *G *has many more ones than zeros. In Ensembl release 51 the median of the proportions of ones in all *G *matrices belonging to each gene is 83%. Taking these facts into account, we select the *D *matrix that provides a structure for $\tilde{W}$ that mimics the model, i.e. all the columns of $\tilde{W}$ must be similar to each other except a few probes.

The condition of having "similar" columns can be converted into an optimization problem,

(8) $$\underset{D}{\min}\left(\sum_{i=1}^{n_{p}}\sum_{j=1}^{n_{t}}\sum_{k=j+1}^{n_{t}}|\log(d_{jj}w_{ij})-\log(d_{kk}w_{ik})|\right)$$

where *n_p _*is the number of probes of a gene, *n_t _*is the number of transcripts and *d_jj _*are the elements of the diagonal of matrix *D*. This optimization problem can be converted into a system of linear equations that can be solved using robust statistical methods, i.e. the presence of outliers do not significantly affect the solution. Once *d_jj _*and hence $\tilde{W}$ are computed, the affinity of the probes are the maximum values of each row of this matrix.

(9) $${\tilde{a}}_{ii}=\underset{k}{\max}({\tilde{W}}_{ik})$$

and *G *can be computed by,

(10) $$\tilde{G}={\tilde{A}}^{-1}\cdot \tilde{W}$$

In this equation, all the entries of the $\tilde{G}$ matrix are between zero and one, since they are obtained by dividing each entry of $\tilde{W}$ by the maximum value for each of the rows.

### Using the information of the location of the probes. Structure coherence correction

Affymetrix probes are grouped in sets of probes. A set of probes should hybridize against the same feature of a gene (an exon or a part of an exon) and can be considered as a whole. The previous steps of the algorithm do not take this fact into account and treat each probe independently. The predicted structure can contain errors due to bad quality probes, large noise or limitations of the algorithm. We can take advantage of Affymetrix redundancy by forcing the coherence of the set of probes; all of them have the same pattern against the different transcripts. In the suggested matrix form, as the hybridization pattern of a probe corresponds to a row of the *G *matrix, all the rows that correspond to probes within the same set must be identical.

Our algorithm selects for each probeset (a subset of rows of matrix *G*) the closest binary matrix of rank equal to one (all the rows have the same values). This matrix is obtained simply by rounding the mean value of each of the columns of the submatrix that corresponds to the probeset. Finally, a threshold is set to all the entries of the *G *matrix.

## Authors' contributions

AR and MA conceived the idea and wrote the manuscript. AR, MA and AA developed the software. AA wrote the vignette to explain the usage of the algorithm. All authors read and approved the final manuscript

## Supplementary Material

Additional file 1 **Prediction of the structure of genes from several datasets using SPACE algorithm**. SPACE performance taking Ensembl release 51 annotated genes as reference (Affymetrix sample dataset of human tissues). Improvement in the estimation of transcript concentrations after applying Adapt algorithm (synthetic data). Comparison between SPACE algorithm and Wang deconvolution model to estimate the concentrations of transcripts (synthetic data). Structure prediction and concentrations estimation of WNK1 gene using SPACE algorithm compared to Wang deconvolution model results (Affymetrix sample dataset of human tissues). Predicted structure and concentrations estimation of several genes using public datasets. These genes have RT-PCR validated splicing events in several studies: de la Grange *et al*. [8], French *et al*. [20], Xing *et al*. [22] and Hung *et al*. [23]. Click here for file

Additional file 2 **Table S1**. Table in Excel format that summarizes the results of the predictions for each of the datasets. Click here for file

Additional file 3 **R package of SPACE algorithm for Affymetrix exon arrays**. SPACE Binary R package to be used in Windows Platforms. Click here for file

Additional file 4 **R script**. Piece of code to run the proposed algorithms in a small set of data. This file includes comments to set the proper directory structure and files. Click here for file

Additional file 5 **R package of SPACE algorithm. Source code**. Source code of the R package. Click here for file

Additional file 6 **Manual of the SPACE R package**. Manual of the SPACE R package. Click here for file
