# Supplementary material for: Improvements to previous algorithms to predict gene structure and isoform concentrations using Affymetrix Exon arrays
Source: BMC Bioinformatics. 2010 Nov 26;11:578. doi: 10.1186/1471-2105-11-578 (PMC3012675; doi:10.1186/1471-2105-11-578)
Supplement: Additional file 1 — Prediction of the structure of genes from several datasets using SPACE algorithm. SPACE performance taking Ensembl release 51 annotated genes as reference (Affymetrix sample dataset of human tissues). Improvement in the estimation of transcript concentrations after applying Adapt algorithm (synthetic data). Comparison between SPACE algorithm and Wang deconvolution model to estimate the concentrations of transcripts (synthetic data). Structure prediction and concentrations estimation of WNK1 gene using SPACE algorithm compared to Wang deconvolution model results (Affymetrix sample dataset of human tissues). Predicted structure and concentrations estimation of several genes using public datasets. These genes have RT-PCR validated splicing events in several studies: de la Grange et al. [8], French et al. [20], Xing et al. [22] and Hung et al. [23]. [file 1471-2105-11-578-S1.PDF]

Improvements to previous algorithms to predict gene structures and isoform concentrations using Affymetrix Exon arrays

Miguel A Anton[1], Ander Aramburu[1]and Angel Rubio*[1]

[1]CEIT and TECNUN, University of Navarra, San Sebastián, Spain

Email: Miguel A Anton - maanton@ceit.es; Ander Aramburu - aaramburu@ceit.es; Angel Rubio*- arubio@ceit.es;

*Corresponding author

SPACE algorithm performance taking Ensembl annotation of genes as reference

A whole genome validation of SPACE algorithm using the Affymetrix sample dataset of 11 human tissues was performed. The validation was done taking Ensembl release 51 annotated transcripts as reference.
The G matrices of SPACE were built using the structure of 1600 randomly selected genes. To do this, we used the CDF files provided by Brainarray version 11 for genes and transcripts. Additionally, using the CDF that groups the probes by exons, the sets of probes that correspond to each "indivisible" sections of the gene were obtained. These "indivisible" sections have the same purpose as Affymetrix probesets. For example, if a gene has AS in alternative donor or acceptor sites, the indivisible parts correspond to each part of the exon that are constitutive or alternatively spliced. All the probes within an indivisible part must have the same hybridization matrix.
The G matrices were considered to be the "ground truth" to test our algorithm. However, if a transcript is not present in any of the samples, its structure is impossible to predict. Ensembl annotation pipeline includes some transcripts derived from ESTs, that are present only in tumoral tissues. Therefore, those transcripts will not be predicted in the Affymetrix dataset of tissues. Additionally, a suitable selection of quality probes is very important. In many cases, it is almost impossible to predict this matrix using only the expression data (as with SPACE algorithm). There are probe intensities of the same gene that along samples do not show correlation with any other probe intensity (the correlation is not larger than with other probes of the array).

The ROC curves of our predictions were made assuming that the G matrices are accurate. They are shown in figure 1. Three ROC curves appear in the figure: SPACE predictions without improvements; SPACE prediction after improvements; and SPACE applied to example-based synthetic data. Additionally, the comparison between the estimated number of transcripts by SPACE and the number of transcripts in Ensembl is shown in figure 2.

The example-based synthetic data of the third ROC curve was constructed as follows. Using the Wang *et al.* deconvolution algorithm ($Y \approx A \cdot G \cdot T$), the affinities ($A$ matrix) and transcript concentrations (T matrix) were estimated using the intensity matrix (Y matrix) and the property G matrix. With these estimations, the residues (Res) of the model (assuming that they are multiplicative noise) were computed.

$$Y = (AGT)(1 + Res)$$

These residues were shuffled ($\widetilde{Res}$) and a new intensity matrix ($\widetilde{Y}$) was built. This $\widetilde{Y}$ matrix is the example-based synthetic data to which SPACE was applied.

$$\widetilde{Y} = (AGT)(1 + \widetilde{Res})$$

If the gene structure in Ensembl is accurate, $\widetilde{Y}$ matrix will be very close to Y matrix. Moreover, shuffling the residues of the genes with incorrect annotation has the effect of converting them into random outliers. As the ROC curve using this synthetic data is better than using real data, there is a structure in the residues. This is due to errors in the gene structure annotated in Ensembl, cross-hybridizations of the probes or the limitations of the model. The ROC curve using the synthetic data can be considered as a "best possible case" for a particular dataset (no annotation errors, no cross-hybridization and perfect linear behavior of all the probes in a gene).

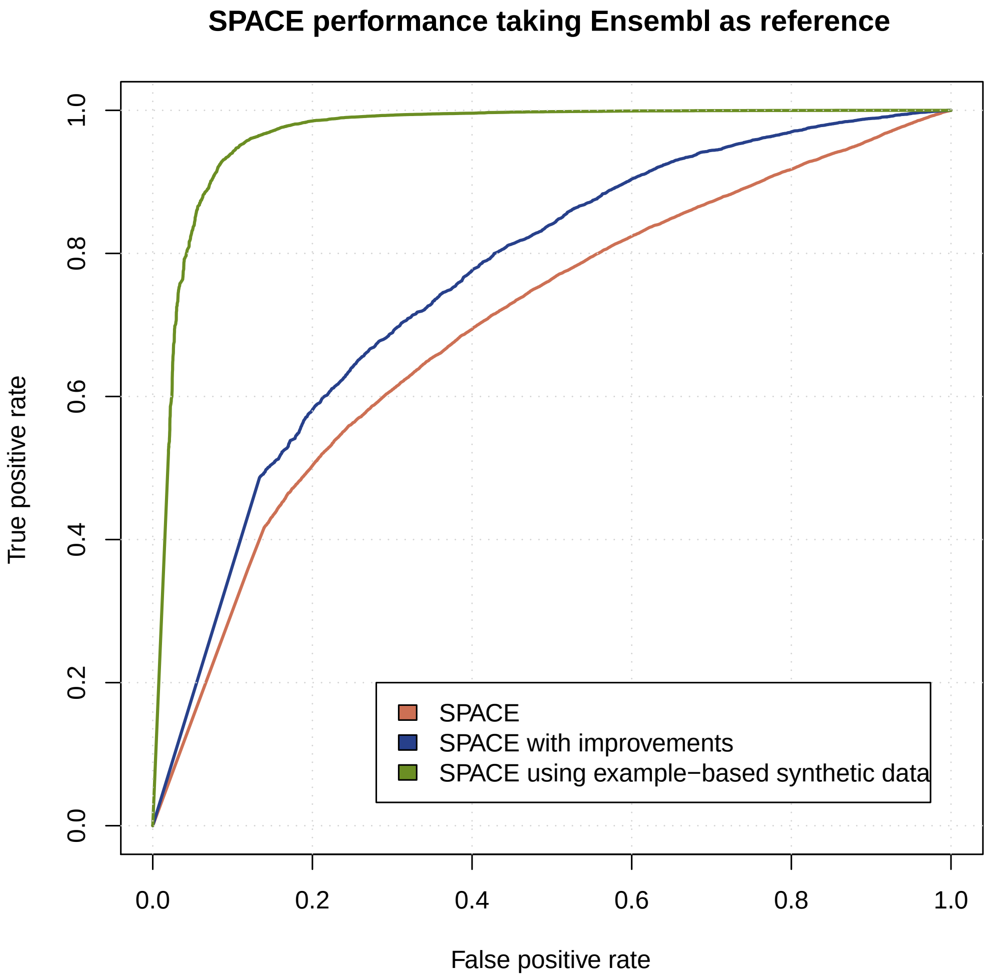

Figure 1: ROC curves of splicing structure predictions. The three ROC curves of this figure measure the concordance of the predictions with the transcript structure that appear in Ensembl release 51. The y-axis shows the sensitivity (the proportion of probes that are said to hybridize against a transcript and actually do) and in the x-axis 1 - specificity is shown (the proportion of probes that are said not to hybridize against a transcript and actually do not). The red ROC curve corresponds to the prediction without taking into account the improvements described in this paper. The blue ROC curve is obtained after applying Adapt algorithm and structure coherence correction (probes within the same exon or exon part must have the same hybridization pattern). Performance improves using the new version of the algorithm. The green ROC curve corresponds to the prediction using example-based synthetic data. This synthetic data is made using the predicted transcript concentrations and estimated probe affinities with shuffled noise. It can be considered as the "best possible case" of the algorithm for a particular set of data.

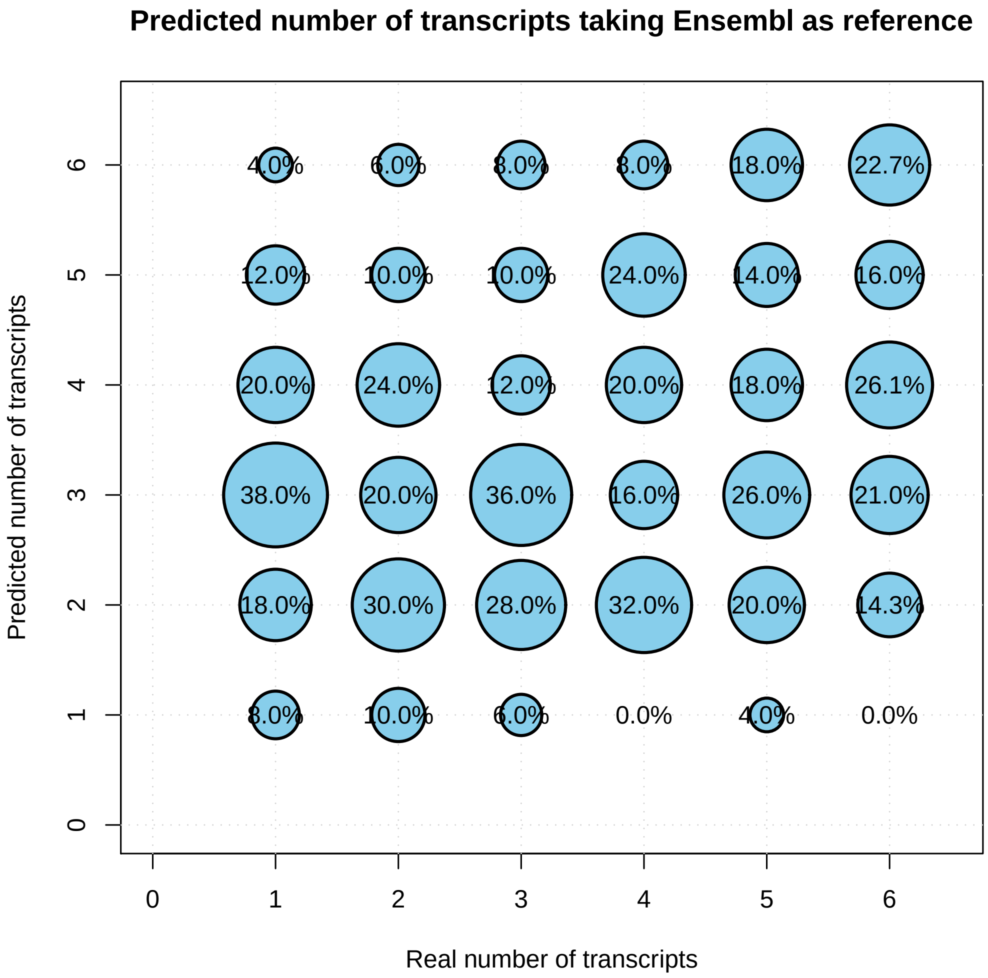

Figure 2: Estimation of the number of transcripts for each gene compared to the number of transcripts that appear in Ensembl release 51. It can be seen that if the number of transcripts in Ensembl increases, the number of predicted transcripts tends usually to be larger. However, this trend is only a subtle trend. Some reasons to explain the differences between the number of predicted isoforms and the number of isoforms in Ensembl are limitations of the algorithm, incorrect number of isoforms in the Ensembl databse, incorrect number of isoforms for this particular dataset, errors in modeling of the probes -cross hybridization for example.

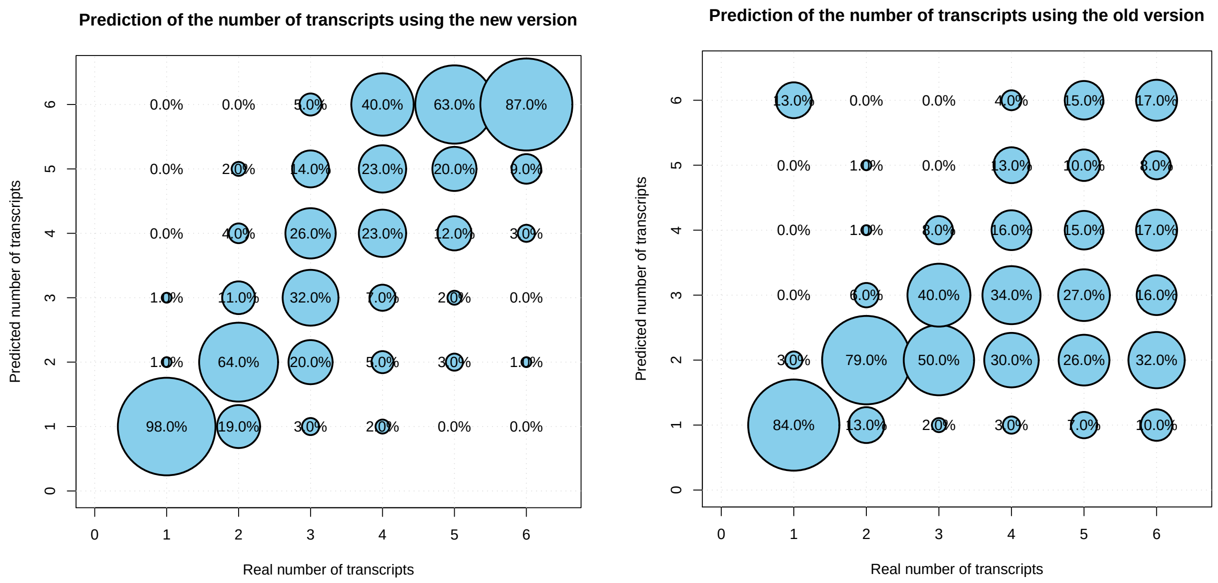

Figure 3: Comparison of the estimation of the number of transcripts using the new version of the algorithm (left panel) and the previous one (right panel) with synthetic data. These synthetic data have much less noise compared to those shown in the main manuscript (roughly the standard deviation is ten times smaller). If the same level of noise was used, the previous algorithm would perform so badly that it would be difficult to make any comparison. The previous version performs well if the number of transcripts is small (one or two). However, for larger number of transcripts, the predicted number using the previous algorithm is underestimated. We have computed the relative error between the true number of transcripts and the real number of transcripts, i.e., $mean(\frac{|N_{Predicted} - N_{Real}|}{N_{Real}})$. This value is 62% for the previous algorithm and 18.2% for the new algorithm. The absolute error $mean(|N_{Predicted} - N_{Real}|)$ also improves with the new algorithm: 1.23 (old) vs 0.36 (new). We also tested that the difference in the relative error was statistically significant (paired Wilcoxon test p.value = 5.7E-10). A similar result occurs for absolute error.

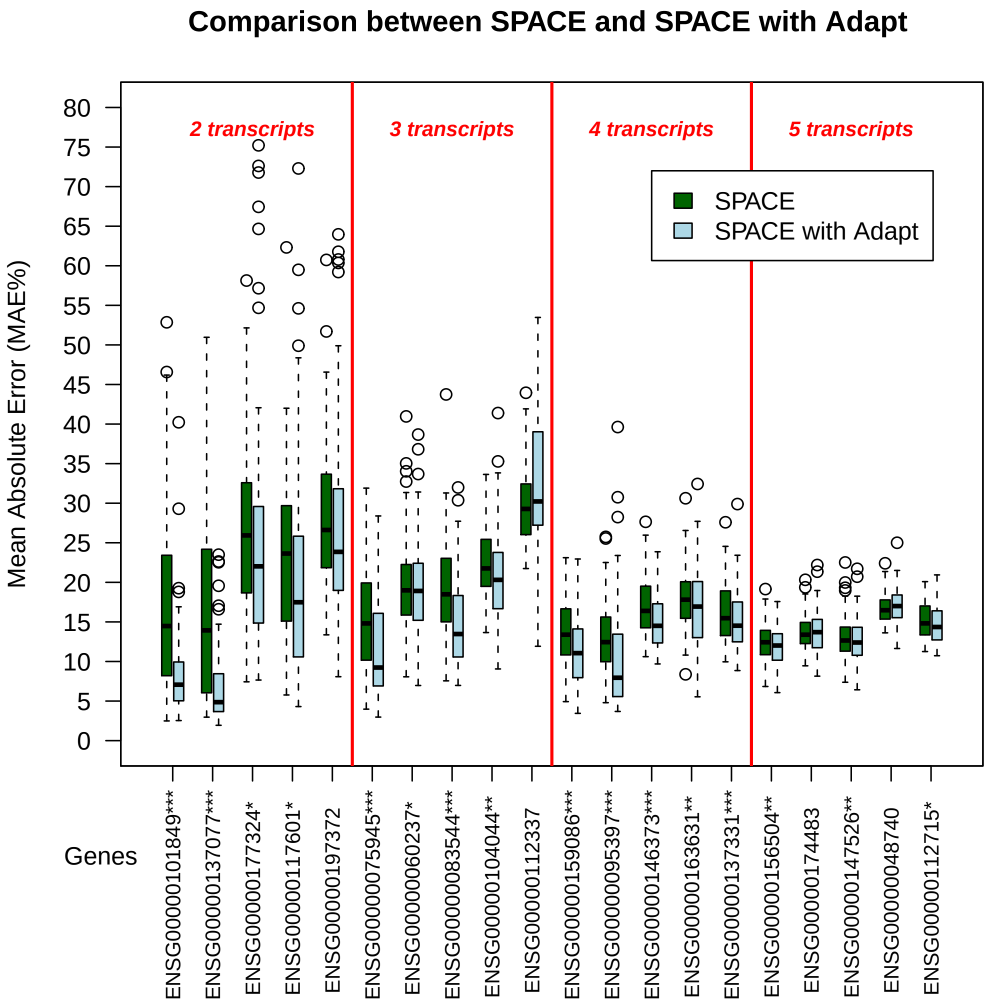

Figure 4: Mean Average Error (MAE%) between the relative concentrations of alternatively spliced isoforms and the relative concentrations estimated by SPACE for genes with 2 to 5 transcripts (synthetic data). The mean average error (MAE%) is defined as the mean of the unsigned differences between the actual relative concentrations and the estimated ones by the algorithm. Green boxplots correspond to the error of the estimated concentrations using SPACE without Adapt algorithm and blue boxplots to the error after applying Adapt. It can be observed that Adapt algorithm improves the estimation of transcript concentrations. This improvement is more apparent when the number of transcripts is low (2 to 4 transcripts). A paired Wilcoxon test was performed for each of the genes. After the name of each of the genes, appears three asterisks (p.value $<$ 1e-5), two asterisks (p.value $<$ 0.01), one asterisk (p.value $<$ 0.05) or no asterisk (p.value non significant).

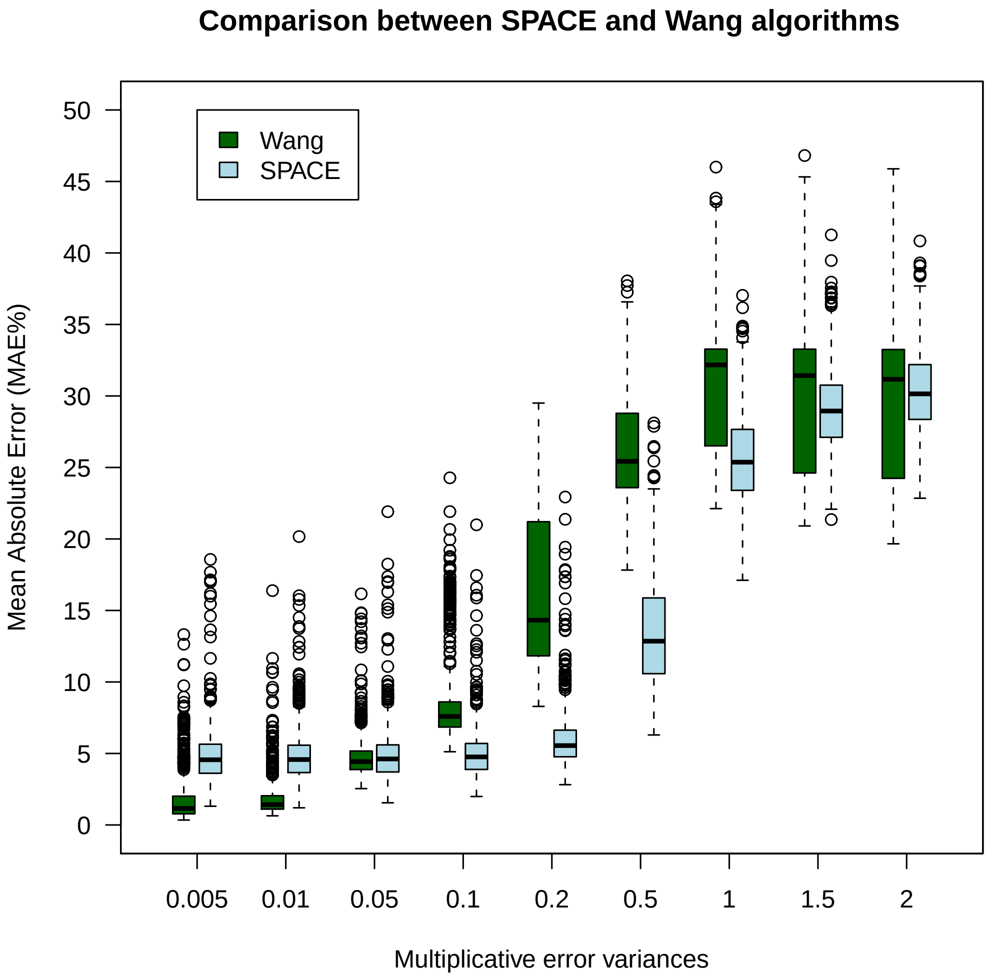

Figure 5: Mean Average Error (MAE%) between the relative concentrations of alternatively spliced isoforms estimated by the Wang deconvolution model (green plotboxes) and the relative concentrations estimated by SPACE (blue plotboxes) for a gene (ENSG00000010671) with seven transcripts under different degrees of multiplicative noise (synthetic data). In the simulated transcript concentrations data, only three of the transcripts are expressed. The other ones are not expressed at all. Multiplicative noise is in the form of $y \cdot e^{\eta}$ with $\eta \sim N(0, {\sigma_{\eta}}^2)$. The units of the x-axis represent the variances ${\sigma_{\eta}}^2$ of the multiplicative error (0.005, 0.01, 0.05, 0.1, 0.2, 0.5 ,1 ,1.5, 2). We noticed that, when estimating the concentrations of different transcripts using the Wang algorithm, many of them are predicted not to be expressed in any sample (in the Affymetrix dataset of tissues). This result is reasonable since many ESTs (used to build the Ensembl database) have been obtained using data from pathological samples. For these cases, in which only a few transcripts are expressed, Wang model includes "too many" degrees of freedom and SPACE (using the internal dimension of the predicted number of transcripts) outperforms the Wang model for intermediate levels of noise. The level of noise for the Affymetrix tissue dataset has a multiplicative variance around 0.05.

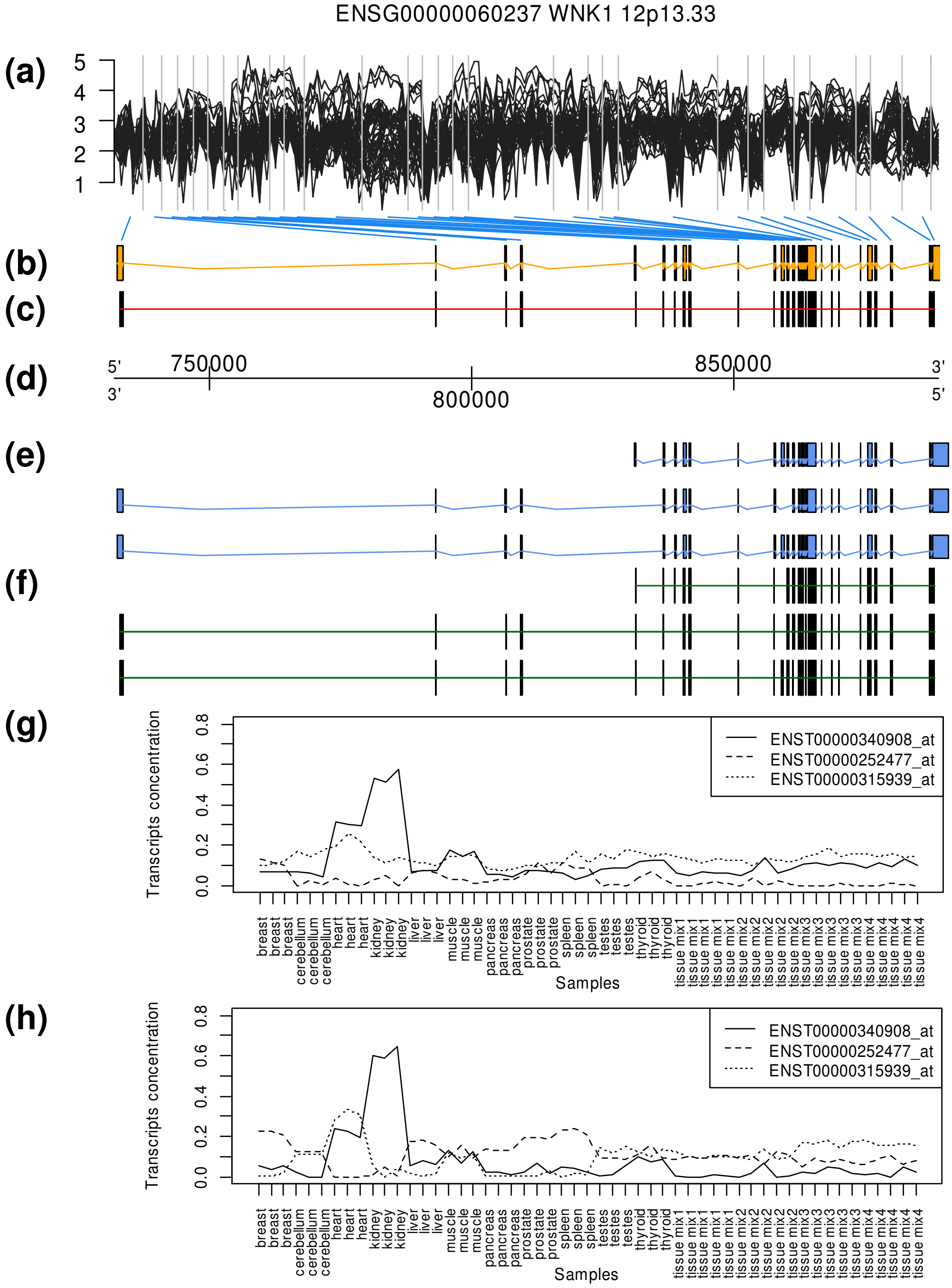

Figure 6: Comparison of the Results for WNK1 gene using both Wang and SPACE algorithms (Affymetrix sample dataset of human tissues). This gene has been used to test other algorithms in previous studies (Xu *et al.*, nar 2002). There are 185 probes in the Affymetrix exon array that map to this gene. (a) Log of the intensities for all the probes within the gene. Each line corresponds to a different sample. (b) Structure of the WNK1 gene, i.e. the exons that appear in any of its transcripts. (c) Locations of the exon probes. It can be observed that each exon has one or several probes mapped to them. (d) Genomic positions of the probes. (e) Structure of the different transcripts of WNK1 gene in Ensembl release 51. Three different transcripts are represented. (f) Predicted structure using SPACE for three transcripts. The alternative start site of the first transcript is correctly predicted. The exon cassette of the second transcripts is also predicted (the predicted cassette is shorter in the prediction than in Ensembl). (g) Predicted concentrations for each of the three transcripts using the Wang algorithm. It can be seen that the first transcript (a kidney specific isoform) is much more expressed in kidney than in any other tissue. (h) Predicted concentrations using SPACE. It shows even more clearly that the first isoform is kidney specific.

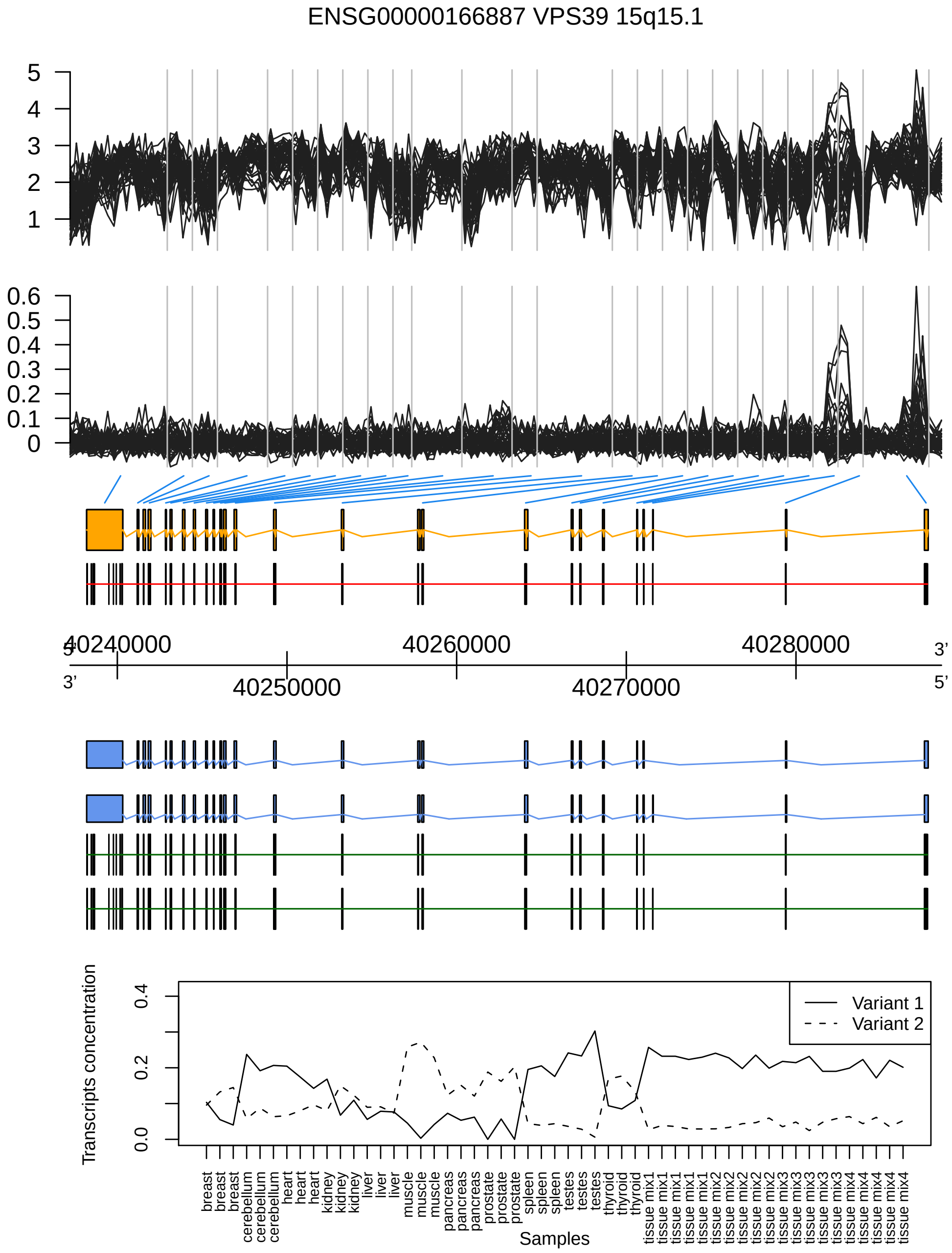

Figure 7: Results for VPS39 gene (Affymetrix sample dataset for human tissues, reverse strand). In the RT-PCR analysis of de la Grange *et al.*, (nar 2010), two isoforms were identified with mutually exclusive exons 2 and 3. On the contrary, Ensembl shows a cassette of exon three in isoform 1. Exon 2 is not exclusive with exon 3. In this case, SPACE and Ensembl structures are identical and miss the cassette of exon 2 validated by PCR. In our results, isoform variant 1 is predominant in cerebellum, heart, liver, spleen and testes. Additionally, variant 2 is more abundant in breast and muscle. These results are in concordance with RT-PCR (except cassette 2) and Ensembl release 51 annotation.

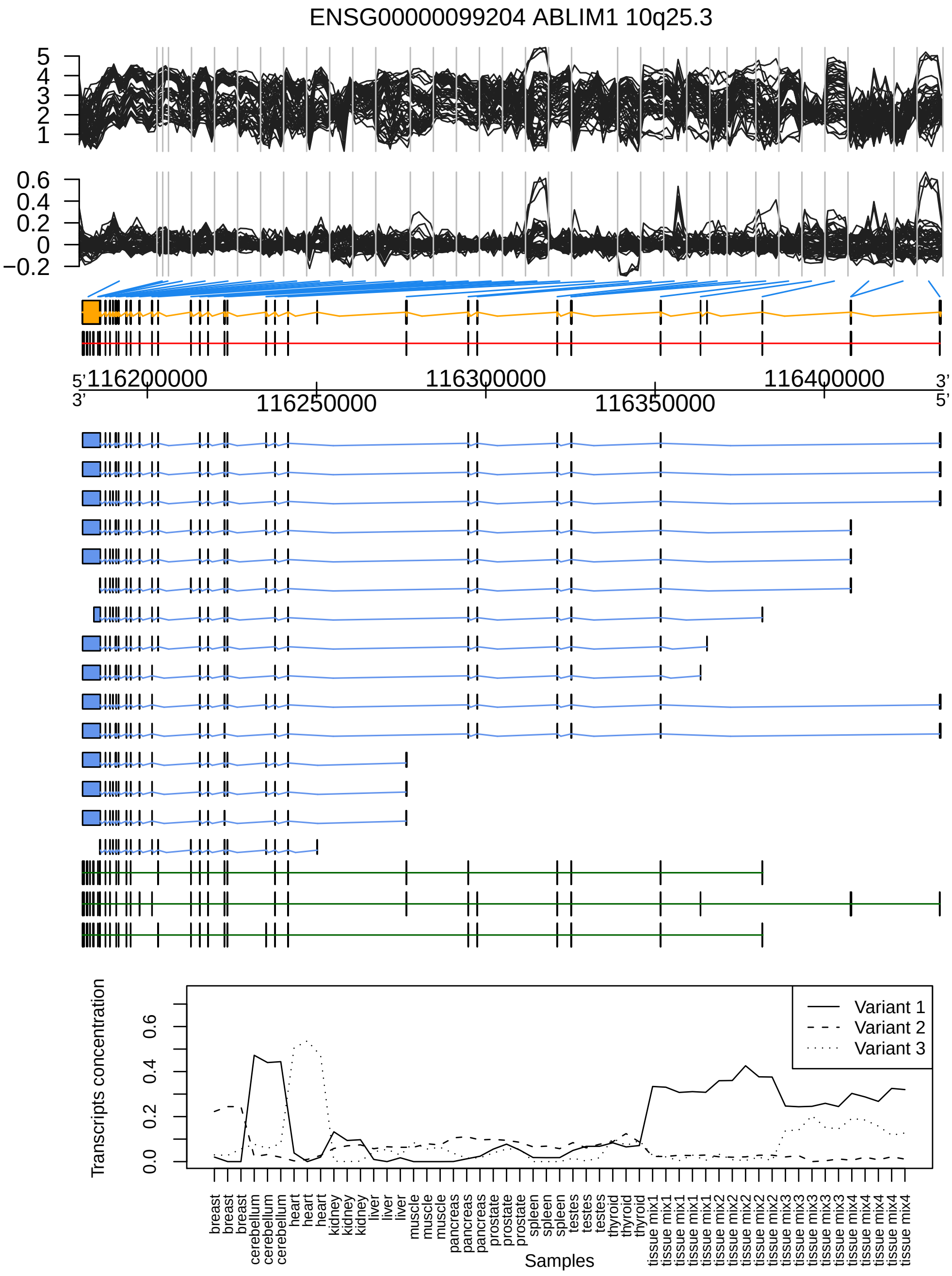

Figure 8: Results for ABLIM1 gene (Affymetrix sample dataset for human tissues, reverse strand). In the RT-PCR analysis of de la Grange *et al.*, (nar 2010), there were two cassette exons 13 and 10 and a short isoform that started in exon 10. In our results, three variants were identified. Isoform variant 3 is predominant in heart and abundant in muscle and behaves as the isoforms that has exon 13 in the RT-PCR. Isoform variant 1 is predominant in cerebellum and is abundant in testes and behaves as the isoform that starts in exon 10 in the RT-PCR. Isoform variant 2 is more abundant in the other tissues.

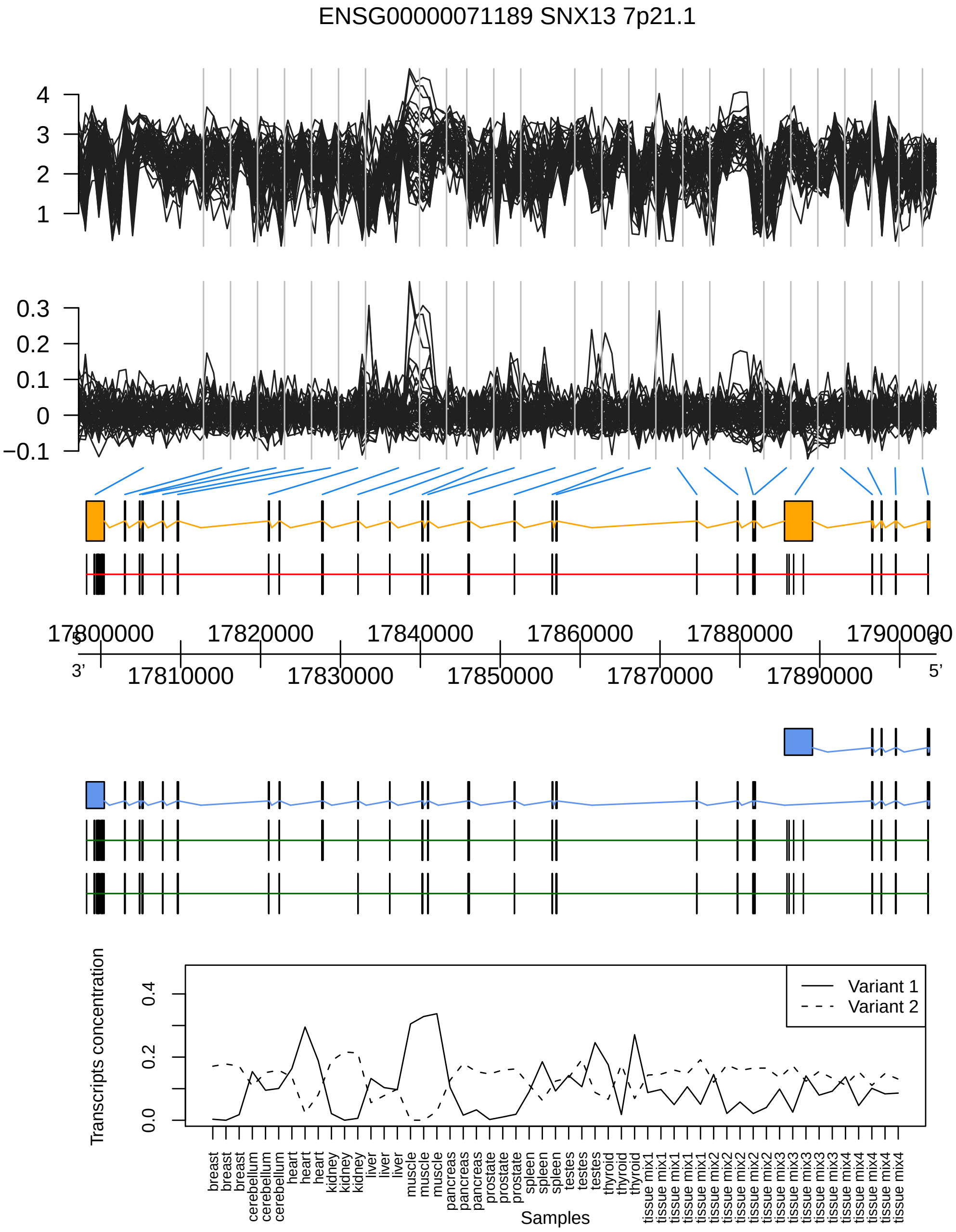

Figure 9: Results for SNX13 gene (Affymetrix sample dataset for human tissues, reverse strand). In the RT-PCR analysis of de la Grange *et al.*, (nar 2010), two isoforms were described, one variant with a long exon 21 and another variant with a short exon 21. In our results, two variants were identified, variant 1 is predominant in heart and muscle and variant 2, which has a cassette, is more abundant in breast, cerebellum and testes. These results are in concordance with RT-PCR althoug the actual predicted concentrations differ.

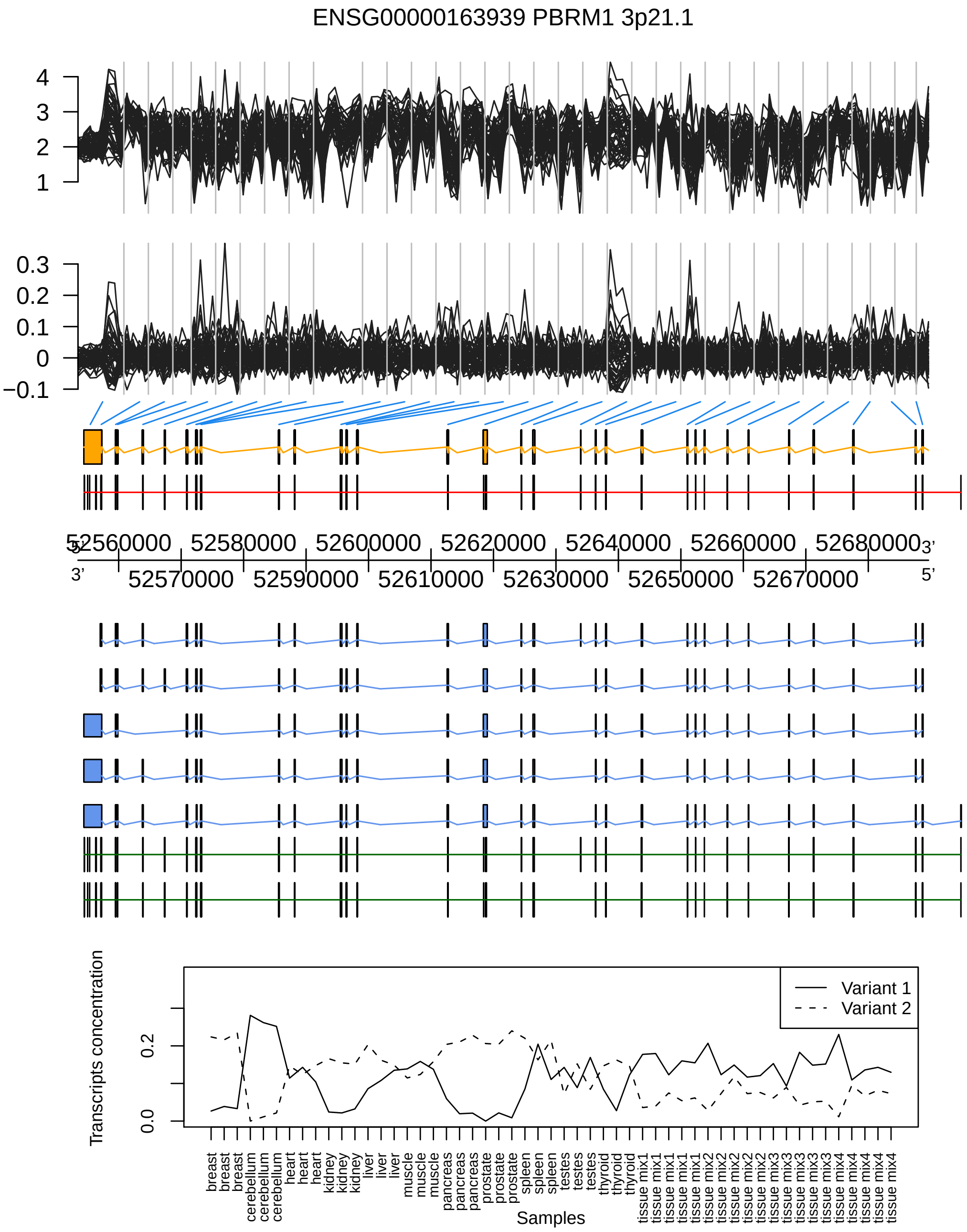

Figure 10: Results for PBRM1 gene (Affymetrix sample dataset for human tissues, reverse strand). In the RT-PCR analysis of de la Grange *et al.*, (nar 2010), a cassette was described in exon 15. Our results also show two isoforms and a cassette in exon 15. Isoform variant 1 includes that exon and is more abundant in cerebellum, heart and muscle in concordance with RT-PCR results.

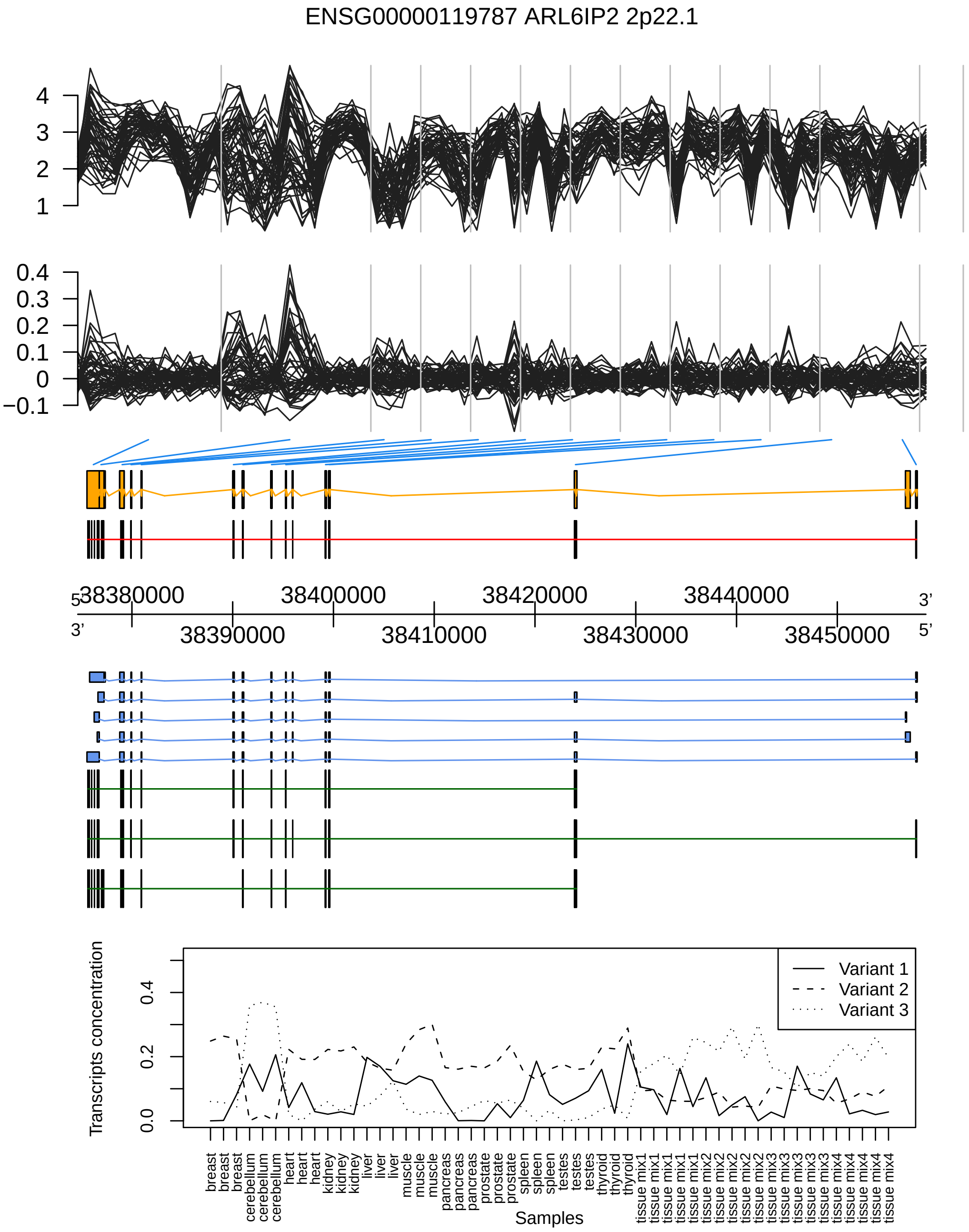

Figure 11: Results for ARL6IP2 gene (Affymetrix sample dataset for human tissues, reverse strand). In the RT-PCR analysis of de la Grange *et al.*, (nar 2010), two isoforms were described that differed in the length of final exon 13. Our results show three isoforms, variant 1 and 2 have a short final exon and variant 3 a long final exon. Isoform variant 3 is predominant in cerebellum and the other two variants in the remaining tissues in concordance with RT-PCR results.

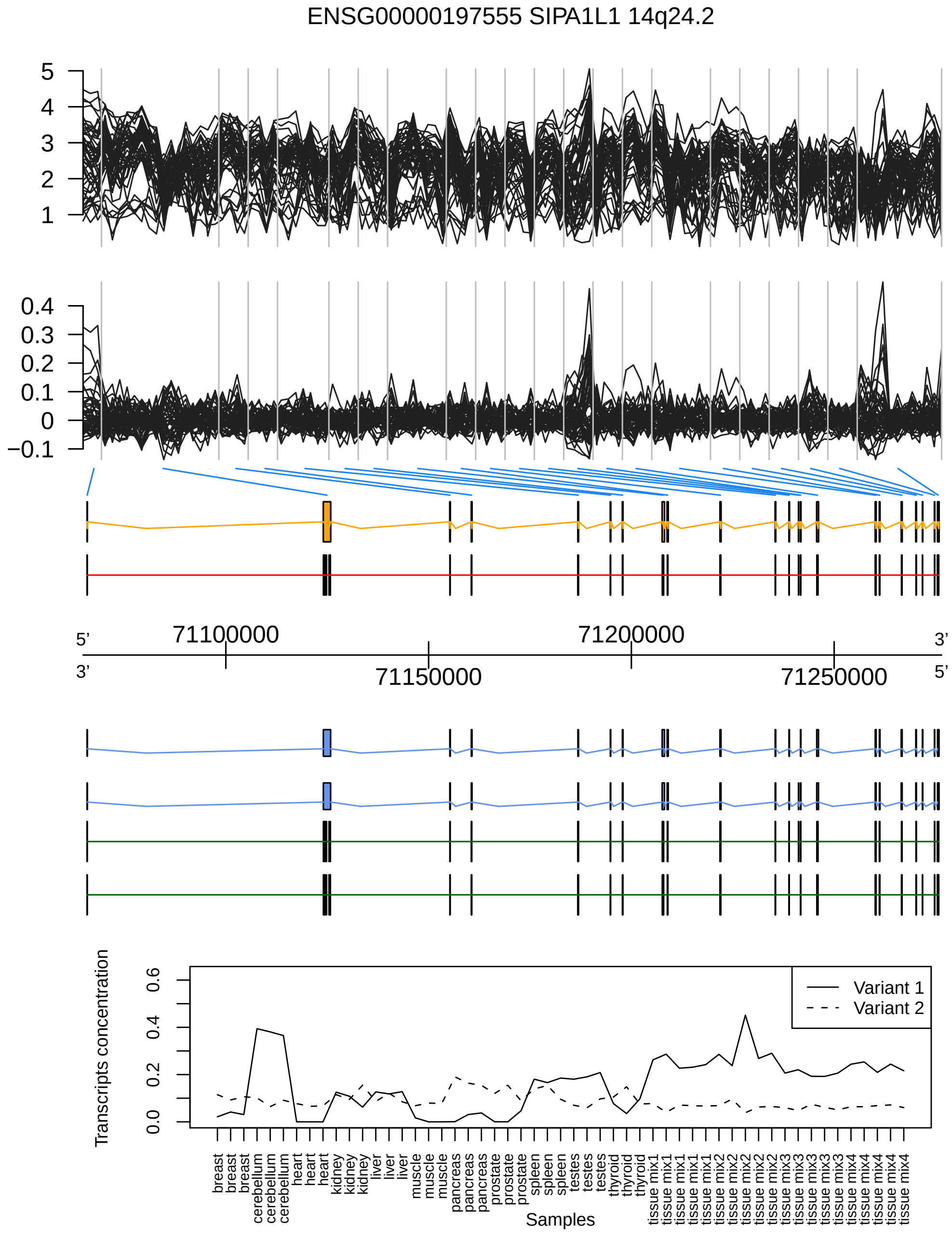

Figure 12: Results for SIPA1L1 gene (Affymetrix sample dataset for human tissues, direct strand). In the RT-PCR analysis of de la Grange *et al.*, (nar 2010), a cassette was described in exon 14. Our results also show two isoforms and a cassette in exon 14. Isoform variant 2 that lacks exon 14 is present in all tissues in concordance with the RT-PCR. Isoform variant 1 that includes exon 14 is predominant in cerebellum but not in heart and muscle and in this aspect differs from the RT-PCR.

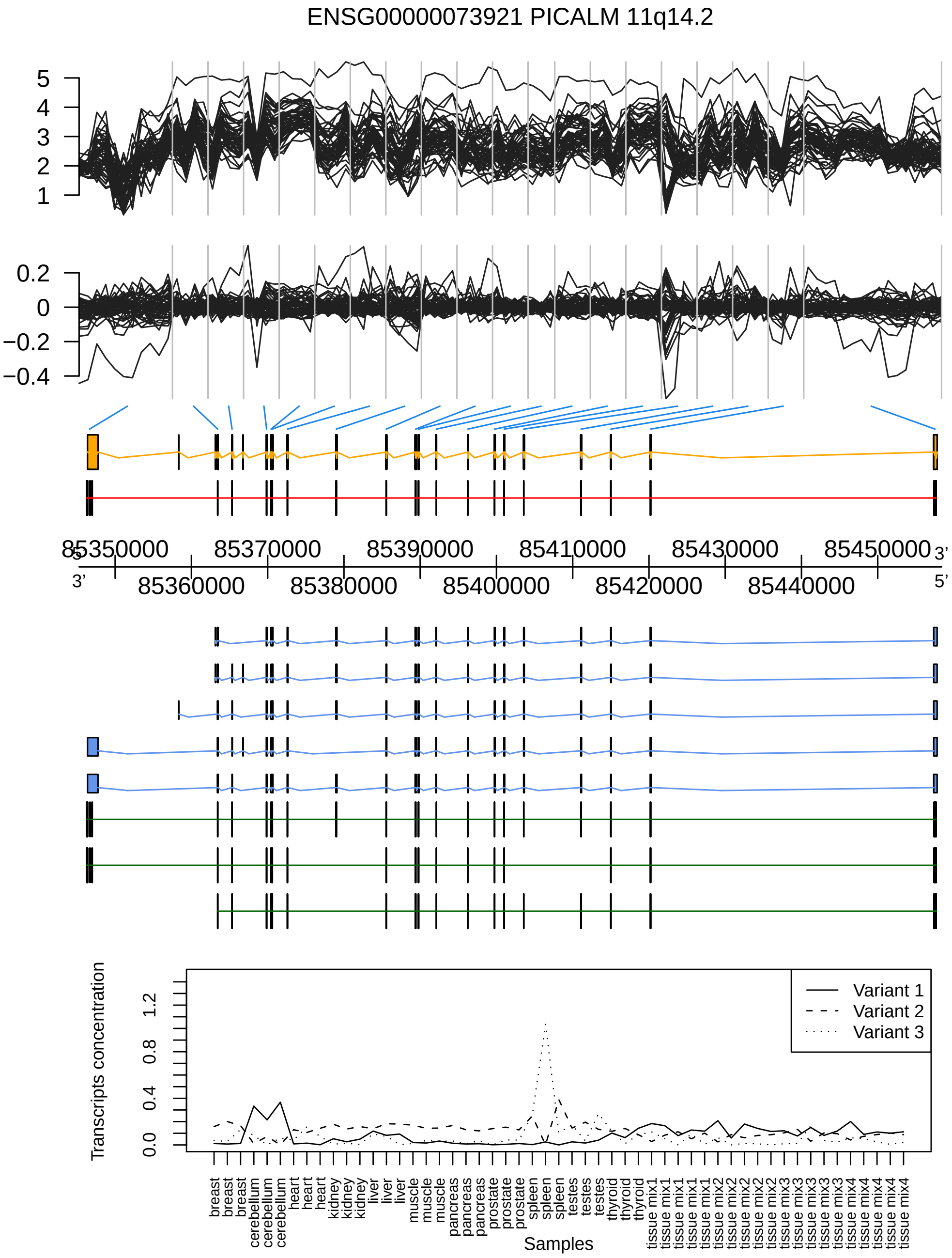

Figure 13: Results for PICALM gene (Affymetrix sample dataset for human tissues, reverse strand). In the RT-PCR analysis of de la Grange *et al.*, (nar 2010), three isoforms were described, one lacked exon 13 and the other two variants differed in the length of exon 13. Our results show three isoforms, variant 1 includes exon 13 and the other two variants lack that exon. Variant 1, which includes exon 13, is predominant in cerebellum and the other two variants are more abundant in the other tissues in concordance with RT-PCR

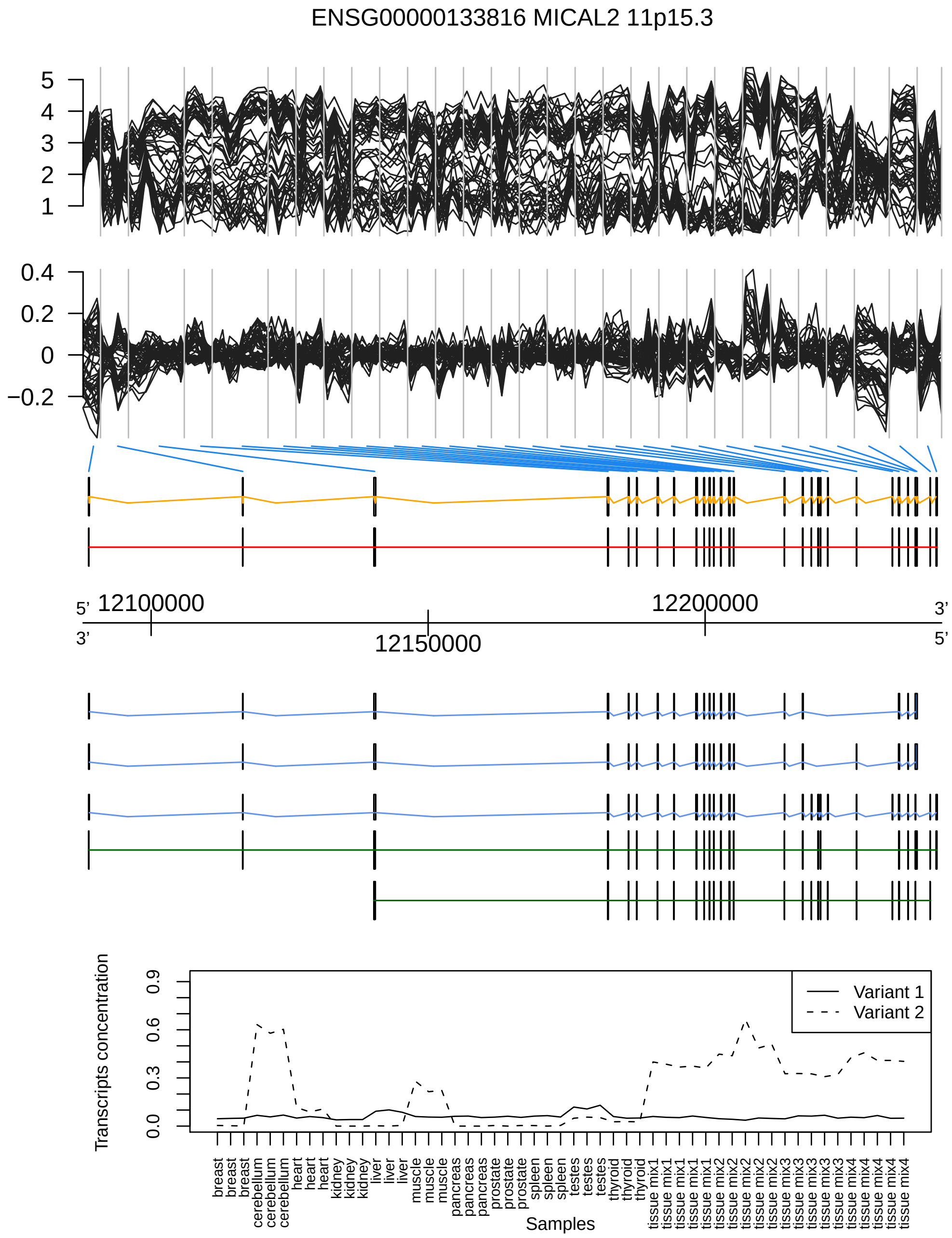

Figure 14: Results for MICAL2 gene (Affymetrix sample dataset for human tissues, direct strand). In the RT-PCR analysis of de la Grange *et al.*, (nar 2010), one cassette in exons 19 and 20 is described in one figure and exons 26 and 27 were differentially used between isoforms in the end part of the gene in other figure. Our results show two different isoforms that differ in the end of the gene, it also can be observed that around the cassette described in the RT-PCR two exons are missing but are exons 18 and 21 and not 19 and 20. Isoform variant 1 that includes more exons at the end of the gene is predominant in cerebellum, heart, muscle and abundant in testes in concordance with RT-PCR.

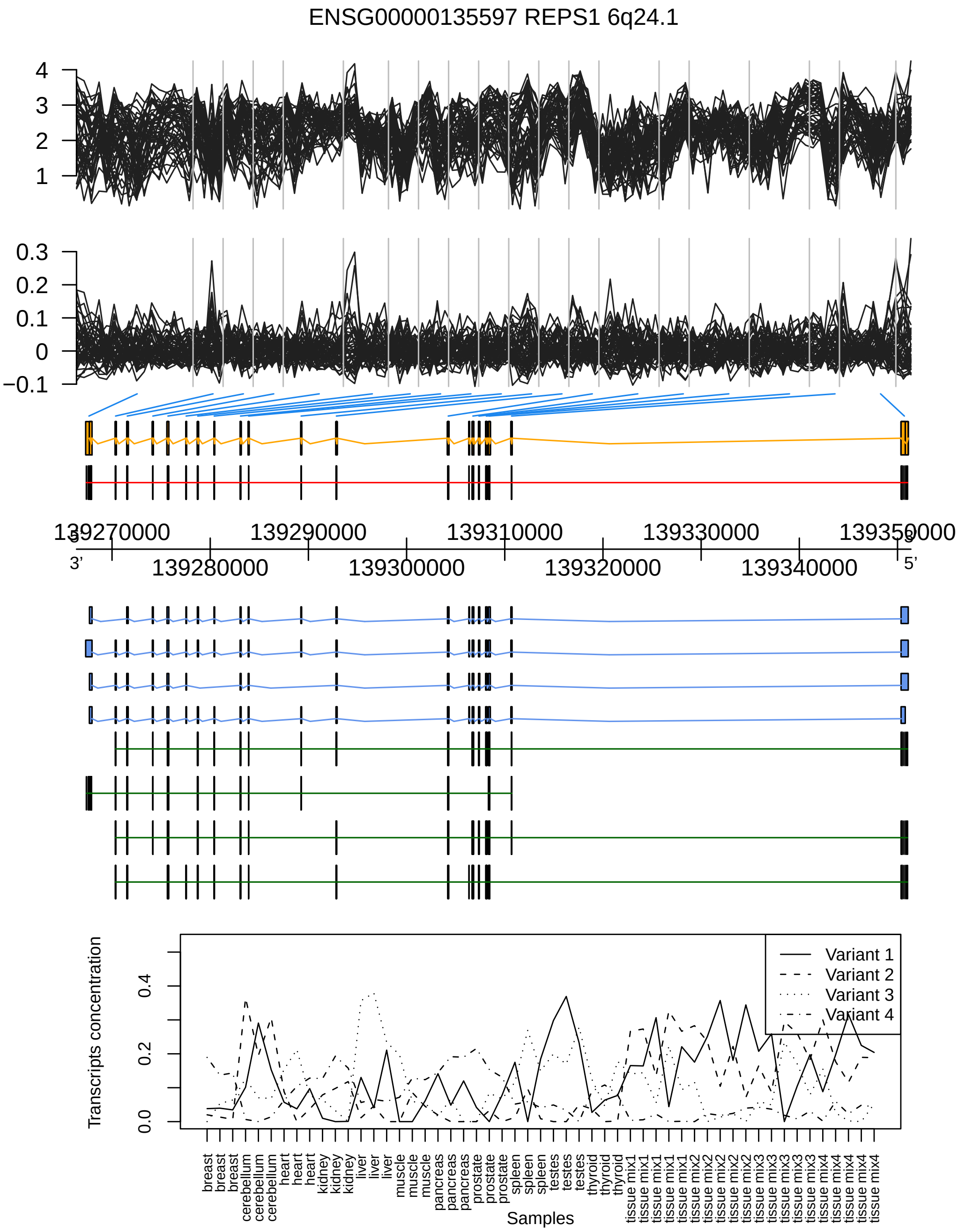

Figure 15: Results for REPS1 gene (Affymetrix sample dataset for human tissues, reverse strand). In the RT-PCR analysis of de la Grange *et al.*, (nar 2010), three isoforms were described, one isoforms lacked exon 10, the second isoform included exon 10, and the last isoform included both exon 10 and intron between exons 9 and 10. Our results comprise four variants, fist and second variants include exon 10 and third and fourth exclude that exon. Isoform variant 1 is abundant in cerebellum and testes and in some samples of heart, liver and spleen. The sum of isoforms variant 3 and 4, which lack exon 10, is abundant in all tissues. These results are in concordance with RT-PCR.

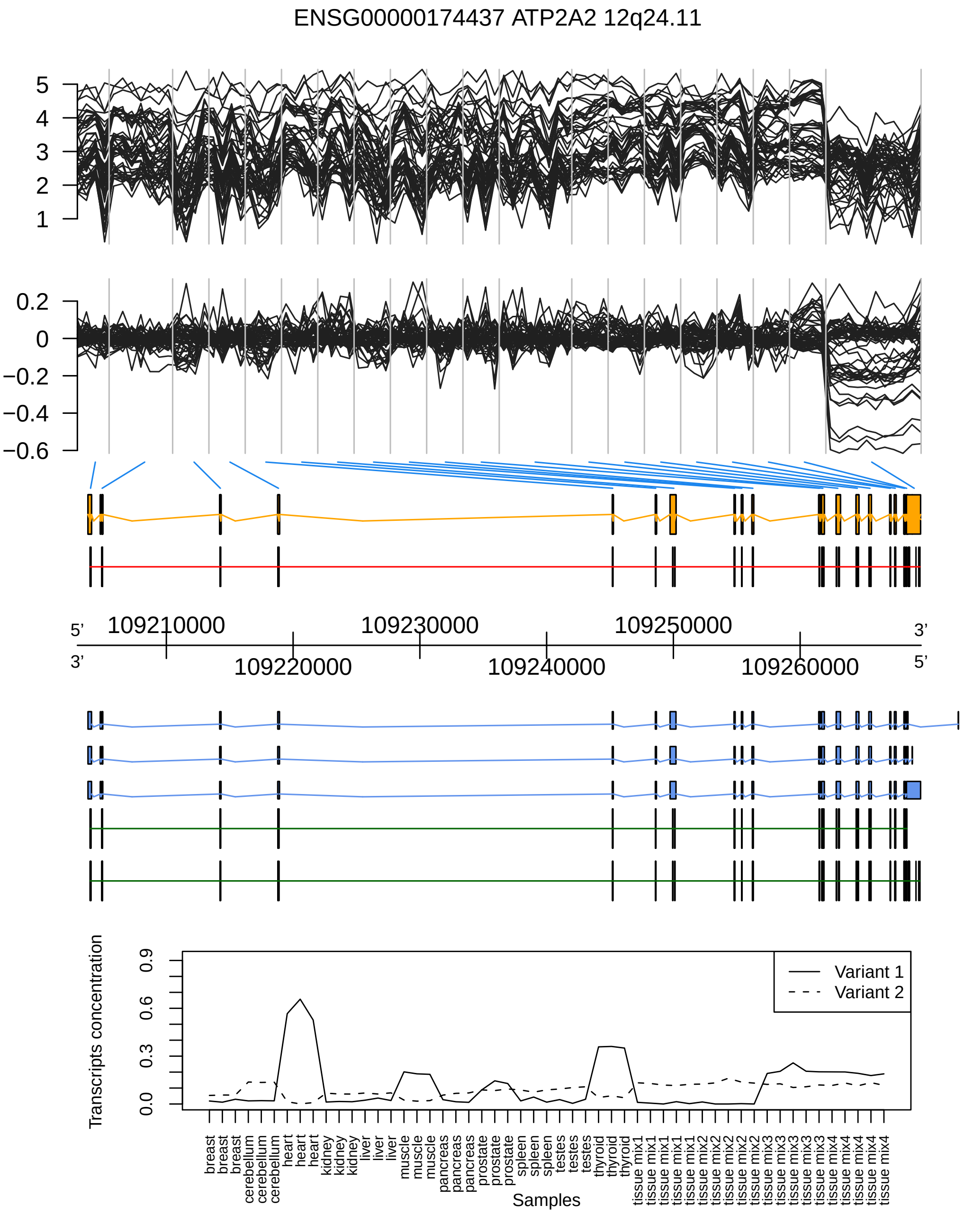

Figure 16: Results for ATP2A2 gene (Affymetrix sample dataset for human tissues, direct strand). In the RT-PCR analysis of de la Grange *et al.*, (nar 2010), two isoforms were described that differed in the final exon, one isoform ended in exon 21 with exon 20 skipped and the other isoform ended in exon 20. Our results matches RT-PCR except for exon 21 that is not mapped in brainarray transcript CDF. Isoform variant 1 skips exon 20 and isoform variant 2 includes it. Variant 1 is predominant in heart and muscle in concordance with RT-PCR.

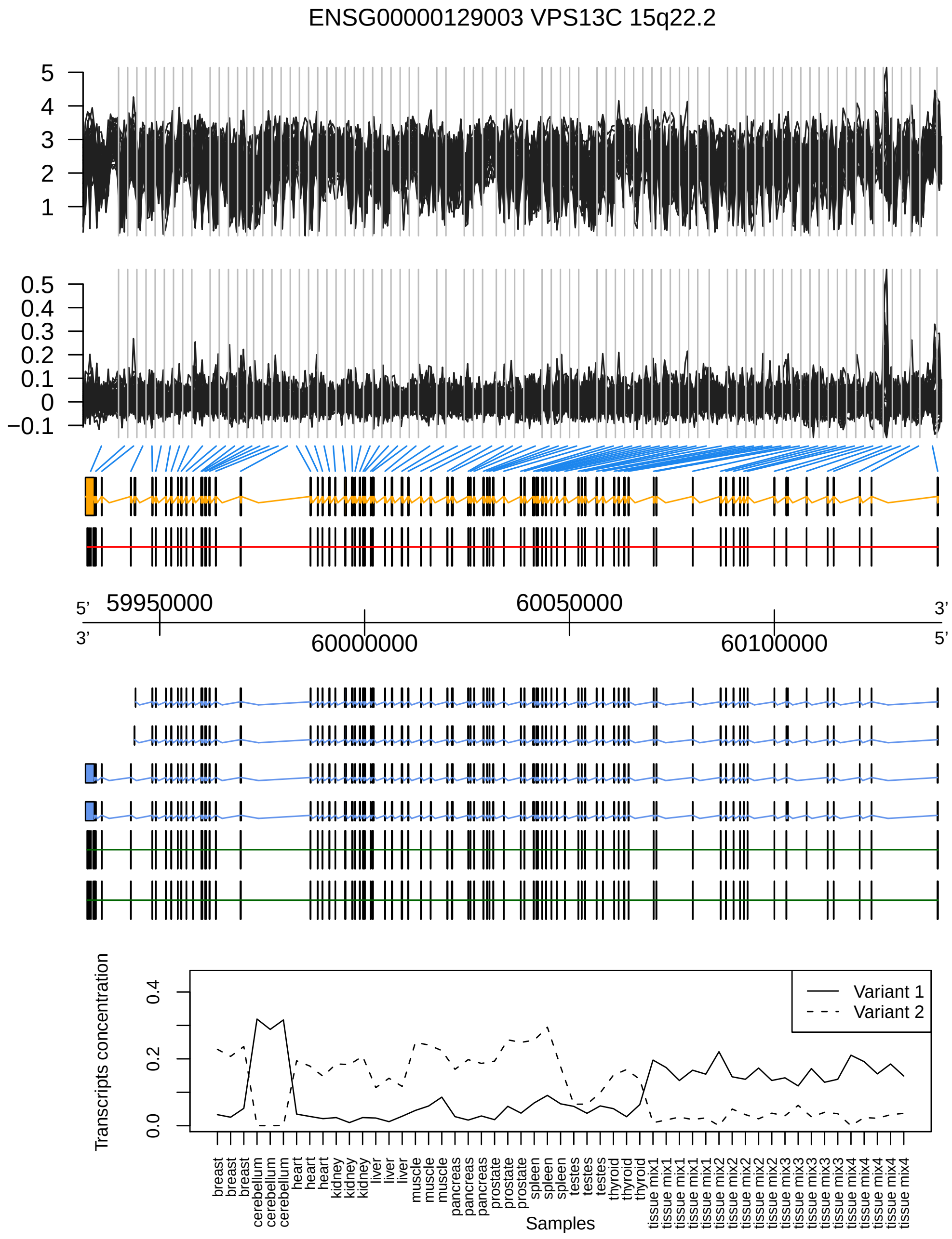

Figure 17: Results for VPS13C gene (Affymetrix sample dataset for human tissues, reverse strand). In the RT-PCR analysis of de la Grange *et al.*, (nar 2010), two isoforms were described that differed in the final exon, one isoform ended in exon 82 and the other isoform skipped that exon. Our results show two variants that differ in a cassette in exon 6, this exon cassette is known and is annotated in Ensembl release 51. Isoform variant 1, which includes exon 6, is predominant in cerebellum while the isoform variant 2, which excludes exon 6, is more abundant in the other tissues. Exon 82 is not mapped to this gene in the Brainarray cdf. In this case, the analysisi of the gene as a whole detects the cassette of exon 6 -confirmed by Ensembl- that is missed in the PCR analysis bacause of the location of the primers.

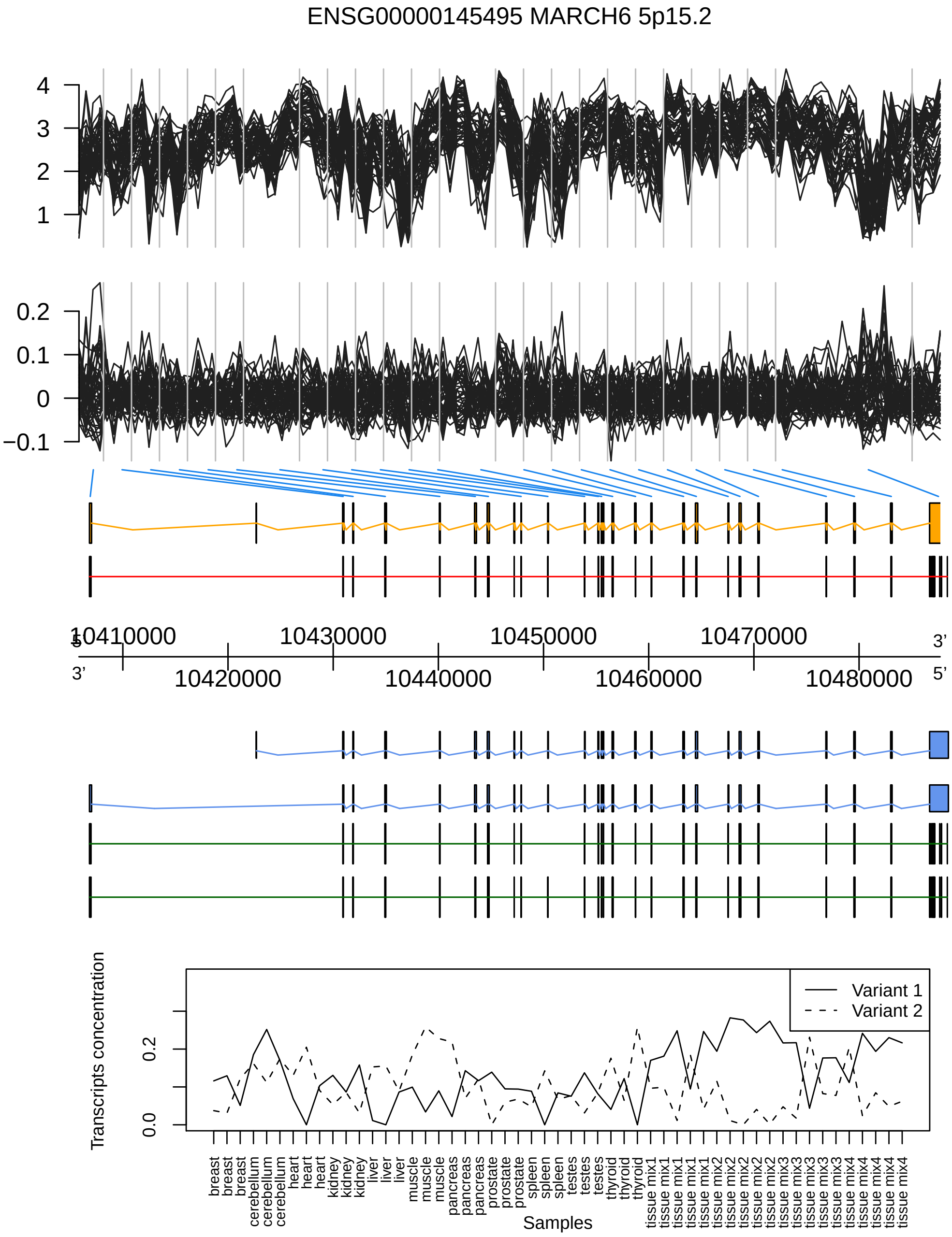

Figure 18: Results for MARCH6 gene (Affymetrix sample dataset for human tissues, direct strand). In the RT-PCR analysis of de la Grange *et al.*, (nar 2010), two isoforms were described that differed in the final exon, one short isoform that ended in exon 11 and other long isoform. Our results do not show the short isoform and only show a cassette in exon 10.

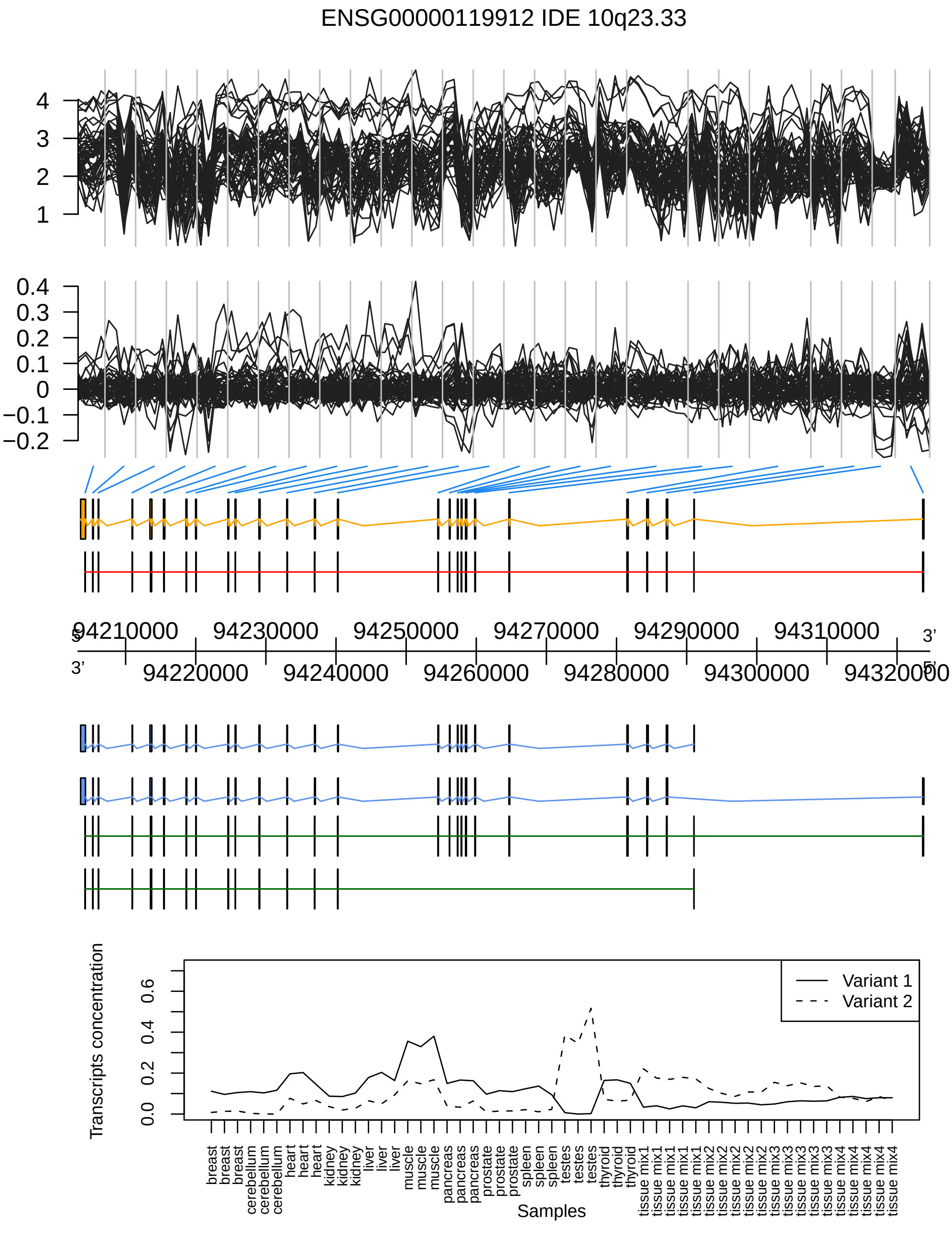

Figure 19: Results for IDE gene (Affymetrix sample dataset for human tissues, reverse strand). In the RT-PCR analysis of de la Grange *et al.*, (nar 2010), two isoforms were described that differed in the first exon, one isoform started in exon 18 and the other isoform skipped that exon. Our results also show a long isoform and a short isoform. The exon 18 is not mapped in Brainarray transcript CDF, but shorter isoform variant 2 includes the rest of the gene except plus a first exon that is wrongly assigned to it. Predicted concentrations are not concordant with RT-PCR.

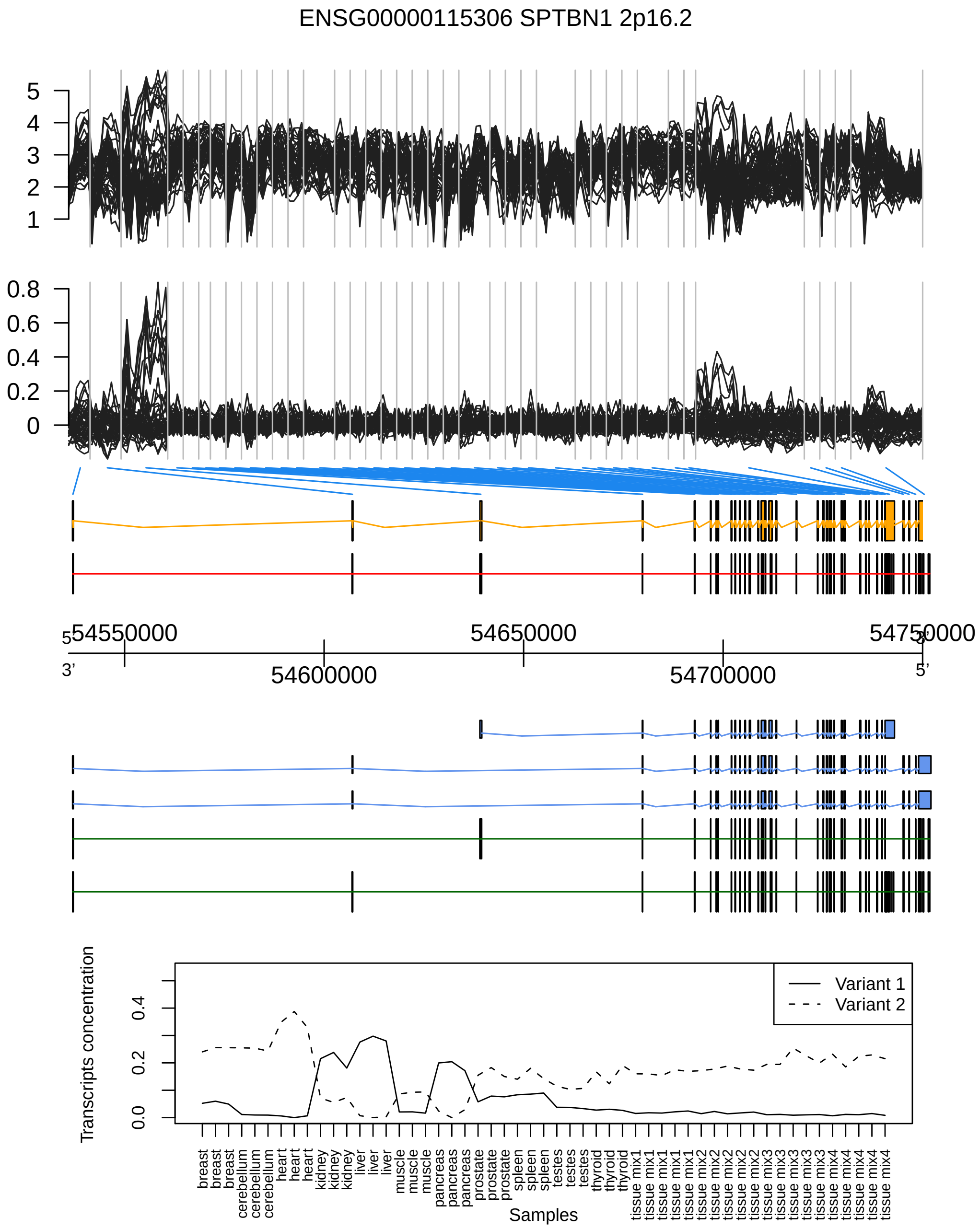

Figure 20: Results for SPTBN1 gene (Affymetrix sample dataset for human tissues, direct strand). In the RT-PCR analysis of de la Grange *et al.*, (nar 2010), two isoforms were described that differed in the first exon, one short isoform started in exon 4 and other isoform skipped that exon, the same happens in Ensembl release 51 annotation. Our results show that exon 4 as a cassette but not as first exon. Isoform variant 1, which includes exon 4, is predominant in liver (also in kidney and prostate not present in RT-PCR). Variant 2 concentrations are coherent with the short isoform.

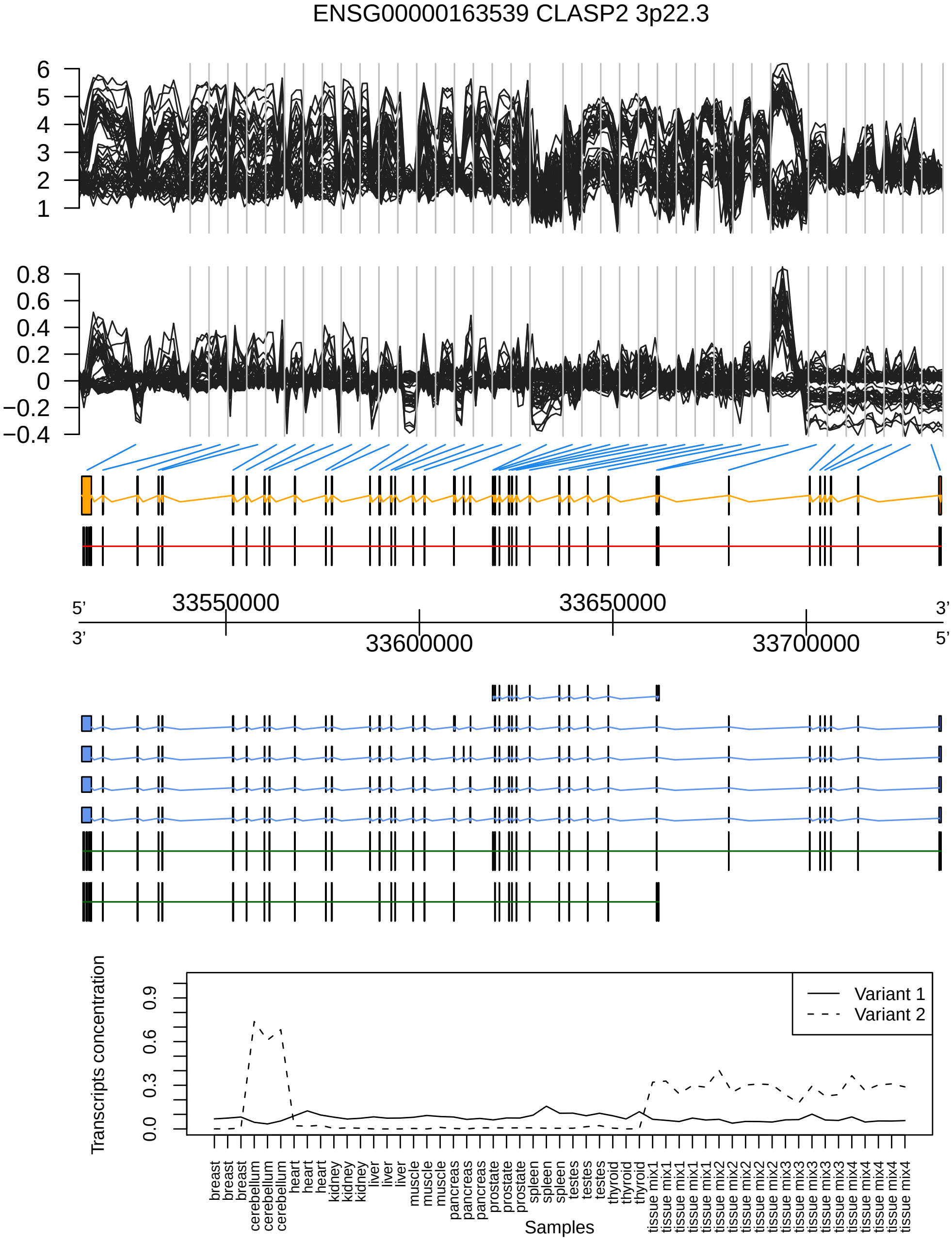

Figure 21: Results for CLASP2 gene (Affymetrix sample dataset for human tissues, reverse strand). In the RT-PCR analysis of de la Grange *et al.*, (nar 2010), two isoforms were described that differed in the first exon, one isoform started in exon 8 and the other isoform skipped that exon. Our results also show a long isoform and a short isoform and structure prediction matches. Short isoform variant 2, which includes exon 8, is predominant in cerebellum but neither in heart nor muscle as in the RT-PCR.

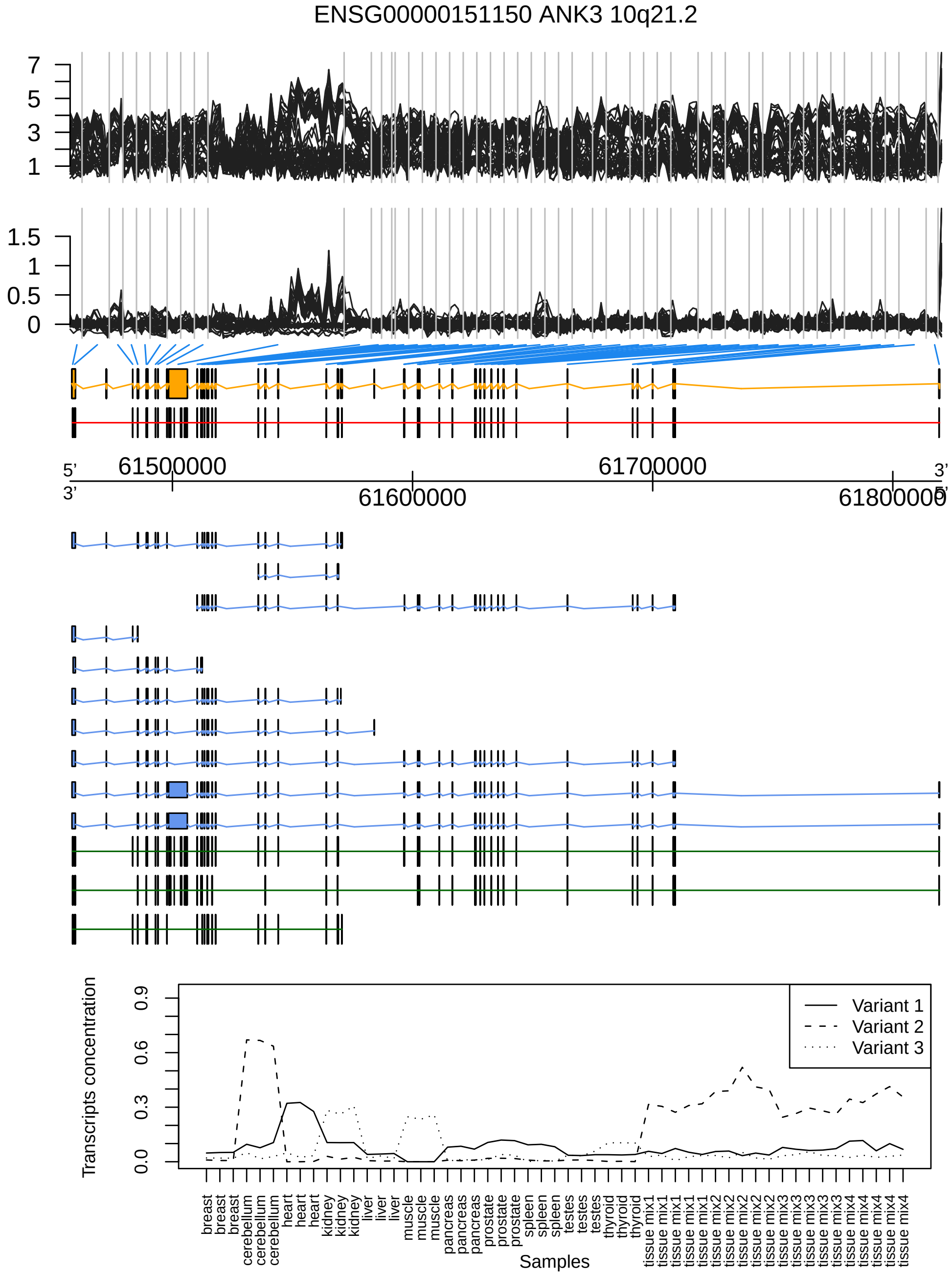

Figure 22: Results for ANK3 gene (Affymetrix sample dataset for human tissues, reverse strand). In the RT-PCR analysis of de la Grange *et al.*, (nar 2010), two isoforms were described that differed in a cassette in exon 16. Our results show three different isoforms. Isoform variant 1 is predominant in heart and is almost not present in the other tissues in concordance with RT-PCR. The other variant is not coherent with RT-PCR results.

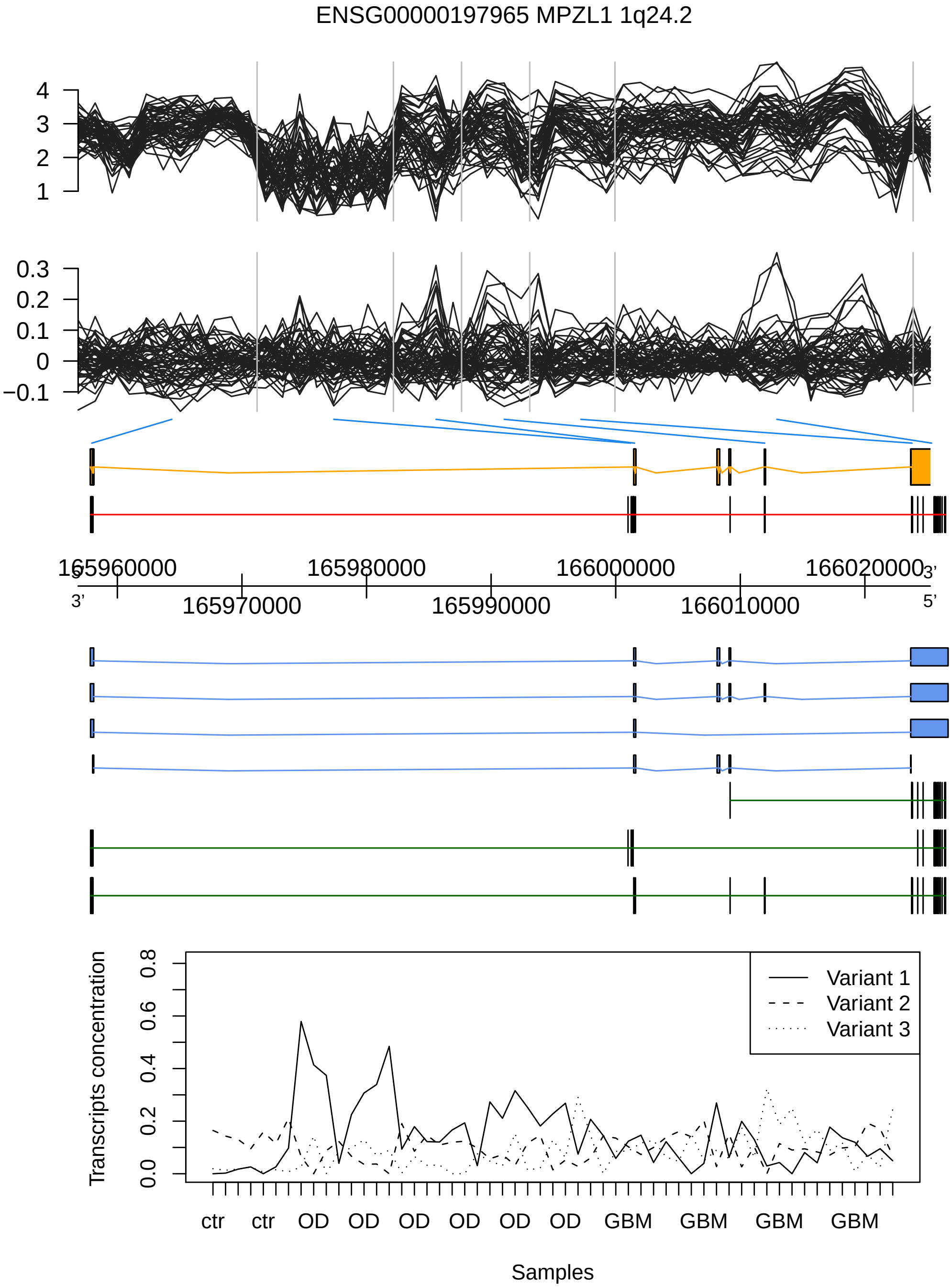

Figure 23: Results for MPZL1 gene (data from GSE9385) in a study of glial brain tumors in humans (French *et al.*, 2007). Samples 1 to 6 correspond to normal tissue, samples 7 to 32 to oligodendroglioma (OD) and samples 33 to 55 to glioblastoma multiforme (GBM). In (French *et al.*, 2007) this gene has two different transcripts in GBM samples. Residues of the RMA model do not show a clear separation between splice variants. Two of the exons of this gene are not represented in the exon array. The predicted concentration of variant 3 -a short isoform- seems to be overexpressed in most of OD and GBM samples if compared with normal samples.

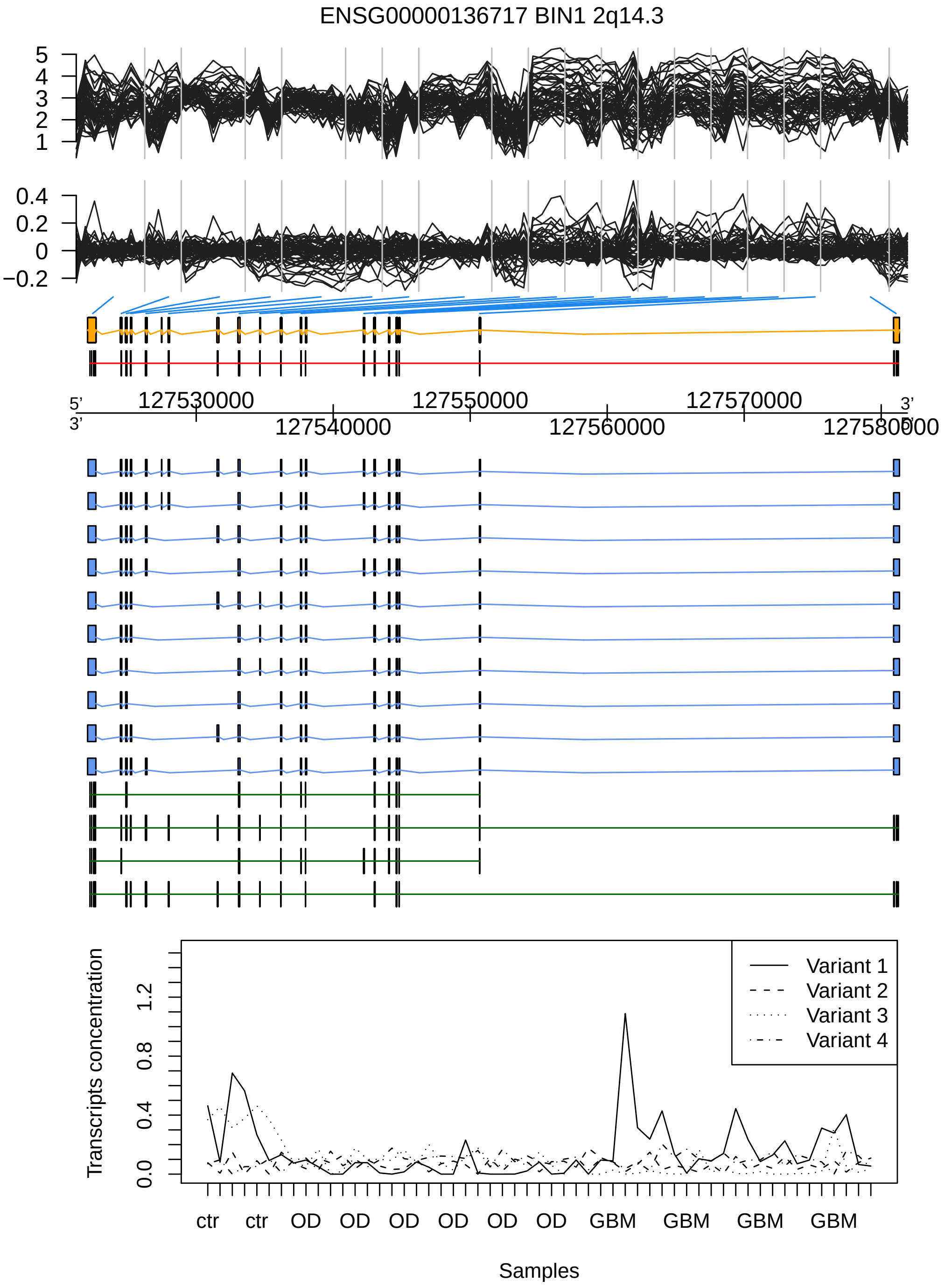

Figure 24: Results for BIN1 gene (data from GSE9385) (French *et al.*, 2007). In (French *et al.*, 2007) this gene has two different transcripts in OD and normal tissue samples. In GBM, three samples (out of five) do not show AS but a single band in PCR validation. The number of predicted transcripts is four. The first variant (that is predicted to have several skipped exons) is predominant in GBM. The third variant is predicted to be downregulated in OD and GBM samples if compared with normal tissues.

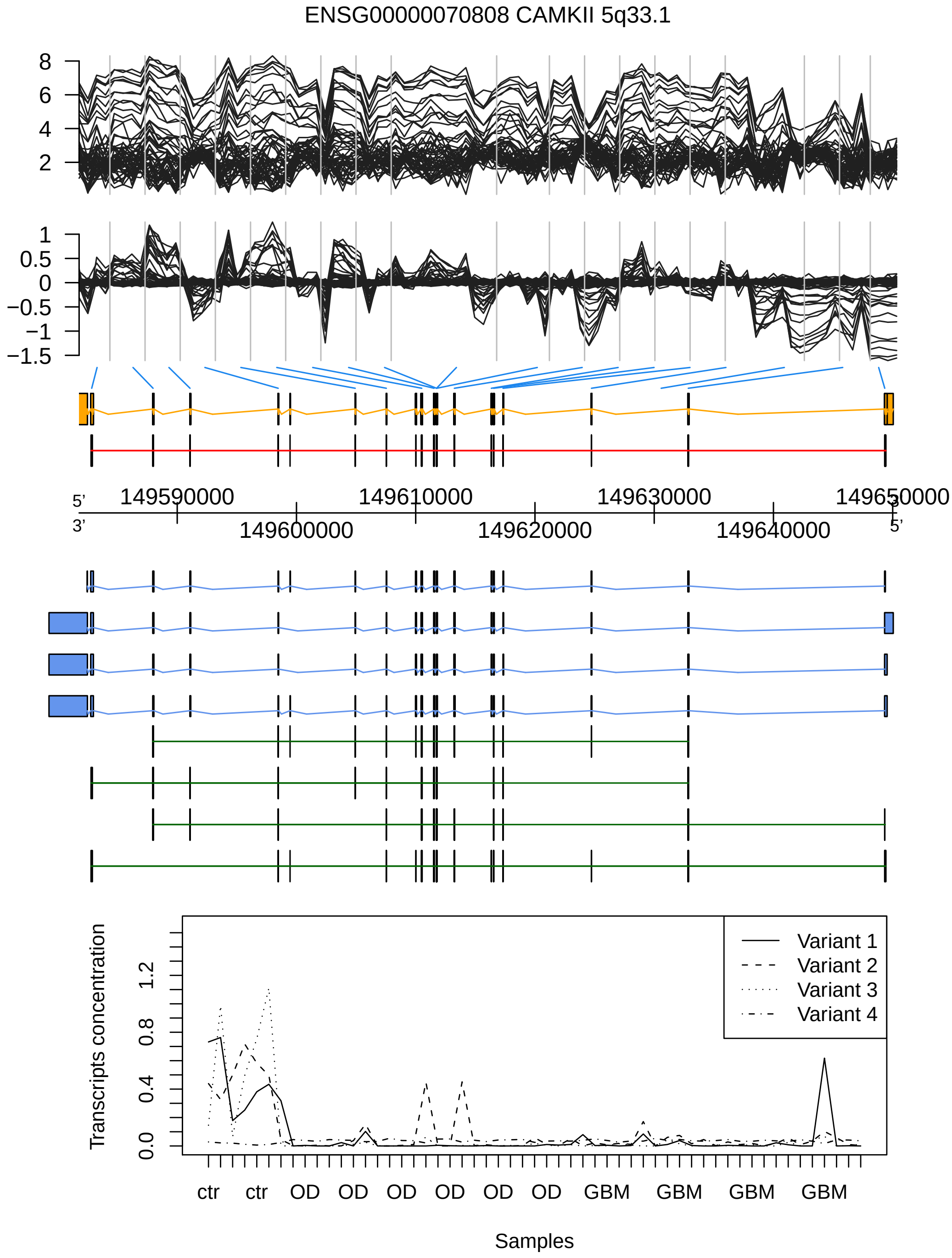

Figure 25: Results for CAMKII gene (data from GSE9385) (French *et al.*, 2007). The analysis of the residues of the RMA model show that this gene seems to have AS for exons 7, 13 and the last two ones. There are several exons that are not mapped in the array (6 and 9). Exon 7 does not show AS in the Ensembl release 51 database (all the transcripts have this exon and could be considered as constitutive). This gene is predicted to have four variants. They are expressed mainly in normal tissue and in some OD samples (as is displayed by PCR in (French *et al.*, 2007).

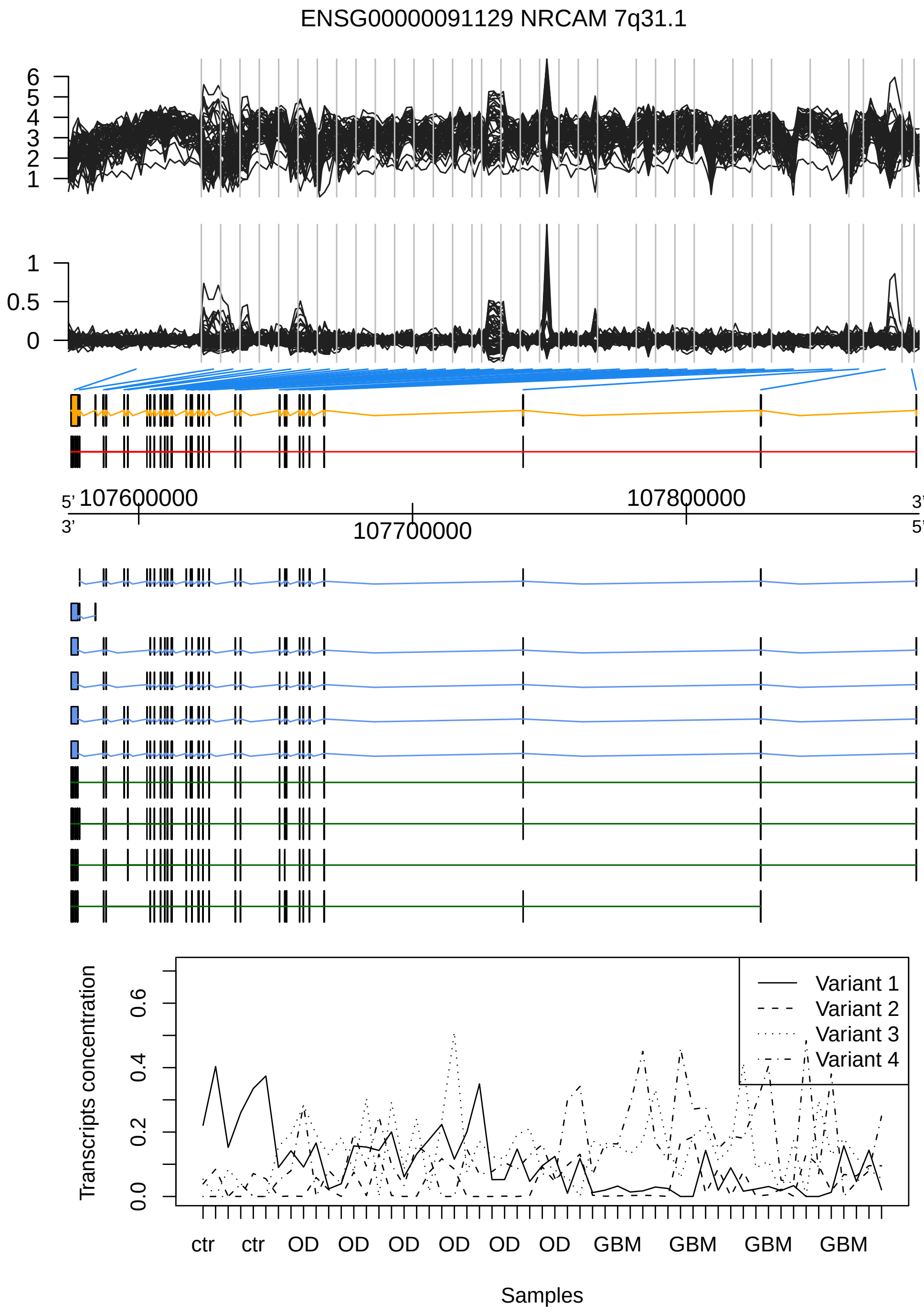

Figure 26: Results for NRCAM gene (data from GSE9385) (French *et al.*, 2007). This gene shows (in the validated PCR) AS in the three different tissues (normal, OD and GBM) and it is difficult to discern if any of the variants is more abundant in a particular tissue. The residues show that probably, there is AS in the set of probes 2,3,4,7 and 17 among others.

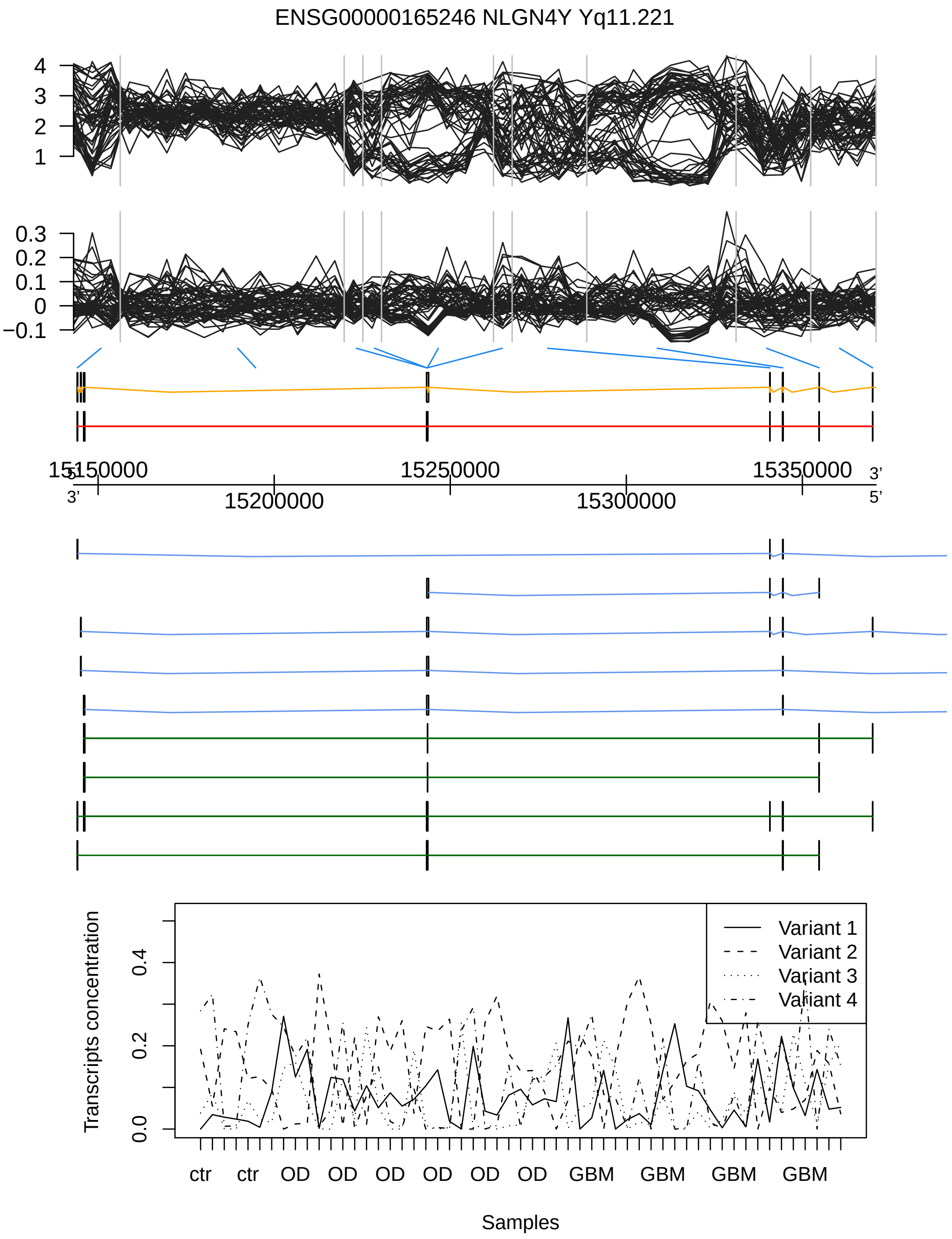

Figure 27: Results for NLGN4Y gene (data from GSE9385) (French *et al.*, 2007). Residues (and even raw expression values) show that there is AS in exon 2 and 4. Residues show that the splicing event in exon 4 occur only in the downstream probes (probably and alternative donor site). In this case, probe coherence will hide these AS events since they occur within the probeset. PCR validation show different isoforms but none of them are able to discern between the different groups.

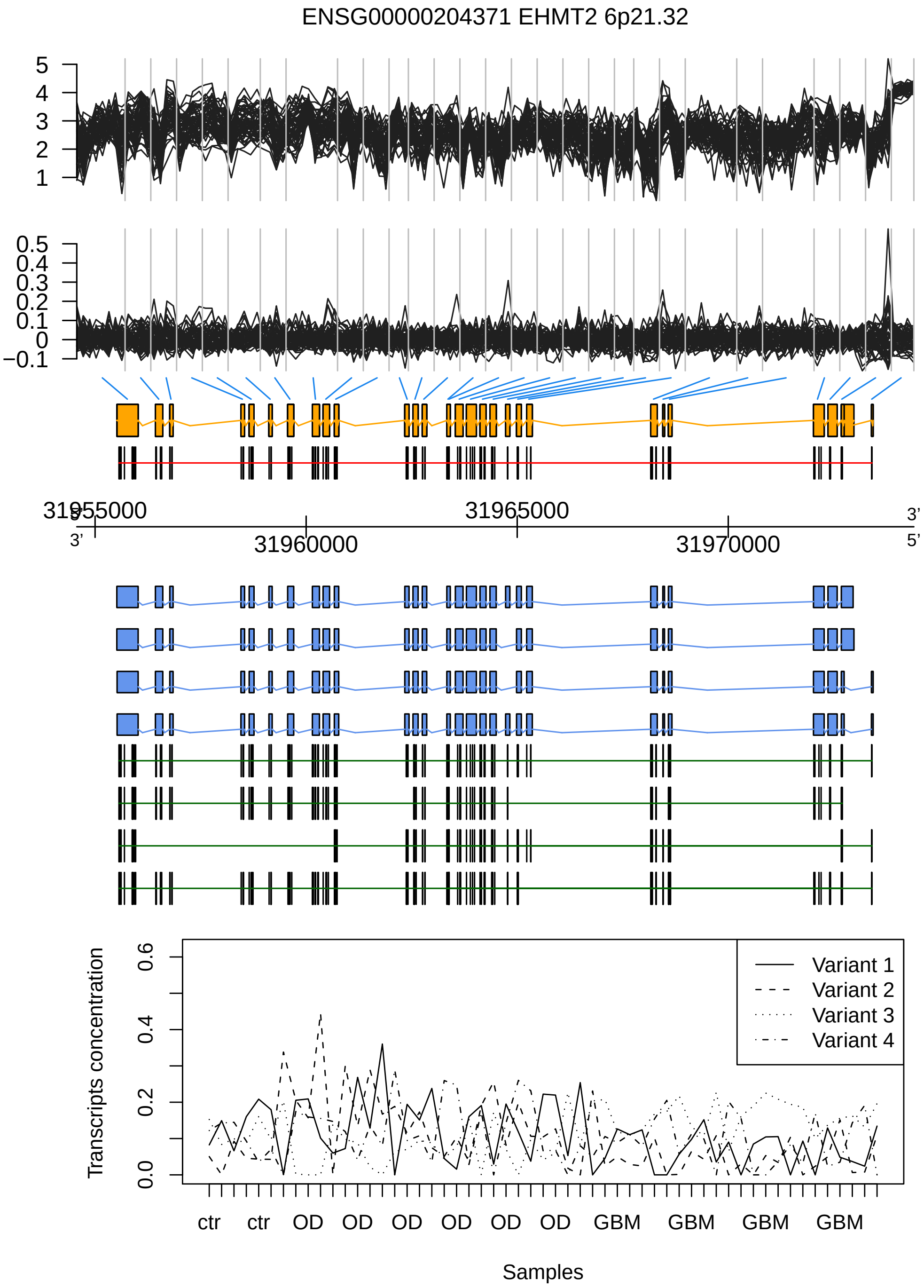

Figure 28: Results for EHMT2 gene (data from GSE9385) (data from GSE9385) (French *et al.*, 2007). This gene presents at least four different transcripts in the PCR validation. One of them appears only in one of the GBM samples but not in any of the other samples. SPACE is not able to discern it.

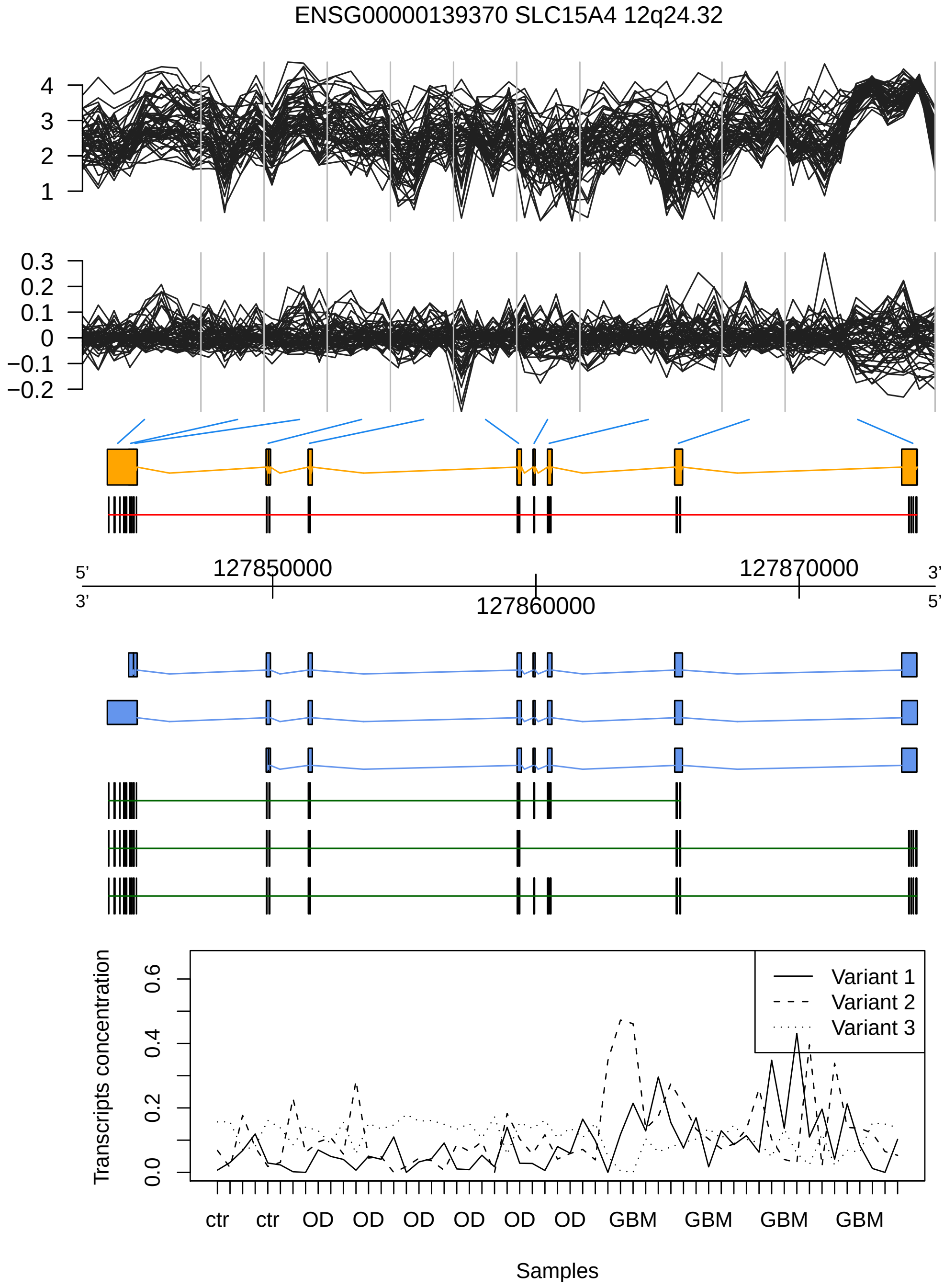

Figure 29: Results for SLC15A4 gene (data from GSE9385) (French *et al.*, 2007). SPACE algorithm predicts three transcripts for this gene. One of them is able to discern between GBM samples and OD or control samples. This result is also shown in the PCR figures (French *et al.*, 2007)

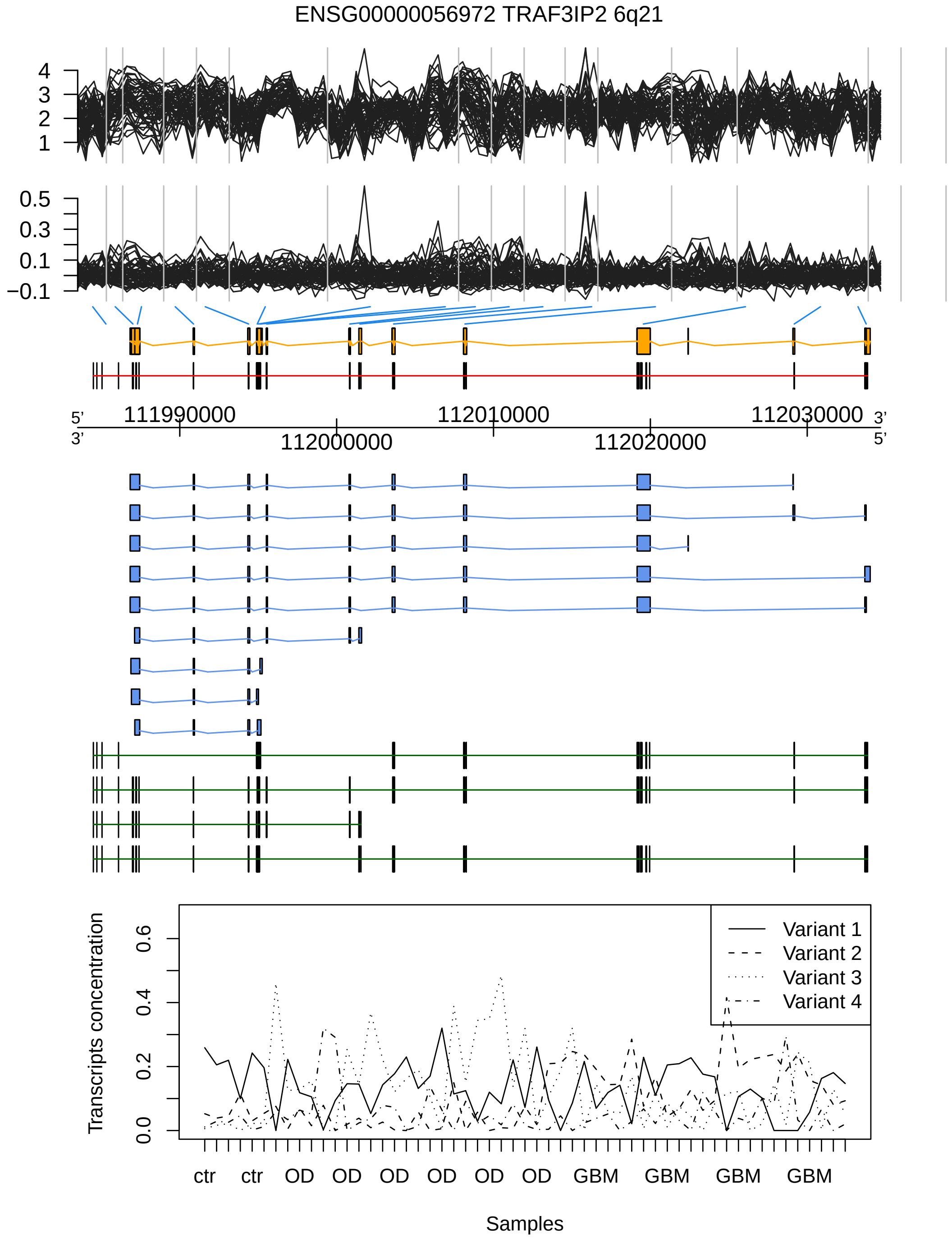

Figure 30: Results for TRAF3IP2 gene (data from GSE9385) (French *et al.*, 2007). RT-PCR validation shows two different transcripts, one of them with very low concentration levels. These transcripts are expressed in the three groups of samples and are not able to distinguish between them. SPACE predicts four transcripts.

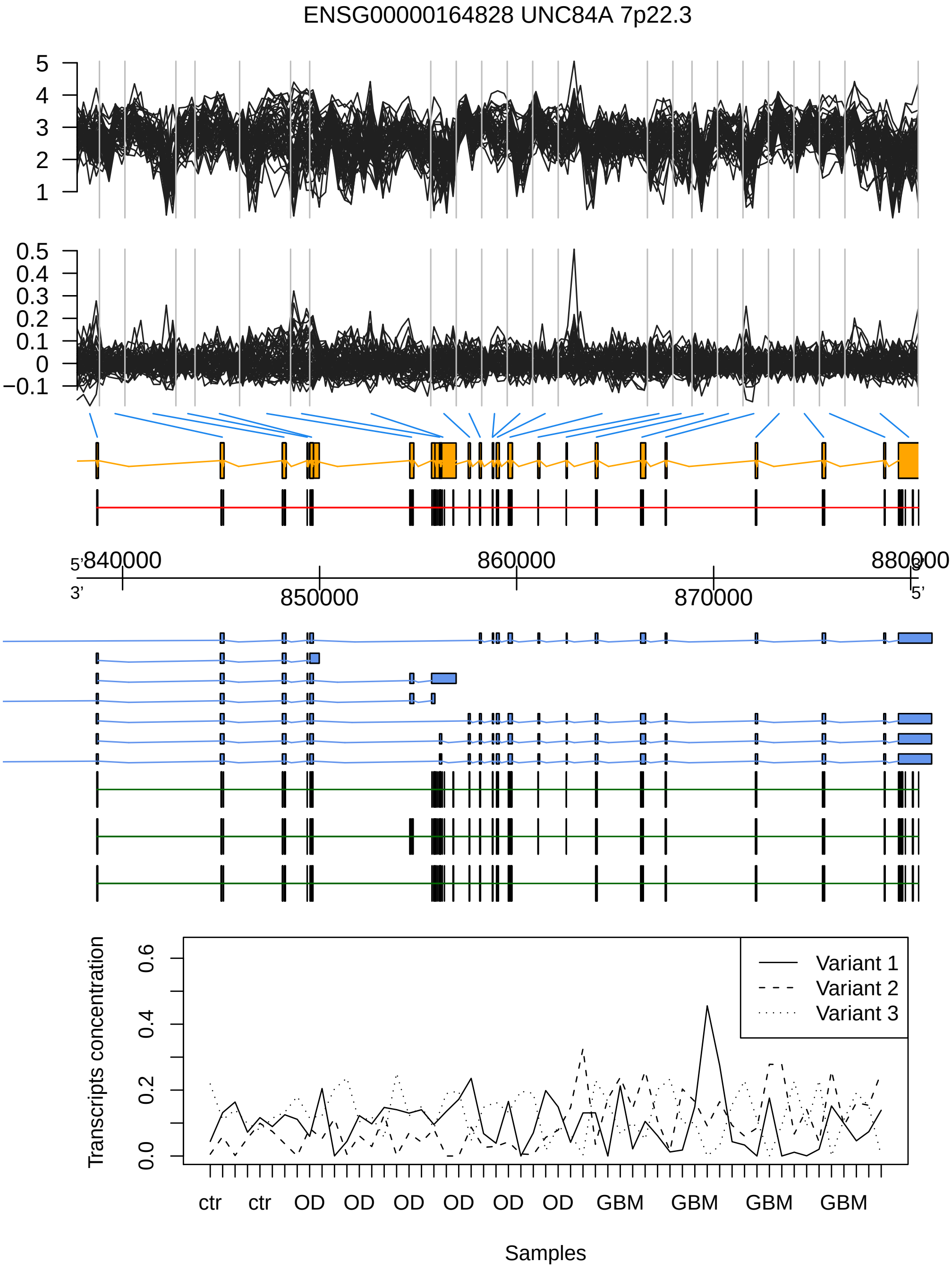

Figure 31: Results for UNC84A gene (data from GSE9385) (French *et al.*, 2007). PCR shows three transcripts. One of them is overexpressed in four of the five GBM samples. SPACE also predicts three transcripts. The second transcript -the largest isoform- is also overexpressed in the GBM samples.

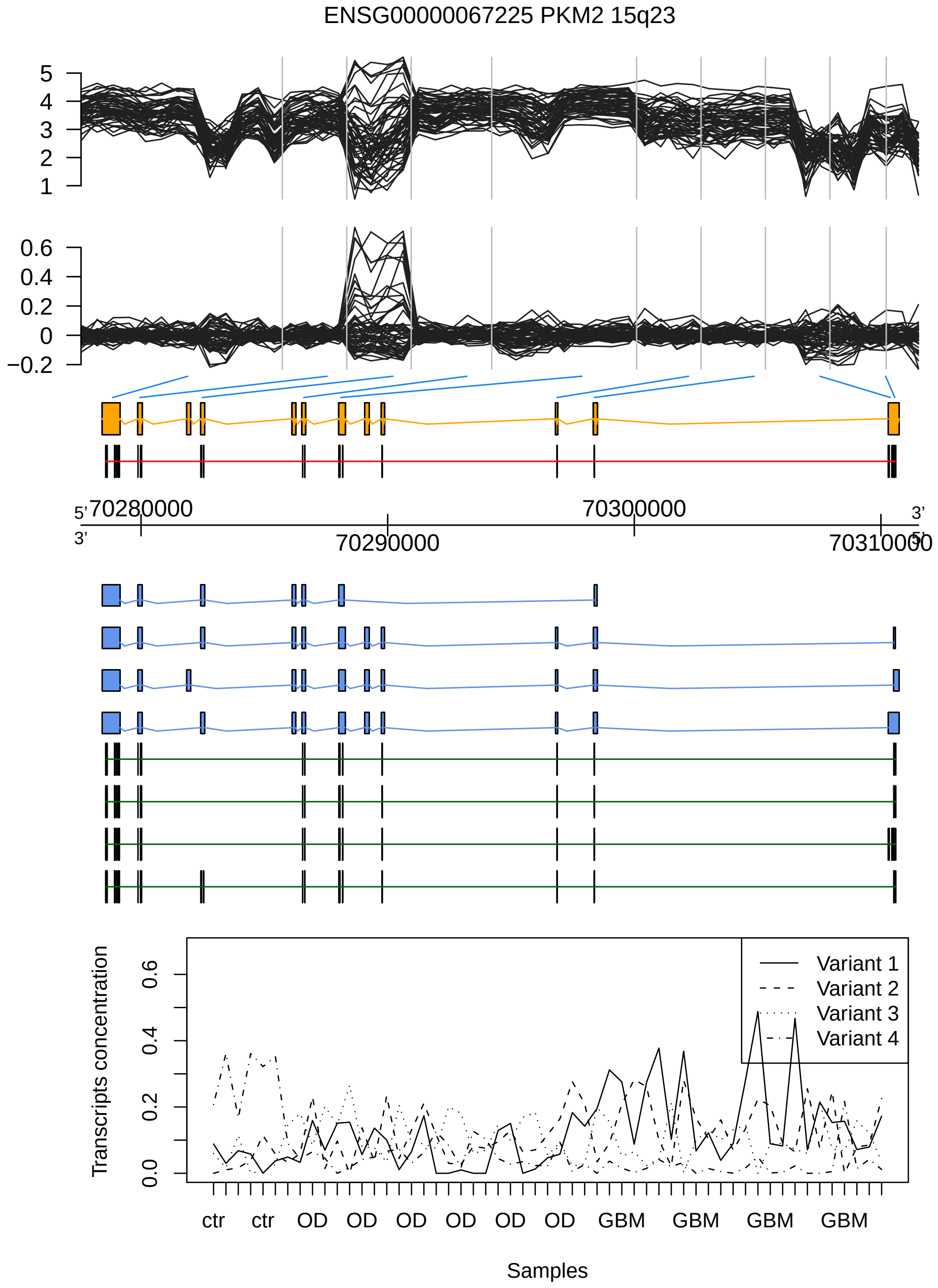

Figure 32: Results for PKM2 gene (data from GSE9385) (French *et al.*, 2007). This gene is in the reverse strand. Consulting the UCSC genome browser the 9th and the 10th exons are, in the figure, the 3rd and the 4th -counting from left to right. The residues of the RMA model show that there is AS in the probes which correspond to exon 9 (the 3rd group of probes starting from left to right). SPACE also predicts an alternative donor site in the last group which correspond to exon 1. Exon 9 and 10 are referred to have alternative splicing events (Christofk *et al.*, Nature 452: 230-233, 2008). In the fourth graph of these figure (black and red vertical bars) it can be seen that no probes that target the 10th exon (3rd from left to right) are included in the exon array -in fact, there are probes that target this exon but because of cross-hybridization or other reasons, they have been removed from the Brainarray cdf. There are however probes that target the 9th exon (4th from left to right, 3rd group of probes). In fact, this is the most prominent splicing event in this gene (the residuals of the RMA model of this gene are especially large for these probes). Only predicted variant 1 includes this exon, and this variant is expressed mainly in normal tissue (the first six control samples). Variant 2 and 3 are more expressed in tumor samples.

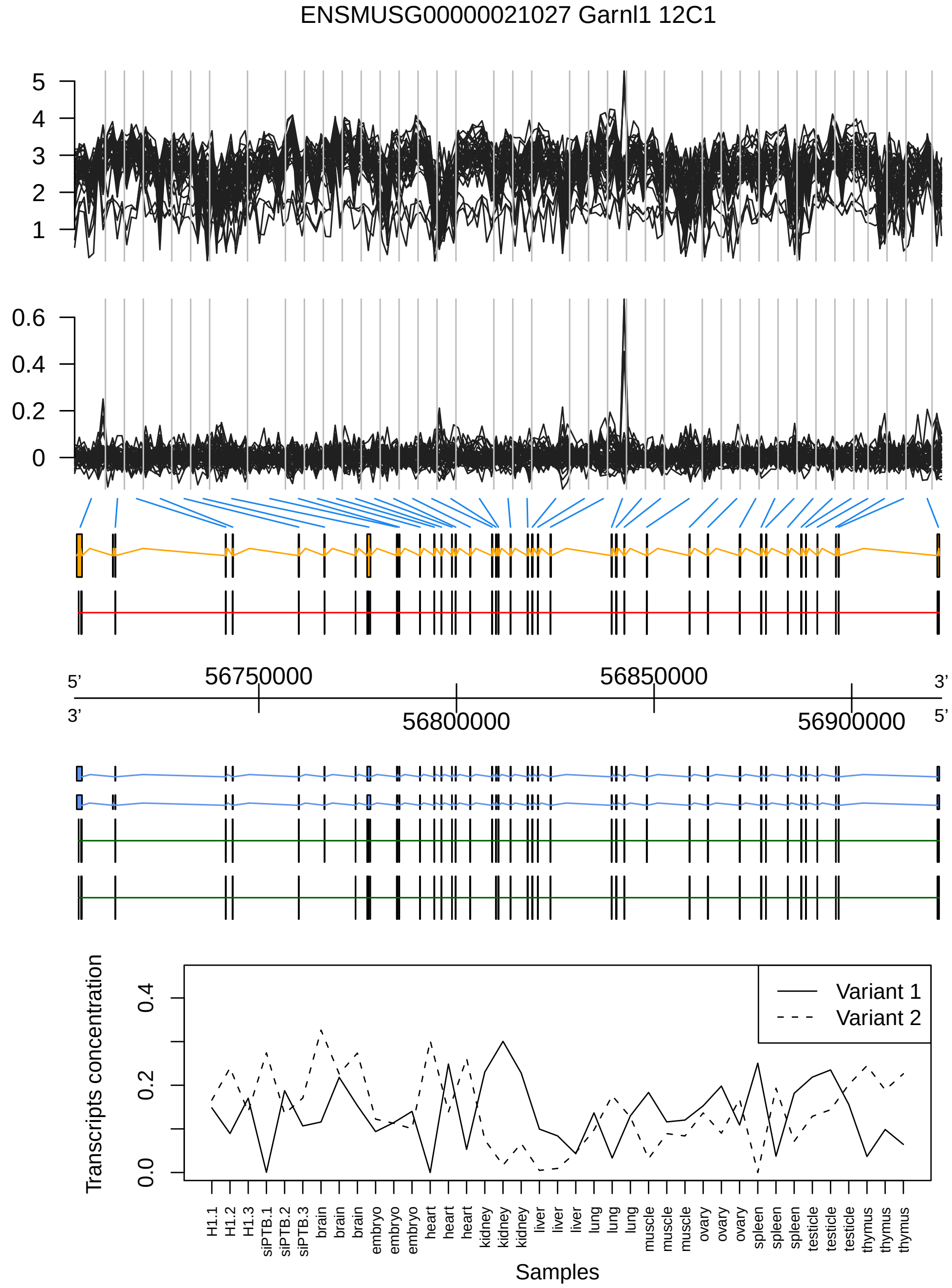

Figure 33: Results for Garnl1 gene (data from GSE11344) (Xing *et al.*, 2008). Samples 1 to 3 correspond to normal tissue and samples 4 to 7 present PTB gene depletion in mice. In addition to these samples, we have included in the analysis data from 11 different tissues provided by Affymetrix (samples 7 to 39, three replicates for each tissue). In this case, the two predicted variants are not able to distinguish between the two groups (within the first 6 samples).

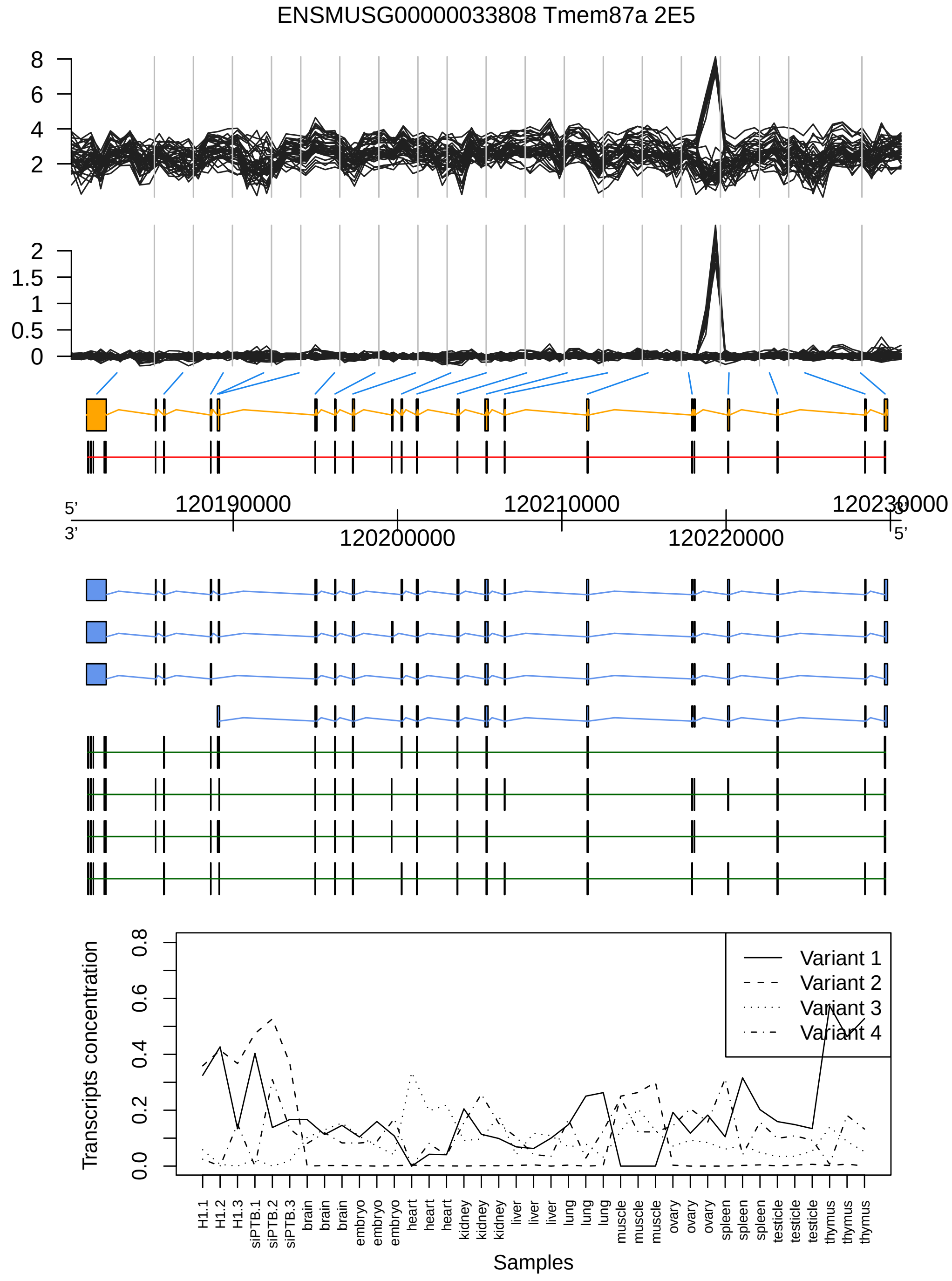

Figure 34: Results for Tmem87a gene (data from GSE11344) (Xing *et al.*, 2008). Samples 1 to 3 correspond to normal tissue and samples 4 to 7 present PTB gene depletion in mice. In addition to these samples, we have included in the analysis data from 11 different tissues provided by Affymetrix (samples 7 to 39, three replicates for each tissue). Exons 12 and 13 -around position 120,2Mb are predicted to be mutually exclusive. If the concentrations are correct, the two PCR bands must be proportional to variant 1 plus variant 4 and variant 2 plus variant 3. The difference in the intensity of the PCR bands is very weak. In addition, the bands are only 3 nt apart. It is difficult to distinguish both isoforms using PCR.

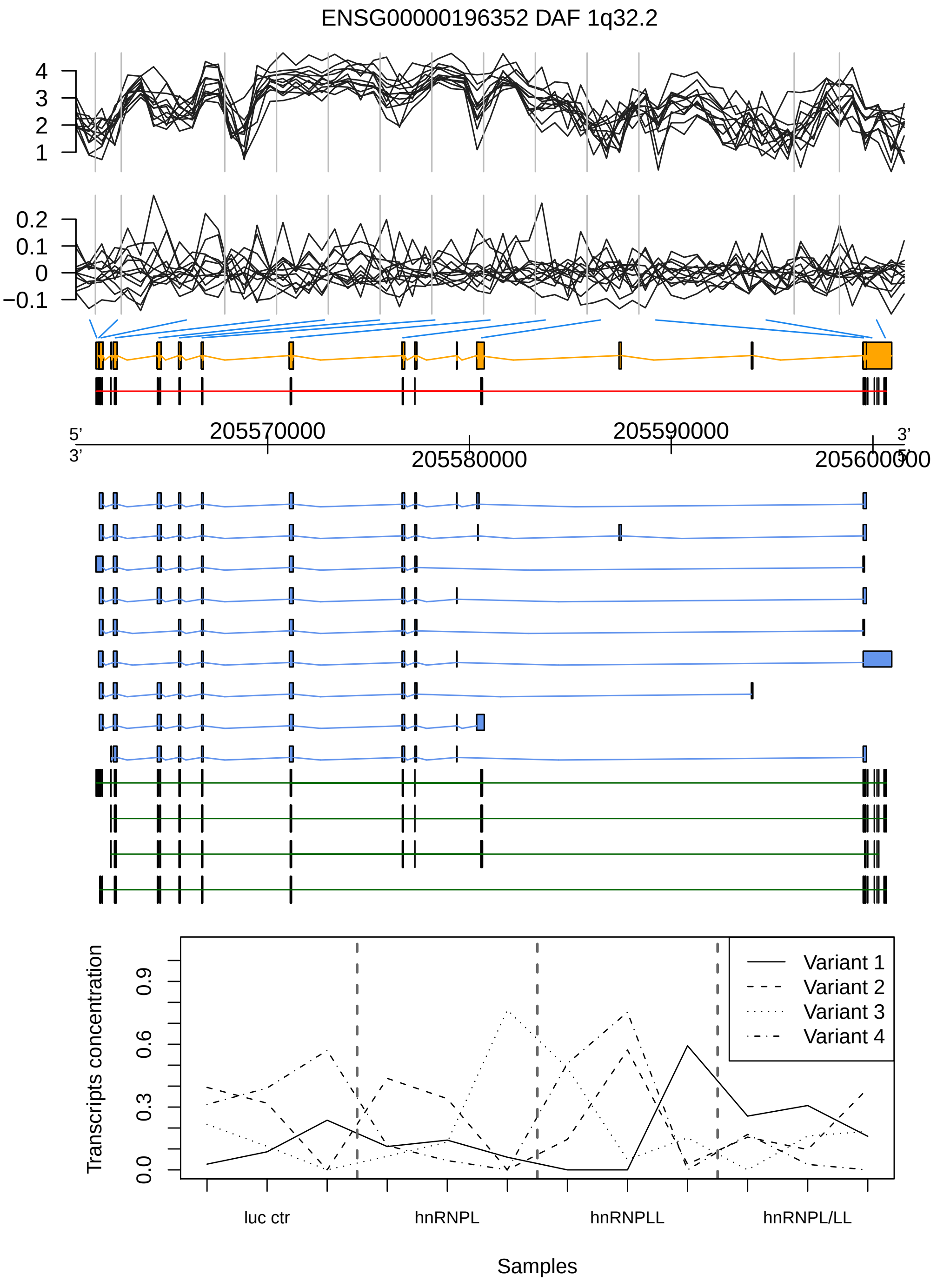

Figure 35: Results for DAF gene (data from GSE8945) (Hung *et al.*, 2008). hnRNP L has been identified in the cited reference as a global regulator of AS. hnRNP LL is a paralog of hnRNP. Samples consist of three technical replicates of each condition: luciferase control, hnRNP L, hnRNP LL and hnRNP L/LL. hnRNP L, hnRNP LL and hnRNP L/LL are knockouts of hnRNP L, hnRNP LL and hnRNP L and LL respectively. In this reference, all the probes of the exon array were mapped against the NCBI annotation of the human genome. This annotation is quite different to Ensembl release 51. This gene has an intron retention, validated by PCR. However, the Ensembl mapping does no include the probes within this intron and it is not possible to discover this AS event.

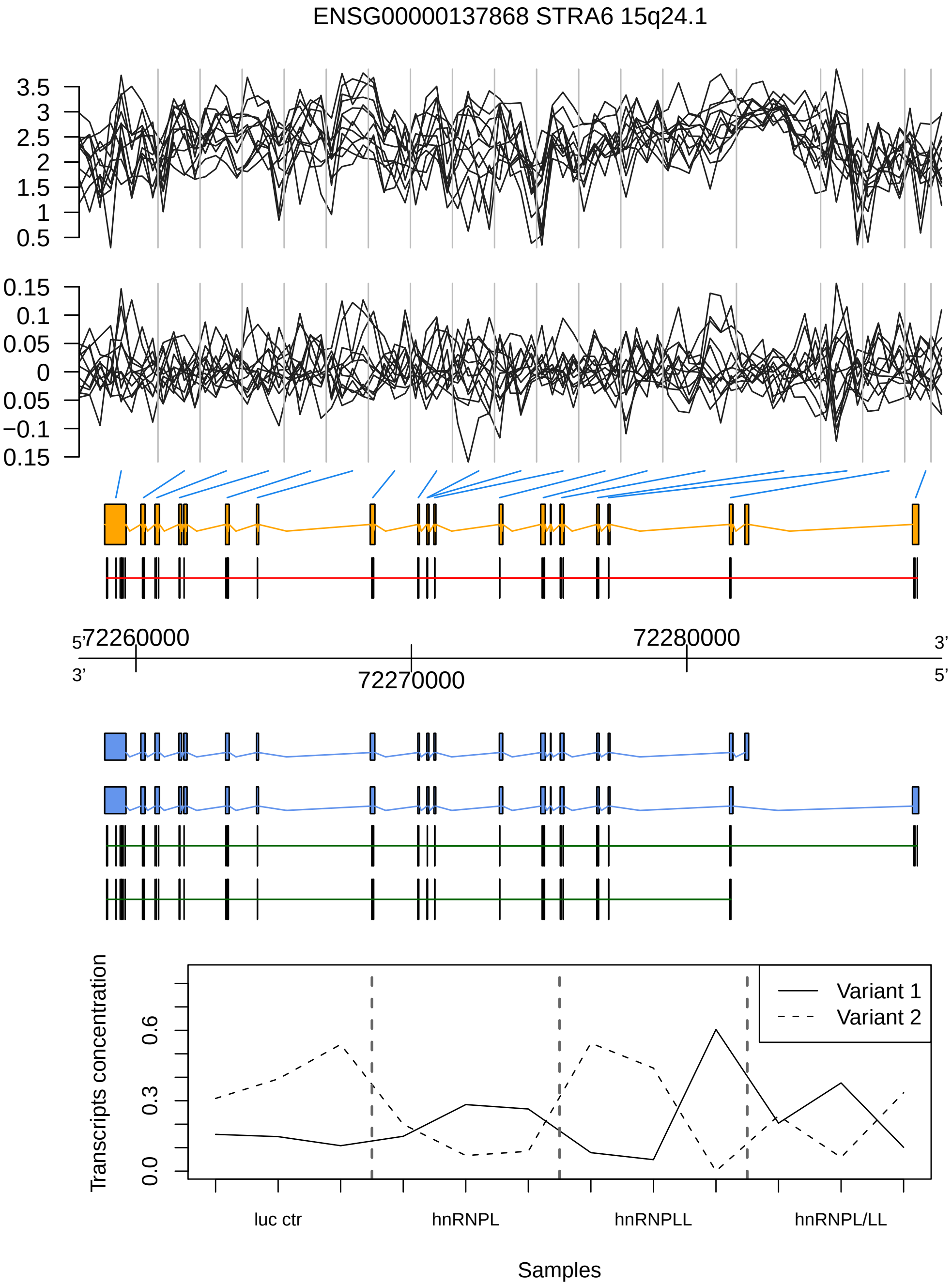

Figure 36: Results for STRA6 gene (data from GSE8945) (Hung *et al.*, 2008). hnRNP L has been identified in the cited reference as a global regulator of AS. hnRNP LL is a paralog of hnRNP. Samples consist of three technical replicates of each condition: luciferase control, hnRNP L, hnRNP LL and hnRNP L/LL. hnRNP L, hnRNP LL and hnRNP L/LL are knockouts of hnRNP L, hnRNP LL and hnRNP L and LL respectively. In this reference, all the probes of the exon array were mapped against the NCBI annotation of the human genome. As it happens in the previous gene, intron probes are not mapped in Ensembl release 51 and it is not possible to unravel this AS event.

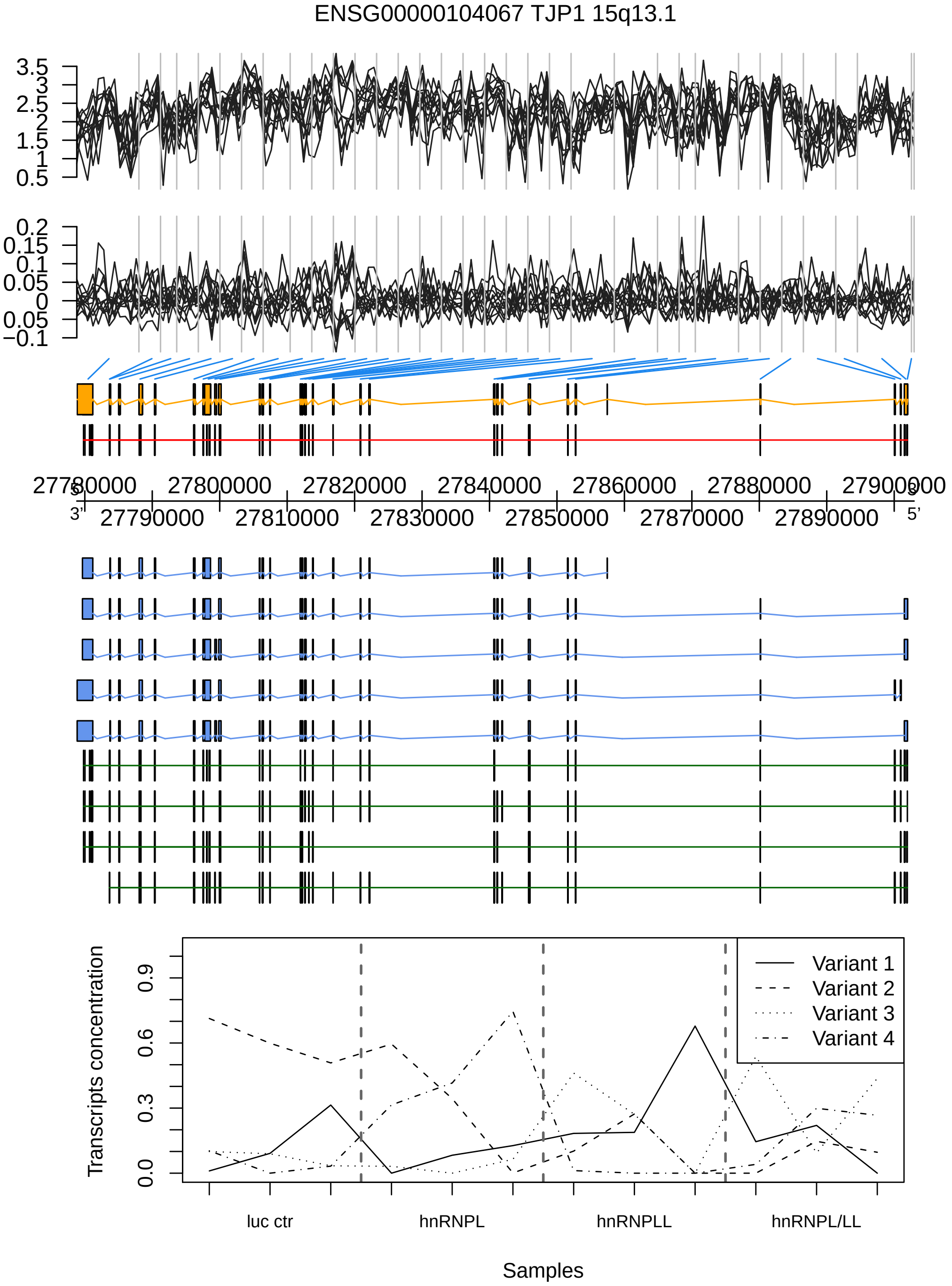

Figure 37: Results for TJP1 gene (data from GSE8945) (Hung *et al.*, 2008). hnRNP L has been identified in the cited reference as a global regulator of AS. hnRNP LL is a paralog of hnRNP. Samples consist of three technical replicates of each condition: luciferase control, hnRNP L, hnRNP LL and hnRNP L/LL. hnRNP L, hnRNP LL and hnRNP L/LL are knockouts of hnRNP L, hnRNP LL and hnRNP L and LL respectively. The residues show a AS event around the position 27800000 (blue lines indicate the relationship between the residues and the real position of the probes).

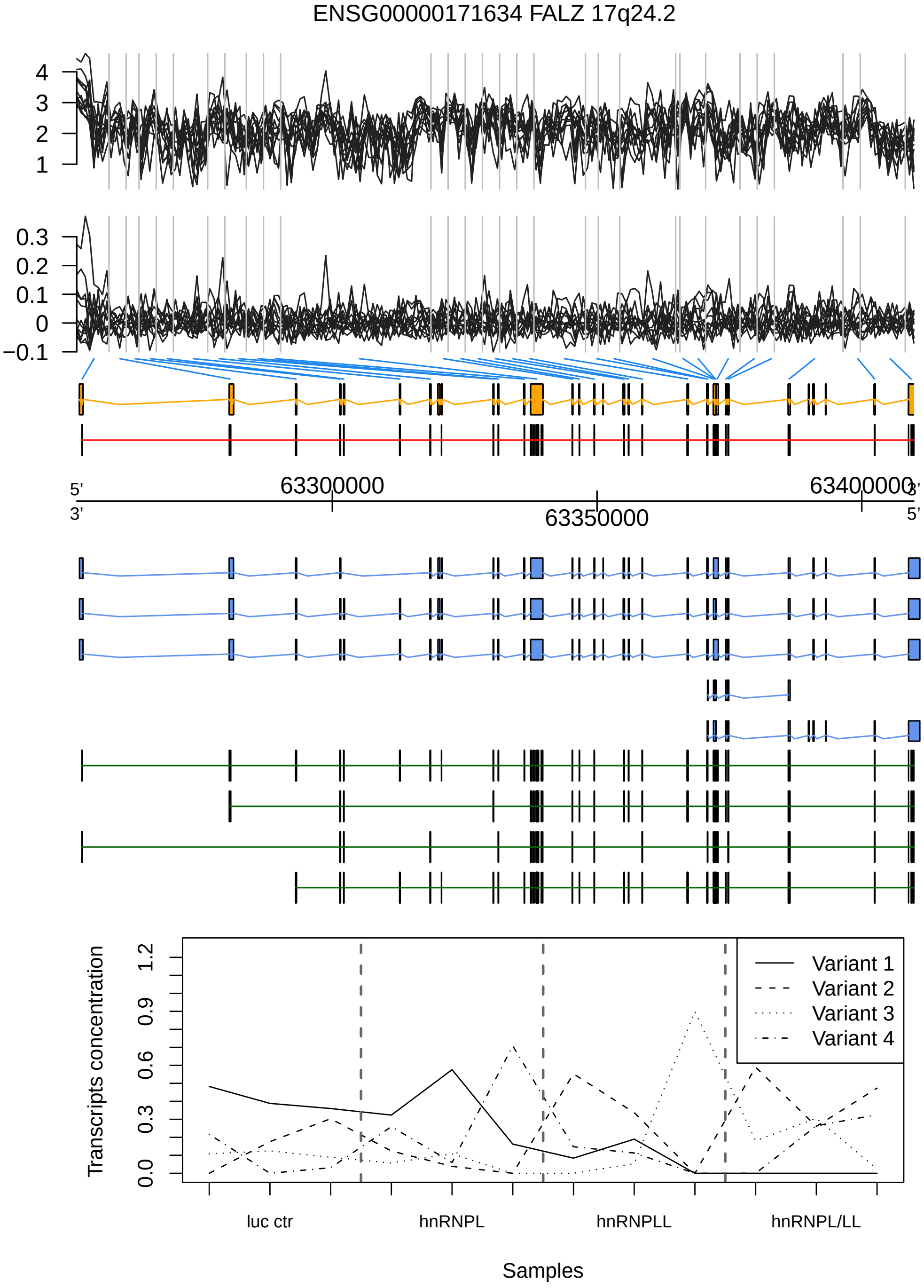

Figure 38: Results for FALZ gene (data from GSE8945) (Hung *et al.*, 2008). hnRNP L has been identified in the cited reference as a global regulator of AS. hnRNP LL is a paralog of hnRNP. Samples consist of three technical replicates of each condition: luciferase control, hnRNP L, hnRNP LL and hnRNP L/LL. hnRNP L, hnRNP LL and hnRNP L/LL are knockouts of hnRNP L, hnRNP LL and hnRNP L and LL respectively. Brainarray mapping does not include any exon with less than three probes. In this gene, the discovered AS event involves exons that has only one probe. Therefore, it is not possible to reproduce the results of the reference using these Brainarray CDF's (Mapping) files. Unfortunately, it also occurs for other genes included in the reference (PARK7, MYL6, FAM48A, PAPOLA).

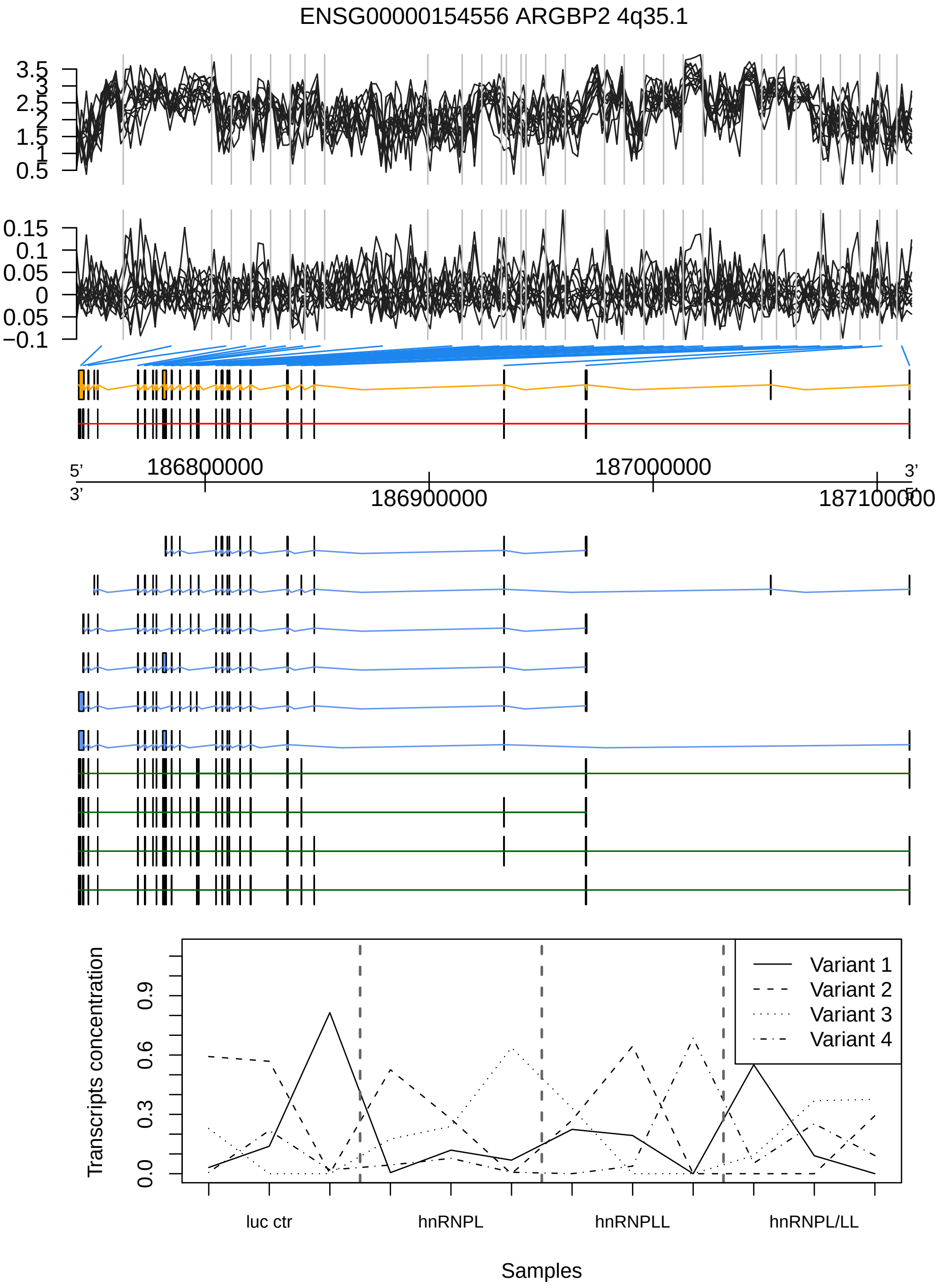

Figure 39: Results for ARGBP2 gene (data from GSE8945) (Hung *et al.*, 2008). hnRNP L has been identified in the cited reference as a global regulator of AS. hnRNP LL is a paralog of hnRNP. Samples consist of three technical replicates of each condition: luciferase control, hnRNP L, hnRNP LL and hnRNP L/LL. hnRNP L, hnRNP LL and hnRNP L/LL are knockouts of hnRNP L, hnRNP LL and hnRNP L and LL respectively. Residues does not show a clear AS event. In the reference, RT-PCR bands for the alternative isoforms are very weak and do not distinguish between the groups.

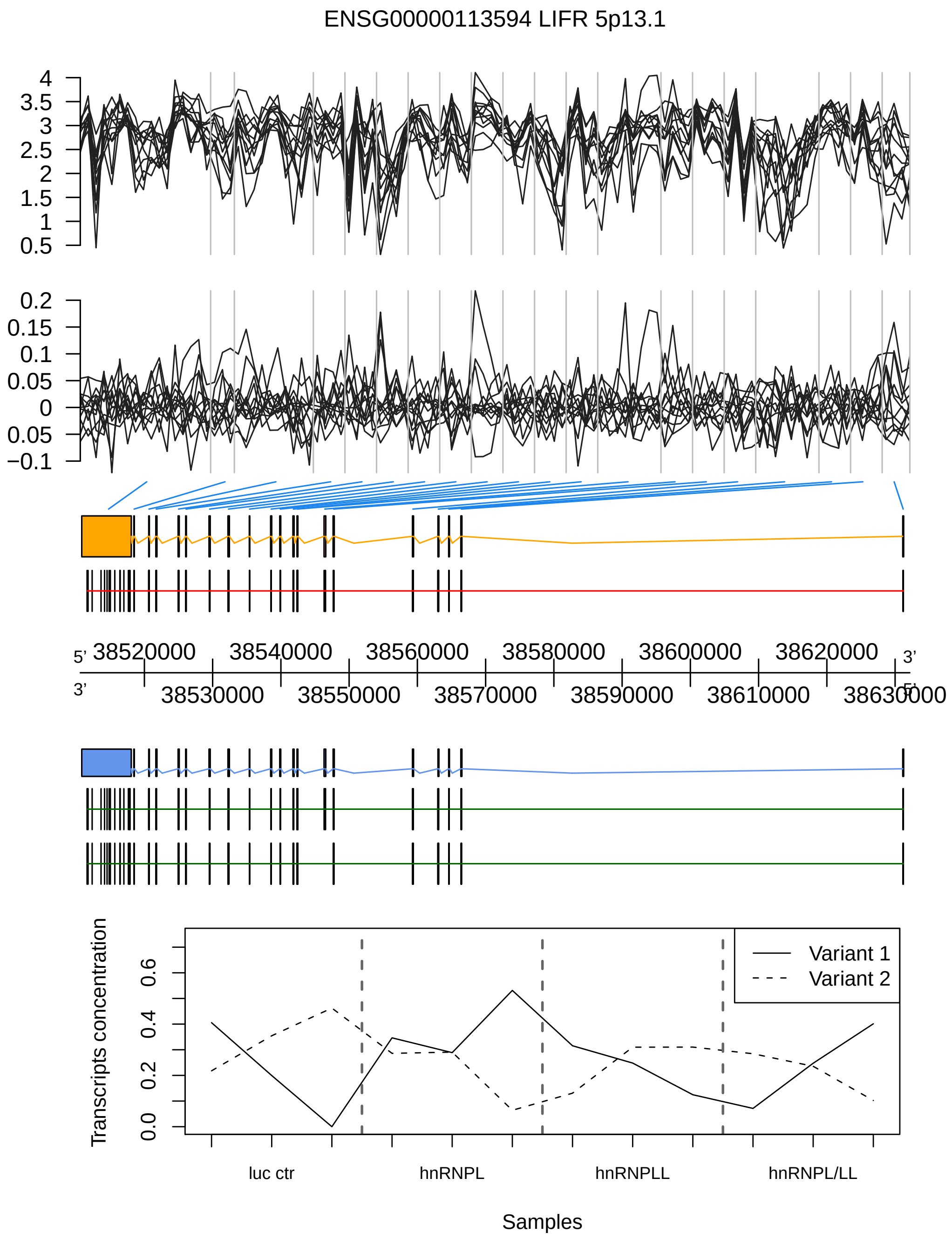

Figure 40: Results for LIFR gene (data from GSE8945) (Hung *et al.*, 2008). hnRNP L has been identified in the cited reference as a global regulator of AS. hnRNP LL is a paralog of hnRNP. Samples consist of three technical replicates of each condition: luciferase control, hnRNP L, hnRNP LL and hnRNP L/LL. hnRNP L, hnRNP LL and hnRNP L/LL are knockouts of hnRNP L, hnRNP LL and hnRNP L and LL respectively. In the reference, relative abundance of each of the isoforms (in RT-PCR) are quite similar to each other, and therefore difficult to distinguish using SPACE. Residues does not show any clear event to pinpoint.

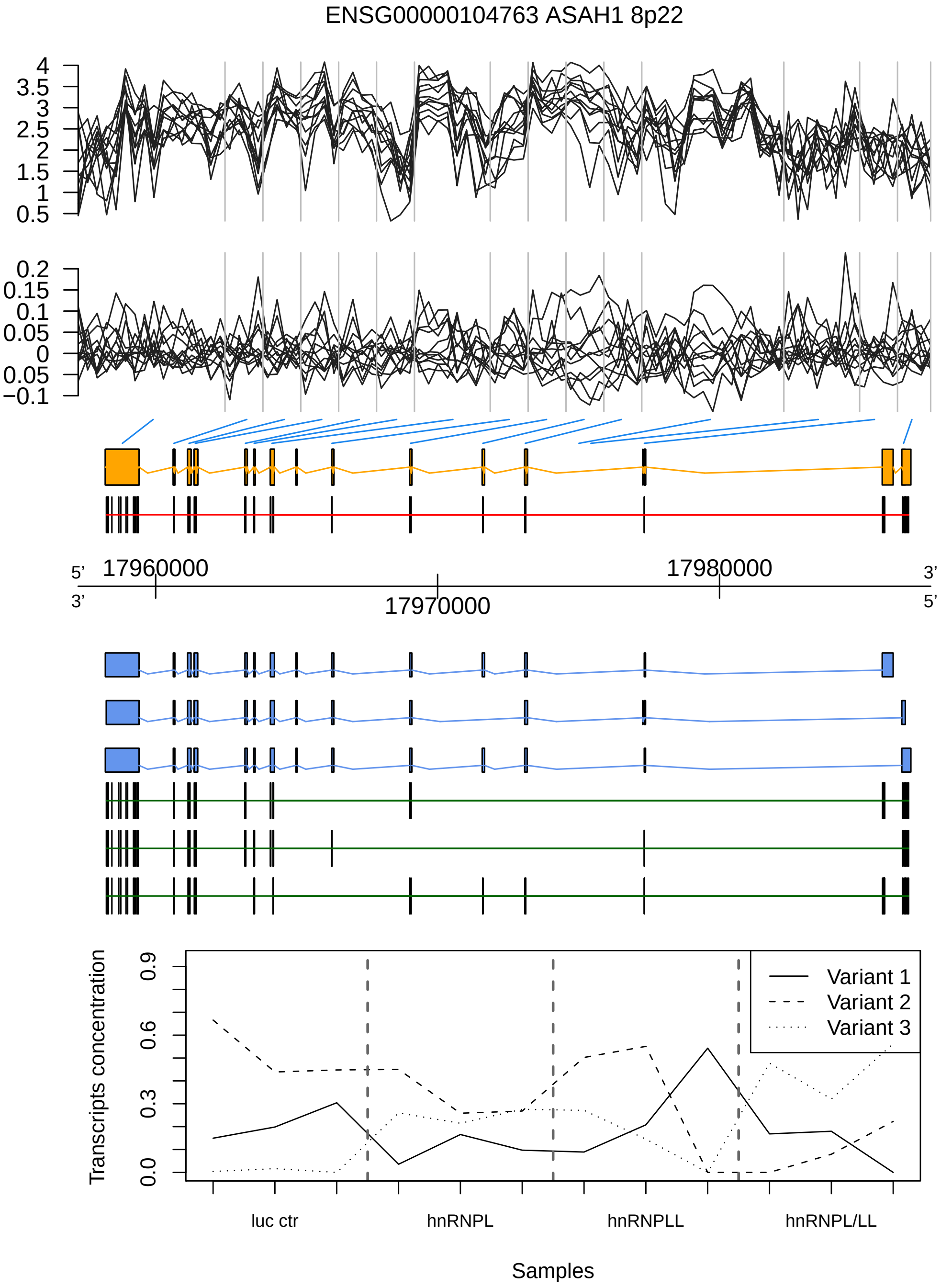

Figure 41: Results for ASAH1 gene (data from GSE8945) (Hung *et al.*, 2008). hnRNP L has been identified in the cited reference as a global regulator of AS. hnRNP LL is a paralog of hnRNP. Samples consist of three technical replicates of each condition: luciferase control, hnRNP L, hnRNP LL and hnRNP L/LL. hnRNP L, hnRNP LL and hnRNP L/LL are knockouts of hnRNP L, hnRNP LL and hnRNP L and LL respectively. In the reference, relative abundance of each of the isoforms (in RT-PCR) are quite similar to each other, and therefore difficult to distinguish using SPACE. Residues does not show any clear event to pinpoint.
