# Supplementary material for: Improvements to previous algorithms to predict gene structure and isoform concentrations using Affymetrix Exon arrays
Source: BMC Bioinformatics. 2010 Nov 26;11:578. doi: 10.1186/1471-2105-11-578 (PMC3012675; doi:10.1186/1471-2105-11-578)
Supplement: Additional file 3 — R package of SPACE algorithm for Affymetrix exon arrays. SPACE Binary R package to be used in Windows Platforms. [file 1471-2105-11-578-S3.ZIP › SPACE/html/00Index.html]

R: An algorithm to predict and quantify alternatively spliced
isoforms using microarrays

# An algorithm to predict and quantify alternatively spliced isoforms using microarrays


---

## Documentation for package ‘SPACE’ version 1.0

- DESCRIPTION file.

## Help Pages

|  |  |
| --- | --- |
| SPACE-package | An algorithm to predict and quantify alternatively spliced isoforms using microarrays |
| AdaptWH | Modification of the predicted structure matrix to make it closer to the shape of a real structure |
| CheckGmatrix | CheckGmatrix. Test the identifiability of a G matrix. |
| ComputeGmatrix | Compute the G matrix that relates genes and their transcripts. |
| estimateNTranscripts | Compute the estimated number of transcripts for a gene |
| fit.IsoformPlm | Performs the summarization of a dataset. |
| getProbeAffinityList.IsoformPlm | Get the estimated affinities for a set of units. |
| getTranscriptConcentrationList.IsoformPlm | Get the concentrations of the predicted (or given) isoforms. |
| getTranscriptConcentrationMatrix.IsoformPlm | Get the concentrations of the predicted (or given) isoforms. |
| getTranscriptStructureList.IsoformPlm | Get the predicted (or given) structure of the genes. |
| IsoformPlm | Creates the isoform object |
| nmfbeta | Non negative matrix factorization (NMF) of the input matrix. |
| RefineGmatrix | Applies the coherence algorithm over the estimated incidence matrix using information on probe locations. |
| removeOutliersGeneric | Removes outliers in matrix containing probe signals. |
| resortTranscripts | Change the order of the columns of a matrix. |
| SPACE | An algorithm to predict and quantify alternatively spliced isoforms using microarrays |
