# Supplementary material for: Improvements to previous algorithms to predict gene structure and isoform concentrations using Affymetrix Exon arrays
Source: BMC Bioinformatics. 2010 Nov 26;11:578. doi: 10.1186/1471-2105-11-578 (PMC3012675; doi:10.1186/1471-2105-11-578)
Supplement: Additional file 6 — Manual of the SPACE R package. Manual of the SPACE R package. [file 1471-2105-11-578-S6.PDF]

Package ‘SPACE’

September 27, 2010

Type Package

Title An algorithm to predict and quantify alternatively spliced isoforms using microarrays

Version 1.0

Date 2010-07-08

Author Miguel Angel and Angel Rubio

Maintainer Ander Aramburu <aaramburu@ceit.es>

Description SPACE is an algorithm that has been developed to estimate the number of different transcripts expressed under several conditions, predict the precursor mRNA splicing structure and quantify the transcript concentrations including unknown forms. The results presented here show its robustness and accuracy for real and simulated data.

Enhances base, R.utils

Depends aroma.affymetrix, MASS, matrixStats, graph, combinat

License GPL (version 2 or later)

R topics documented:

SPACE-package 2
AdaptWH 5
CheckGmatrix 6
ComputeGmatrix 7
estimateNTranscripts 8
fit.IsoformPlm 9
getProbeAffinityList.IsoformPlm 11
getTranscriptConcentrationList.IsoformPlm 12
getTranscriptConcentrationMatrix.IsoformPlm 13
getTranscriptStructureList.IsoformPlm 15
IsoformPlm 16
nmfbeta 18
RefineGmatrix 19
removeOutliersGeneric 19
resortTranscripts 20

Index **22**

`SPACE-package` *An algorithm to predict and quantify alternatively spliced isoforms using microarrays*

Description

SPACE is an algorithm that has been developed to estimate the number of different transcripts expressed under several conditions, predict the precursor mRNA splicing structure and quantify the transcript concentrations including unknown forms.

Details

Package:	SPACE
Type:	Package
Version:	0.1
Date:	2010-07-08
Enhances:	base, R.utils
Depends:	aroma.affymetrix, MASS, matrixStats, graph, combinat
License:	GPL (version 2 or later)

Author(s)

Miguel Anton, Ander Aramburu and Angel Rubio

Maintainer: Ander Aramburu <aaramburu@ceit.es>

References

SPACE: an algorithm to predict and quantify alternatively spliced isoforms using microarrays

Miguel A Anton, Dorleta Gorostiaga, Elizabeth Guruceaga, Victor Segura, Pedro Carmona-Saez, Alberto Pascual-Montano, Ruben Pio, Luis M Montuenga and Angel Rubio

See Also

http://www.aroma-project.org

Examples

```
# load SPACE package
# aroma.affymetrix package will also be loaded
library(SPACE)


# Set a proper directory to work with aroma.affymetrix
# From this directory there should be several directories such as
# annotationData, rawData, etc.
setwd("E:/workingDir")

# Download custom cdfs for Ensembl from
```

```
# http://brainarray.mbni.med.umich.edu/Brainarray/Database/CustomCDF/CDF_download.asp

# Go to last version (version 13) of the cdf files
# Click on ENSG, ENST and ENSE versions

# Go to Homo_sapiens and HuEx10stv2 (Affymetrix exon array) row
# Click on the last option CDF/Seq/Map/Desc to download each file
# Extract the cdf files from zip compresed files

# Brainarray cdf files are in ascii format
# They should be converted into binary format to be used with aroma.affymetrix
# using the convertCdf command (It will take time)
# This conversion should be done only once
#convertCdf("HuEx10stv2_Hs_ENSG.cdf", "HuEx-1_0-st-v2,ENSG,brainarray,v13.cdf")
#convertCdf("HuEx10stv2_Hs_ENST.cdf", "HuEx-1_0-st-v2,ENST,brainarray,v13.cdf")
#convertCdf("HuEx10stv2_Hs_ENST.cdf", "HuEx-1_0-st-v2,ENST,brainarray,v13.cdf")


#Download Affymetrix exon array sample dataset for human tissues from Affymetrix
#Only 6 samples will be used corresponding to kidney and liver tissues
#Once all the files have been downloaded, the must be arrange in the
#following directory structure

#D:/workingDir
#       /annotationData
#            /chipTypes
#                 /HuEx-1_0-st-v2
#                      HuEx-1_0-st-v2,ENSG,brainarray,v13.cdf
#                      HuEx-1_0-st-v2,ENST,brainarray,v13.cdf
#                      HuEx-1_0-st-v2,ENSE,brainarray,v13.cdf
#       /rawData
#            /humanLiverKidney
#                 /HuEx-1_0-st-v2
#                      huex_wta_kidney_A.CEL
#                      huex_wta_kidney_B.CEL
#                      huex_wta_kidney_C.CEL
#                      huex_wta_liver_A.CEL
#                      huex_wta_liver_B.CEL
#                      huex_wta_liver_C.CEL


# Background correction and normalization using aroma.affymetrix

verbose <- Arguments$getVerbose(-8)
timestampOn(verbose)

projectName <- "humanLiverKidney"
chipType <- "HuEx-1_0-st-v2"
cdfGeneFile <- "HuEx-1_0-st-v2,ENSG,brainarray,v13"
cdfTranscriptFile <- "HuEx-1_0-st-v2,ENST,brainarray,v13"
cdfExonFile <- "HuEx-1_0-st-v2,ENSE,brainarray,v13"

cdfG <- AffymetrixCdfFile$byChipType(cdfGeneFile)
cdfT <- AffymetrixCdfFile$byChipType(cdfTranscriptFile)
cdfE <- AffymetrixCdfFile$byChipType(cdfExonFile)

cs <- AffymetrixCelSet$byName(projectName, cdf=cdfG)
```

```
bc <- NormExpBackgroundCorrection(cs, method="mle", tag=c("*","ensembl"));
csBC <- process(bc,verbose=verbose);

qn <- QuantileNormalization(csBC, typesToUpdate="pm")
csN <- process(qn,verbose=verbose)

# Analysis with LiWangWong method
isoPlmLWW <- IsoformPlm(csN,
                        method="LiWangWong",
                        cdfT=cdfTranscriptFile,
                        cdfE=cdfExonFile,
                        maxIter=2000,
                        fileName="LWWTEST")

print("isoPlm object constructed")
outLWW <- fit(isoPlmLWW,verbose=TRUE)
# Last function actually performs the summarization.

TListLWW <- getTranscriptConcentrationList(isoPlmLWW)
AListLWW <- getProbeAffinityList(isoPlmLWW)
GListLWW <- getTranscriptStructureList(isoPlmLWW, Gmatrix="G")
GcListLWW <- getTranscriptStructureList(isoPlmLWW, Gmatrix="Gc")
TMatrixLWW <- getTranscriptConcentrationMatrix(isoPlmLWW)


# Analysis with SPACE method
# Estimating the number of transcripts
isoPlmSPACEest <- IsoformPlm(csN,
                             method="SPACE",
                             nbrOfTranscripts="estimate",
                             cdfT=cdfTranscriptFile,
                             cdfE=cdfExonFile,
                             fileName="SPACETEST")

print("isoPlm object constructed")
outSPACEest <- fit(isoPlmSPACEest,verbose=TRUE)
# Last function actually performs the summarization.

TListSPACEest <- getTranscriptConcentrationList(isoPlmSPACEest)
AListSPACEest <- getProbeAffinityList(isoPlmSPACEest)
GListSPACEest <- getTranscriptStructureList(isoPlmSPACEest, Gmatrix="G")
GcListSPACEest <- getTranscriptStructureList(isoPlmSPACEest, Gmatrix="Gc")
TMatrixSPACEest <- getTranscriptConcentrationMatrix(isoPlmSPACEest)

# If it is only needed for some genes
isoPlmSPACEestGenes <- IsoformPlm(csN,method="SPACE",nbrOfTranscripts="estimate",
                                  cdfT=cdfTranscriptFile,cdfE=cdfExonFile,
                                  fileName="SPACETEST_Genes")
outSPACEestGenes <- fit(isoPlmSPACEestGenes,verbose=TRUE,units=c(186,196,241))
TMatrixSPACEestGenes <- getTranscriptConcentrationMatrix(isoPlmSPACEestGenes)

# Get the unit that corresponds to an Ensembl identifier
isoPlmSPACEestEnsembl <- IsoformPlm(csN,method="SPACE",nbrOfTranscripts="estimate",
                                    cdfT=cdfTranscriptFile,cdfE=cdfExonFile,
                                    fileName="SPACETEST_Ensembl")
unit <- match("ENSG00000005302_at",getUnitNames(cdfG))
```

```
outSPACEestEnsembl <- fit(isoPlmSPACEestEnsembl,units=unit,verbose=TRUE)
TMatrixSPACEestEnsembl <- getTranscriptConcentrationMatrix(isoPlmSPACEestEnsembl)


# Analysis with SPACE method
# Number of transcripts for each gene equal to number of transcripts in Ensembl

isoPlmSPACEcdf <- IsoformPlm(csN,
                             method="SPACE",
                             nbrOfTranscripts="cdf",
                             cdfT=cdfTranscriptFile,
                             cdfE=cdfExonFile,
                             fileName="SPACETEST")
outSPACEcdf <- fit(isoPlmSPACEcdf,verbose=TRUE)

TListSPACEcdf <- getTranscriptConcentrationList(isoPlmSPACEcdf)
AListSPACEcdf <- getProbeAffinityList(isoPlmSPACEcdf)
GListSPACEcdf <- getTranscriptStructureList(isoPlmSPACEcdf, Gmatrix="G")
GcListSPACEcdf <- getTranscriptStructureList(isoPlmSPACEcdf, Gmatrix="Gc")
TMatrixSPACEcdf <- getTranscriptConcentrationMatrix(isoPlmSPACEcdf)


# Analysis with SPACE method
# Number of transcripts for each gene equal to 2

isoPlmSPACE2 <- IsoformPlm(csN,
                           method="SPACE",
                           nbrOfTranscripts=2,
                           cdfT=cdfTranscriptFile,
                           cdfE=cdfExonFile,
                           fileName="SPACETEST")
outSPACE2 <- fit(isoPlmSPACE2,verbose=TRUE)

TListSPACE2 <- getTranscriptConcentrationList(isoPlmSPACE2)
AListSPACE2 <- getProbeAffinityList(isoPlmSPACE2)
GListSPACE2 <- getTranscriptStructureList(isoPlmSPACE2, Gmatrix="G")
GcListSPACE2 <- getTranscriptStructureList(isoPlmSPACE2, Gmatrix="Gc")
TMatrixSPACE2 <- getTranscriptConcentrationMatrix(isoPlmSPACE2)
```

`AdaptWH`	*Modification of the predicted structure matrix to make it closer to the shape of a real structure*

Description

Compute a diagonal matrix D from a non-negative W matrix. The product W·D provides a result closer to the structure of a A·G matrix.

This is done by solving a system of equations robustly.

Usage

```
AdaptWH(W)
```

Arguments

`W` W matrix (structure of the gene)

Value

Provides a list with two elements (Wa and Diag) that correspond to the rescaled structure matrix and a vector of affinities respectively. Diag: Affinity vector of each of the probes. The product of the affinities is one. Wa: Rescaled structure.

$W_a = W \cdot diag(Diag)$

Examples

```
# Real G matrix
G <- matrix(c(rep(1,10), rep(1,8),rep(0,2), rep(0,3),rep(1,7)), 10,3)

# Estimated matrix.
# The example of a random matrix related with G.

Gnmf <- G + abs(matrix(.5*rnorm(10 * 3),10,3));
Gnmf <- Gnmf / apply(Gnmf,1,max);

Wnmf <- t(c(100,200,400) * t(Gnmf));

matplot(Wnmf, type="b")

# It is needed to rescale the Wnmf matrix
Wout <- AdaptWH(Wnmf);

# The new matrix shows a structure closer to G.
str(Wout)
matplot(Wout$Wa, type = "b")
```

`CheckGmatrix` *CheckGmatrix. Test the identifiability of a G matrix.*

Description

This is an internal function of the SPACE package. It is not expected to be used by the end user. It performs several checks over the matrix of incidence that relates genes and their transcripts. They are 1) Check that all the transcripts have different hybridization patterns. 2) Check the rank of the matrix 3) Check the identifiability.

Usage

```
CheckGmatrix(Glist, CheckComponents = TRUE)
```

Arguments

`Glist` List of G matrices that relates genes with transcripts.

`CheckComponents` It checks the identifiability of each of the matrices. Usually, this check is done to relate transcripts with genes and is not needed to related genes with exons.

Details

The identifiability is performed using the algorithm suggested by Hiller et al [2009].

Value

List of G's. Returns a modified list with the G's for every gene (several transcripts can be merged into a single one), additional fields (lack of identifiability, that is termed AGT unique) and the strong components of the solution. If the gene is not identifiable, the affinity of the sets of probes in the same strong component can be multiplied by any arbitrary factor.

References

Identifiability of isoform deconvolution from junction arrays and RNA-Seq, David Hiller, Hui Jiang, Weihong Xu and Wing Hung Wong. Bioinformatics 2009.

Examples

```
# An identifiable G matrix
# Note: The rows and the columns must have explicit names.
G1 <- cbind(c(rep(0,3), rep(1,7)), c(rep(1,7), rep(0,3)));
colnames(G1) <- c("G1_T1", "G1_T2");
rownames(G1) <- 1:10;
# A non-identifiable G matrix
G2 <- cbind(c(rep(0,3), rep(1,7)), c(rep(1,7), rep(1,3)));
colnames(G2) <- c("G2_T1", "G2_T2");
rownames(G2) <- 1:10;

Gmatrices <- list(G1, G2);
names(Gmatrices) <- c("Gene 1", "Gene 2")

# Check identifiabilty
Goutput <- CheckGmatrix(Gmatrices, CheckComponents = TRUE)
```

ComputeGmatrix *Compute the G matrix that relates genes and their transcripts.*

Description

This is an internal function of the SPACE package. It is not expected to be used by the end user. It computes the matrices that relate genes with transcripts (or with exons) given a pair of cdf objects: one for the genes and the other for the transcripts of exons.

Usage

```
ComputeGmatrix(cdfG, cdfT, patternG = "", patternT = "")
```

Arguments

`cdfG`	cdf object for genes
`cdfT`	cdf object for transcripts (or exons).
`patternG`	Pattern (regular expression) to select the genes within the gene cdf object. It can be useful to skip set of probes used only for normalization.
`patternT`	Pattern (regular expression) to select the genes within the transcript or exon cdf object. It can be useful to skip set of probes used only for normalization.

Value

list of G's. Returns a list with the G's for every gene

`estimateNTranscripts` *Compute the estimated number of transcripts for a gene*

Description

It provides the internal dimension of a SVD factorization using bi-cross validation based on an algorithm developed by Owen et al [2009].

Usage

```
estimateNTranscripts(X, Permutations = 250)
```

Arguments

`X`	X intensity matrix (probes x samples)
`Permutations`	Number of permutations to perform the cross-validation. The default value is 250.

Details

Even though, the estimated number of transcripts is performed using the SVD (for sake of computation time), results are also valid for NMF (if used for probe signal data).

Value

A list that returns the estimated number of transcripts, the median error of the bi-cross validation for each of the internal dimension and the value of the error for each of the L permutations.

References

Bi-cross-validation of the SVD and the nonnegative matrix factorization, Art B. Owen and Patrick O. Perry, Ann. Appl. Stat. Volume 3, Number 2 (2009), 564-594.

Examples

```
## Assuming that the internal dimension is four.
W <- matrix(runif(100 * 4), 100,4);
H <- matrix(runif(4 * 50), 4,50);

# Building the matrix
# Y <- W %*% H;
Y <- crossprod(t(W), H);

# Adding some noise
Y <- Y + 0.1 * matrix(rnorm(100 * 50),100,50);

# Estimating the internal dimension
nY <- estimateNTranscripts(Y)

# The internal dimension is, by construction, 4.
# The expected internal dimension is:
print(nY[[1]])
```

`fit.IsoformPlm` *Performs the summarization of a dataset.*

Description

Fits the SPACE or the LWW method to a set of data. The output for SPACE are the probe affinities, the predicted structure and the estimated concentrations. In the case of LWW the estimation of the concentrations. It also provides whether the gene is identifiable or not.

Usage

```
fit.IsoformPlm(this,units="remaining",...,force=FALSE,ram=1,verbose=FALSE)
```

Arguments

`this`	IsoPlm object to perform the summarization.
`units`	Units of the object to perform the summarization. The default value is "remaining", i.e., the summarization is performed for the genes that have not been summarized yet. The number of a particular unit can also be specified. See the aroma.affymetrix documentation for additional information on this function.
`...`	Additional parameters to be sent to the inherited objects.
`force`	If TRUE, the summarization must be performed even for the already summarized data.
`ram`	Memmory space to use in the summarization. Higher values give faster results. Small values use less RAM and hard disk is more strongly used. The default value 1 works well in a computer with 1Gb of RAM.
`verbose`	If TRUE, the current status of the algorithm's computation is displayed.

Details

In the LWW method, if the gene is not identificable the algorithm assumes that the mean of the affinities within a group of probes that correspond to the same connected component is identical.

Examples

```
library(SPACE)
# Background correction and normalization using aroma.affymetrix

setwd("E:/workingDir")

verbose <- Arguments$getVerbose(-8)
timestampOn(verbose)

projectName <- "humanTissues"
chipType <- "HuEx-1_0-st-v2"
cdfGeneFile <- "HuEx-1_0-st-v2,ENSG,brainarray,v13"
cdfTranscriptFile <- "HuEx-1_0-st-v2,ENST,brainarray,v13"
cdfExonFile <- "HuEx-1_0-st-v2,ENSE,brainarray,v13"

cdfG <- AffymetrixCdfFile$byChipType(cdfGeneFile)
cdfT <- AffymetrixCdfFile$byChipType(cdfTranscriptFile)
cdfE <- AffymetrixCdfFile$byChipType(cdfExonFile)

cs <- AffymetrixCelSet$byName(projectName, cdf=cdfG)

bc <- NormExpBackgroundCorrection(cs, method="mle", tag=c("*","ensembl"));
csBC <- process(bc,verbose=verbose);

qn <- QuantileNormalization(csBC, typesToUpdate="pm")
csN <- process(qn,verbose=verbose)

# Analysis with LiWangWong algorithm
isoPlm <- IsoformPlm(csN,
                     cdfT=cdfTranscriptFile,
                     cdfE=cdfExonFile,
                     method="LiWangWong",
                     maxIter=2000,
                     fileName="LWWTEST")

print("isoPlm object constructed")
out <- fit(isoPlm,verbose=TRUE,ram=1)
# Last function actually performs the summarization.


# Analysis with SPACE algorithm
isoPlm <- IsoformPlm(csN,
                     method="SPACE",
                     nbrOfTranscripts="estimate",
                     cdfT=cdfTranscriptFile,
                     cdfE=cdfExonFile,
                     maxIter=2000,
                     fileName="SPACETEST")

print("isoPlm object constructed")
out <- fit(isoPlm,verbose=TRUE,ram=1)
# Last function actually performs the summarization.

TList <- getTranscriptConcentrationList(isoPlm)
AList <- getProbeAffinityList(isoPlm)
```

```
GList <- getTranscriptStructureList(isoPlm, Gmatrix="G")
GcList <- getTranscriptStructureList(isoPlm, Gmatrix="Gc")
TMatrix <- getTranscriptConcentrationMatrix(isoPlm)
```

`getProbeAffinityList.IsoformPlm`

Get the estimated affinities for a set of units.

Description

Get the estimated affinities for a set of units. These are provided as a list. Each element of the list is a vector that corresponds to a gene. The number of elements of each vector represents the number of probes assigned to each gene.

Usage

```
getProbeAffinityList.IsoformPlm(this, ...)
```

Arguments

`this`	Name of the created object with the summarizations.
`...`	Additional arguments.

Value

Returns a list. The number of elements of the list is the number of genes. Each element contains as many affinity values as probes.

Examples

```
library(SPACE)

setwd("E:/workingDir")

verbose <- Arguments$getVerbose(-8)
timestampOn(verbose)

projectName <- "humanTissues"
chipType <- "HuEx-1_0-st-v2"
cdfGeneFile <- "HuEx-1_0-st-v2,ENSG,brainarray,v13"
cdfTranscriptFile <- "HuEx-1_0-st-v2,ENST,brainarray,v13"
cdfExonFile <- "HuEx-1_0-st-v2,ENSE,brainarray,v13"

cdfG <- AffymetrixCdfFile$byChipType(cdfGeneFile)
cdfT <- AffymetrixCdfFile$byChipType(cdfTranscriptFile)
cdfE <- AffymetrixCdfFile$byChipType(cdfExonFile)

cs <- AffymetrixCelSet$byName(projectName, cdf=cdfG)

# Background correction and normalization using aroma.affymetrix
bc <- NormExpBackgroundCorrection(cs, method="mle", tag=c("*","ensembl"));
csBC <- process(bc,verbose=verbose);
```

```
qn <- QuantileNormalization(csBC, typesToUpdate="pm")
csN <- process(qn,verbose=verbose)

# Analysis with LiWangWong algorithm
isoPlm <- IsoformPlm(csN,
        method="LiWangWong",
        cdfT=cdfTranscriptFile,
        cdfE=cdfExonFile,
        maxIter=2000,
        fileName="LWWTEST")

print("isoPlm object constructed")
out <- fit(isoPlm,units = c(1,2,3),verbose=TRUE,ram=.2)
# Last function actually performs the summarization.

AList <- getProbeAffinityList(isoPlm)
```

`getTranscriptConcentrationList.IsoformPlm`
Get the concentrations of the predicted (or given) isoforms.

Description

This function gives as output a list with the estimated concentrations of all the transcripts within the provided experiment by the argument. Each element of the list contains the concentrations of the transcripts that correspond to a gene.

Usage

```
getTranscriptConcentrationList.IsoformPlm(this, ...)
```

Arguments

`this`	Fitted object that have been summarizaed using SPACE.
`...`	Additional parameters to other objects.

Value

A list with as many elements as units. Each element contains the expression matrix that correspond to the trasncripts.

Examples

```
library(SPACE)

setwd("E:/workingDir")

verbose <- Arguments$getVerbose(-8)
timestampOn(verbose)

projectName <- "humanTissues"
```

```
chipType <- "HuEx-1_0-st-v2"
cdfGeneFile <- "HuEx-1_0-st-v2,ENSG,brainarray,v13"
cdfTranscriptFile <- "HuEx-1_0-st-v2,ENST,brainarray,v13"
cdfExonFile <- "HuEx-1_0-st-v2,ENSE,brainarray,v13"

cdfG <- AffymetrixCdfFile$byChipType(cdfGeneFile)
cdfT <- AffymetrixCdfFile$byChipType(cdfTranscriptFile)
cdfE <- AffymetrixCdfFile$byChipType(cdfExonFile)

cs <- AffymetrixCelSet$byName(projectName, cdf=cdfG)

# Background correction and normalization using aroma.affymetrix
bc <- NormExpBackgroundCorrection(cs, method="mle", tag=c("*","ensembl"));
csBC <- process(bc,verbose=verbose);

qn <- QuantileNormalization(csBC, typesToUpdate="pm")
csN <- process(qn,verbose=verbose)

# Analysis with LiWangWong algorithm
isoPlm <- IsoformPlm(csN,
        method="LiWangWong",
        cdfT=cdfTranscriptFile,
        cdfE=cdfExonFile,
        maxIter=2000,
        fileName="LWWTEST")

# Assuming that there is an object isoPlm.
out <- fit(isoPlm,units = c(1,2,3),verbose=TRUE,ram=.2)
transcriptList <- getTranscriptConcentrationList(isoPlm)
```

---

getTranscriptConcentrationMatrix.IsoformPlm

Get the concentrations of the predicted (or given) isoforms.

Description

This function provides a matrix with the estimated concentrations of all the transcripts within the experiment provided by the argument. Each row of the matrix contains the estimated concentration of a particular transcript in the different samples.

Usage

```
getTranscriptConcentrationMatrix.IsoformPlm(this, ...)
```

Arguments

`this`	Fitted object that have been summarizaed using SPACE.
`...`	Additional parameters to other objects.

Details

The names of the rows (i.e. the transcripts) are different depending on the algorithm for summarization. If the algorithm is LWW, then the names of the rows are the names of the transcripts provided by the cdf file. If the algorithm is SPACE, then the names of the rows are the names of the genes appended by a dash, the number of the transcript and the total number of transcripts.

Value

A matrix for whose size is transcripts times samples.

Examples

```
library(SPACE)

setwd("E:/workingDir")

verbose <- Arguments$getVerbose(-8)
timestampOn(verbose)

projectName <- "humanTissues"
chipType <- "HuEx-1_0-st-v2"
cdfGeneFile <- "HuEx-1_0-st-v2,ENSG,brainarray,v13"
cdfTranscriptFile <- "HuEx-1_0-st-v2,ENST,brainarray,v13"
cdfExonFile <- "HuEx-1_0-st-v2,ENSE,brainarray,v13"

cdfG <- AffymetrixCdfFile$byChipType(cdfGeneFile)
cdfT <- AffymetrixCdfFile$byChipType(cdfTranscriptFile)
cdfE <- AffymetrixCdfFile$byChipType(cdfExonFile)

cs <- AffymetrixCelSet$byName(projectName, cdf=cdfG)

# Background correction and normalization using aroma.affymetrix
bc <- NormExpBackgroundCorrection(cs, method="mle", tag=c("*","ensembl"));
csBC <- process(bc,verbose=verbose);

qn <- QuantileNormalization(csBC, typesToUpdate="pm")
csN <- process(qn,verbose=verbose)

# Analysis with SPACE algorithm
isoPlm <- IsoformPlm(csN,
        method="SPACE",
        nbrOfTranscripts="estimate",
        cdfT=cdfTranscriptFile,
        cdfE=cdfExonFile,
        maxIter=3000,
        fileName="SPACETEST")

print("isoPlm object constructed")
out <- fit(isoPlm,units = c(1,2,3),verbose=TRUE,ram=.2)
# Last function actually performs the summarization.

transcriptMatrix <- getTranscriptConcentrationMatrix(isoPlm)
```

`getTranscriptStructureList.IsoformPlm`
Get the predicted (or given) structure of the genes.

Description

This function gives output as a list with the estimated structure of all transcripts within the provided experiment. Each element of the list contains the structure of the transcripts that correspond to a gene.

Usage

```
getTranscriptStructureList.IsoformPlm(this, Gmatrix = "G", ...)
```

Arguments

`this`	Fitted object that have been summarized using SPACE.
`Gmatrix`	This parameter has two possible values: "G" and "Gc". For "G", it returns the estimated structure without taking into account the coherence algorithm. For "Gc", it returns the estimated structure using the information on which sets belong to the same indivisible part of an exon.
`...`	Additional parameters to other objects.

Examples

```
library(SPACE)

setwd("E:/workingDir")

verbose <- Arguments$getVerbose(-8)
timestampOn(verbose)

projectName <- "humanTissues"
chipType <- "HuEx-1_0-st-v2"
cdfGeneFile <- "HuEx-1_0-st-v2,ENSG,brainarray,v13"
cdfTranscriptFile <- "HuEx-1_0-st-v2,ENST,brainarray,v13"
cdfExonFile <- "HuEx-1_0-st-v2,ENSE,brainarray,v13"

cdfG <- AffymetrixCdfFile$byChipType(cdfGeneFile)
cdfT <- AffymetrixCdfFile$byChipType(cdfTranscriptFile)
cdfE <- AffymetrixCdfFile$byChipType(cdfExonFile)

cs <- AffymetrixCelSet$byName(projectName, cdf=cdfG)

# Background correction and normalization using aroma.affymetrix
bc <- NormExpBackgroundCorrection(cs, method="mle", tag=c("*","ensembl"));
csBC <- process(bc,verbose=verbose);

qn <- QuantileNormalization(csBC, typesToUpdate="pm")
csN <- process(qn,verbose=verbose)

# Analysis with SPACE algorithm
isoPlm <- IsoformPlm(csN,
```

```
          method="SPACE",
          nbrOfTranscripts="estimate",
          cdfT=cdfTranscriptFile,
          cdfE=cdfExonFile,
          maxIter=3000,
          fileName="SPACETEST")

print("isoPlm object constructed")

# It is only needed for some genes
out <- fit(isoPlm,verbose=TRUE,ram=1,units=c(186,196,241))

GList <- getTranscriptStructureList(isoPlm, Gmatrix="G")
GcList <- getTranscriptStructureList(isoPlm, Gmatrix="Gc")
```

IsoformPlm *Creates the isoform object*

Description

An isoform object is the main object to store the summarized value of the structure and the concentrations of the isoforms in an experiment. Using this function, these objects are created and, afterwards, applying the fit function, the summarization procedure is performed.

Usage

```
IsoformPlm(..., method = "SPACE",
                nbrOfTranscripts = "estimate",
                cdfT = cdfTranscriptFile,
                cdfE = cdfExonFile,
                LWWalgorithmA = "euclidean",
                LWWalgorithmT = "pseudoinverse",
                maxIter = NULL,
                fileName = "TEST")
```

Arguments

`method` Method: "SPACE" or "LiWangWong". "SPACE" predicts the structure and concentrations. "LiWangWong" estimates the concentrations given the structure. This structure is deduced from the cdf for genes and transcripts.

`nbrOfTranscripts` Number of transcripts. The possible values are: "estimate": The number of transcripts is estimated. "cdf": The number of transcripts is the same of the provided cdf for transcripts. <Numeric>: The number of trasncripts is fixed to a number. The last one is especially useful if the summarization is performed for a single gene.

`cdfT` cdf file for transcripts.

`cdfE` cdf file for exons.

`LWWalgorithmA` Type of algorithm to estimate the affinities for the LiWangWong algorithm. By default is "euclidean".

LWWalgorithmT
: Type of algorithm to estimate the concentrations for the LiWangWong algorithm. By default is "pseudoinverse".

maxIter
: Maximum number of iterations to converge. If NULL, SPACE method will converge in 3000 iterations and LiWangWong method in 1000 iterations.

fileName
: Name of the file to store the results. This name is appended with several tags depending on the selected options.

Value

An isoform object.

Examples

```
library(SPACE)

setwd("E:/workingDir")

verbose <- Arguments$getVerbose(-8)
timestampOn(verbose)

projectName <- "humanTissues"
chipType <- "HuEx-1_0-st-v2"
cdfGeneFile <- "HuEx-1_0-st-v2,ENSG,brainarray,v13"
cdfTranscriptFile <- "HuEx-1_0-st-v2,ENST,brainarray,v13"
cdfExonFile <- "HuEx-1_0-st-v2,ENSE,brainarray,v13"

cdfG <- AffymetrixCdfFile$byChipType(cdfGeneFile)
cdfT <- AffymetrixCdfFile$byChipType(cdfTranscriptFile)
cdfE <- AffymetrixCdfFile$byChipType(cdfExonFile)

cs <- AffymetrixCelSet$byName(projectName, cdf=cdfG)

# Background correction and normalization using aroma.affymetrix
bc <- NormExpBackgroundCorrection(cs, method="mle", tag=c("*","ensembl"));
csBC <- process(bc,verbose=verbose);

qn <- QuantileNormalization(csBC, typesToUpdate="pm")
csN <- process(qn,verbose=verbose)

# Analysis with wang algorithm
isoPlm <- IsoformPlm(csN,
        method="LiWangWong",
        cdfT=cdfTranscriptFile,
        cdfE=cdfExonFile,
        maxIter=2000,
        fileName="LWWTEST")

print("isoPlm object constructed for LiWangWong method")


# Analysis with SPACE algorithm
isoPlm <- IsoformPlm(csN,
                     method="SPACE",
                     nbrOfTranscripts="estimate",
                     cdfT=cdfTranscriptFile,
```

```
                        cdfE=cdfExonFile,
                        maxIter=2000,
                        fileName="SPACETEST")

print("isoPlm object constructed for SPACE method")
```

---

nmfbeta *Non negative matrix factorization (NMF) of the input matrix.*

Description

This function performs the NMF of the input matrix. It uses the "standard" algorithm provided by Lee and Seung [1999] and adapted by Cichocki et al. [2008] to modify the sparsity of the factors.

Usage

```
nmfbeta(V, alphaW, alphaH, coeff, rdim, maxIter = 3000, trace = FALSE)
```

Arguments

V	Input data matrix to be factorized so that $V \approx W \cdot H$.
alphaW	An exponent that increases the sparsity of the W factor. Typical values are from 0.99 to 1.01. Values larger than 1.0 increase sparsity. Values smaller than 1.0 tend to "fill" the W factor.
alphaH	An exponent with equivalent behaviour for the H factor to alphaW.
coeff	Type of distance to be used (0: Itakuro-Saito Divergence, 1: Kullback Leiver divergence, 2: euclidean norm. Intermediate values can also be used. Adapted from Kompass [2007].
rdim	Internal dimension of the factorization.
maxIter	Maximum number of iterations. Default value is 3000.
trace	Displays additional output of the algorithm.

Value

It return a list that includes:

W	W factor of the factorization
H	H factor of the factorization
iter	Number of iterations to get to the convergence.

References

Daniel D. Lee and H. Sebastian Seung (1999). "Learning the parts of objects by non-negative matrix factorization". Nature 401 (6755): 788–791. doi:10.1038/44565. PMID 10548103.

Raul Kompass (March 2007). "A Generalized Divergence Measure for Nonnegative Matrix Factorization". Neural Computation 19 (3): 780–791. doi:10.1162/neco.2007.19.3.780. PMID 17298233.

Andrzej Cichocki, Rafal Zdunek and Shun-ichi Amari (January 2008). "Nonnegative Matrix and Tensor Factorization". IEEE Signal Processing Magazine 25 (1): 142–145. doi:10.1109/MSP.2008.4408452.

RefineGmatrix — *Applies the coherence algorithm over the estimated incidence matrix using information on probe locations.*

Description

This function is not intended to be used by the end user. It combines the information of the matrix that relates genes with exons to create sets of probes that must behave coherently. All the values within the same exon are assigned to the same corresponding value. In the case of alternative donor or acceptor sites, each part of exon is treated separately.

Usage

```
RefineGmatrix(Gnmf, GE, Anmf = as.vector(matrix(1, ncol = (dim(GE)[1]))))
```

Arguments

Gnmf	Initial estimate of the G matrix
GE	Matrix that relates genes with exons.
Anmf	Affinities. It is used to improve performance since probes with large affinities are more reliable.

Value

Corrected G matrix

removeOutliersGeneric — *Removes outliers in matrix containing probe signals.*

Description

Removes outliers in matrix containing Expression signals. The identification of the outliers is done by analyzing the residues of the median polish method. If the residues are larger than a number of standard deviations, the value is also substituted by an estimation using the median polish method.

Usage

```
removeOutliersGeneric(Y, tau=6, algorithm="median", permutations=250, ...)
```

Arguments

Y	An IxK matrix. All its entries must be positive.
tau	A scalar specifying the threshold (number of standard deviations) to identify outliers.
algorithm	Type of algorithm to remove the outliers. Two versions are implemented: “median”, uses the medianPolish algortihm and checks for large residuals. It is fast. “BCV” uses an adaptation the bi-cross validation (Owen[2009]). It is more accurate but slower.

tau	A scalar specifying the threshold (number of standard deviations) to identify outliers.
...	Not used

Value

Returns an IxK matrix where outliers have been “pruned”. Outliers are substituted by an estimate of the true value.

Examples

```
## Generate the signal probes.

W <- matrix(runif(100 * 4), 100,4);
H <- matrix(runif(4 * 50), 4,50);

# Build the matrix
# Yinit <- W %*% H;
Yinit <- crossprod(t(W),H)

# Add some noise
Y <- Yinit + 0.1 * matrix(rnorm(100 * 50),100,50);
Y <- (Y>0) * Y + 1e-3;

# Add some outliers
Y[10,20] <- 1e4;
Y[15,25] <- 1e3;

# Remove Outliers with the median algorithm
Yromedian <- removeOutliersGeneric(Y, algorithm = "median")
# The imputed values should be much smaller
print(c(Yromedian[10,20],Y[10,20]))
print(c(Yromedian[15,25],Y[15,25]))

# There also some other values that have been changed
sum(abs(Yromedian-Y)> 1e-10)

YroBCV <- removeOutliersGeneric(Y, algorithm = "BCV")
# The imputed values should be much smaller
print(c(YroBCV[10,20],Y[10,20]))
print(c(YroBCV[15,25],Y[15,25]))

# Much less "good" values were changed.
sum(abs(YroBCV-Y)> 1e-10)
```

`resortTranscripts` *Change the order of the columns of a matrix.*

Description

This function is not intended for the end user. It finds -using a heuristic- the order of the transcripts that makes the estimated Gnmf closer to a reference G matrix. It is useful to match the predicted structure with information obtained from Ensembl or other sources.

Usage

```
resortTranscripts(G, Gnmf)
```

Arguments

`G`	Reference structure
`Gnmf`	Estimated structure

Details

It also works if both matrixes have different size. In this case, it finds the order of the n first rows from the largest matrix that better fit to other matrix.

Value

It returns a list that includes:

`ordenG`	A sorting vector so that Gnmf[,ordenG] is close to G
`G`	Provided reference matrix (perhaps resorted)
`ordenGnmf`	A sorting vector so that G[,ordenGnmf] is close to Gnmf
`Gnmf`	Provided estimated structure matrix (perhaps resorted)

Examples

```
# Reference matrix
G <- matrix(c(rep(1,10), rep(1,8),rep(0,2), rep(0,3),rep(1,7)), 10,3)

# Estimated matrix.
# for the example a random matrix related with G.

Gnmf <- G + abs(matrix(.5*rnorm(10 * 3),10,3));
Gnmf <- Gnmf / apply(Gnmf,1,max);

#Reorder Gnmf
Gnmf <- Gnmf[,c(2,3,1)];

# The result of the ordering vector should be 3,1,2
newOrder <- resortTranscripts(Gnmf, G)
print(newOrder$ordenG)
```

Index

∗Topic **package**
SPACE-package, 1

AdaptWH, 5

CheckGmatrix, 6
ComputeGmatrix, 7

estimateNTranscripts, 8

fit.IsoformPlm, 9

getProbeAffinityList.IsoformPlm, 10
getTranscriptConcentrationList.IsoformPlm, 12
getTranscriptConcentrationMatrix.IsoformPlm, 13
getTranscriptStructureList.IsoformPlm, 14

http://www.aroma-project.org, *2*

IsoformPlm, 15

nmfbeta, 17

RefineGmatrix, 18
removeOutliersGeneric, 19
resortTranscripts, 20

SPACE (*SPACE-package*), 1
SPACE-package, 1
